# Genome sequencing of 2000 canids by the Dog10K consortium advances the understanding of demography, genome function and architecture

**DOI:** 10.1186/s13059-023-03023-7

**Published:** 2023-08-15

**Authors:** Jennifer R. S. Meadows, Jeffrey M. Kidd, Guo-Dong Wang, Heidi G. Parker, Peter Z. Schall, Matteo Bianchi, Matthew J. Christmas, Katia Bougiouri, Reuben M. Buckley, Christophe Hitte, Anthony K. Nguyen, Chao Wang, Vidhya Jagannathan, Julia E. Niskanen, Laurent A. F. Frantz, Meharji Arumilli, Sruthi Hundi, Kerstin Lindblad-Toh, Catarina Ginja, Kadek Karang Agustina, Catherine André, Adam R. Boyko, Brian W. Davis, Michaela Drögemüller, Xin-Yao Feng, Konstantinos Gkagkavouzis, Giorgos Iliopoulos, Alexander C. Harris, Marjo K. Hytönen, Daniela C. Kalthoff, Yan-Hu Liu, Petros Lymberakis, Nikolaos Poulakakis, Ana Elisabete Pires, Fernando Racimo, Fabian Ramos-Almodovar, Peter Savolainen, Semina Venetsani, Imke Tammen, Alexandros Triantafyllidis, Bridgett vonHoldt, Robert K. Wayne, Greger Larson, Frank W. Nicholas, Hannes Lohi, Tosso Leeb, Ya-Ping Zhang, Elaine A. Ostrander

**Affiliations:** 1 Science for Life Laboratory, Department of Medical Biochemistry and Microbiology, Uppsala University, 75132 Uppsala, Sweden; 2 Department of Human Genetics, University of Michigan, Ann Arbor, MI 48107 USA; 3 State Key Laboratory of Genetic Resources and Evolution, Kunming Institute of Zoology, Chinese Academy of Sciences, Kunming, 650223 China; 4 National Human Genome Research Institute, National Institutes of Health, 50 South Drive, Building 50 Room 5351, Bethesda, MD 20892 USA; 5 Section for Molecular Ecology and Evolution, Globe Institute, University of Copenhagen, Øster Voldgade 5-7, 1350 Copenhagen, Denmark; 6 University of Rennes, CNRS, Institute Genetics and Development Rennes - UMR6290, 35000 Rennes, France; 7 Institute of Genetics, Vetsuisse Faculty, University of Bern, 3001 Bern, Switzerland; 8 Department of Medical and Clinical Genetics, Department of Veterinary Biosciences, University of Helsinki and Folkhälsan Research Center, 02900 Helsinki, Finland; 9 School of Biological and Behavioural Sciences, Queen Mary University of London, London E14NS, UK and Palaeogenomics Group, Department of Veterinary Sciences, Ludwig Maximilian University, D-80539 Munich, Germany; 10 Broad Institute of MIT and Harvard, Cambridge, MA 02142 USA; 11 BIOPOLIS-CIBIO-InBIO-Centro de Investigação Em Biodiversidade E Recursos Genéticos - ArchGen Group, Universidade Do Porto, 4485-661 Vairão, Portugal; 12 Department of Public Health, Udayana University, Bali, 80361 Indonesia; 13 Department of Biomedical Sciences, Cornell University, 930 Campus Road, Ithaca, NY 14853 USA; 14 Department of Veterinary Integrative Biosciences, School of Veterinary Medicine and Biomedical Sciences, Texas A&M University, College Station, TX 77843 USA; 15 Department of Genetics, School of Biology, ), Aristotle University of Thessaloniki, Thessaloniki, Macedonia 54124, Greece and Genomics and Epigenomics Translational Research (GENeTres), Center for Interdisciplinary Research and Innovation (CIRI-AUTH, Balkan Center, Thessaloniki, Greece; 16 NGO “Callisto”, Wildlife and Nature Conservation Society, 54621 Thessaloniki, Greece; 17 Natural History Museum of Crete & Department of Biology, University of Crete, 71202 Irakleio, Greece; 18 Biology Department, School of Sciences and Engineering, University of Crete, Heraklion, Greece; 19 Palaeogenomics and Evolutionary Genetics Lab, Institute of Molecular Biology and Biotechnology (IMBB), Foundation for Research and Technology - Hellas (FORTH), Heraklion, Greece; 20 Department of Gene Technology, Science for Life Laboratory, KTH - Royal Institute of Technology, 17121 Solna, Sweden; 21 Department of Genetics, School of Biology, Aristotle University of Thessaloniki, 54124 Thessaloniki, Macedonia Greece; 22 Sydney School of Veterinary Science, The University of Sydney, Sydney, NSW 2570 Australia; 23 Department of Ecology and Evolutionary Biology, Princeton University, Princeton, NJ 08544 USA; 24 Department of Ecology and Evolutionary Biology, Ecology and Evolutionary Biology, University of California, Los Angeles, CA 90095-7246 USA; 25 Palaeogenomics and Bio-Archaeology Research Network, School of Archaeology, University of Oxford, Oxford, OX1 3TG UK

**Keywords:** Canine, Dog, Genomics, Variation, Demographic history, Mitochondrial DNA, Genetic diversity

## Abstract

**Background:**

The international Dog10K project aims to sequence and analyze several thousand canine genomes. Incorporating 20 × data from 1987 individuals, including 1611 dogs (321 breeds), 309 village dogs, 63 wolves, and four coyotes, we identify genomic variation across the canid family, setting the stage for detailed studies of domestication, behavior, morphology, disease susceptibility, and genome architecture and function.

**Results:**

We report the analysis of > 48 M single-nucleotide, indel, and structural variants spanning the autosomes, X chromosome, and mitochondria. We discover more than 75% of variation for 239 sampled breeds. Allele sharing analysis indicates that 94.9% of breeds form monophyletic clusters and 25 major clades. German Shepherd Dogs and related breeds show the highest allele sharing with independent breeds from multiple clades. On average, each breed dog differs from the UU_Cfam_GSD_1.0 reference at 26,960 deletions and 14,034 insertions greater than 50 bp, with wolves having 14% more variants. Discovered variants include retrogene insertions from 926 parent genes. To aid functional prioritization, single-nucleotide variants were annotated with SnpEff and Zoonomia phyloP constraint scores. Constrained positions were negatively correlated with allele frequency. Finally, the utility of the Dog10K data as an imputation reference panel is assessed, generating high-confidence calls across varied genotyping platform densities including for breeds not included in the Dog10K collection.

**Conclusions:**

We have developed a dense dataset of 1987 sequenced canids that reveals patterns of allele sharing, identifies likely functional variants, informs breed structure, and enables accurate imputation. Dog10K data are publicly available.

**Supplementary Information:**

The online version contains supplementary material available at 10.1186/s13059-023-03023-7.

## Background

Recent advances in comparative genomics have enhanced the utility of the domestic dog and other canines for studies of mammalian biology, disease, and domestication. The initial dog reference genome, derived from a single boxer, was released in 2004 [1] and has since been augmented with reference patches [2, 3], variation catalogs (e.g., [4–6]), and functional annotations (e.g., [2, 3, 7]). The resulting data has been important for identifying genes and variants controlling simple Mendelian traits (e.g., [4, 8, 9]), tracing migration of human populations [10–12], building a vocabulary for mammalian behavior [13, 14], and enabling studies of both aging [15, 16] and disease susceptibility [17].

Many complex genetic questions remain, and answering them has been limited by the reliance of both reference and test datasets comprised of dogs of largely western European descent, incomplete catalogs of copy number variants [5], and the exclusion of village and feral dogs and other canid species from most datasets [18]. Exome-based sequencing approaches have made useful contributions, but have been limited by dataset size [19]. Also, while studies of ancient canids have revealed key events in canine history (e.g., [11, 20–24]), this research relies on high-quality reference genomes supported by sequence variation from large numbers of wild and domestic canids. At present, these resources are insufficient. In response to this demand, a group of canine geneticists and biologists joined forces in 2016 to initiate Dog10K, a worldwide consortium with a goal of producing and analyzing DNA sequences from 10,000 canids [25].

Since 2004, several hundred canine genomes have been partially or fully sequenced by individual groups or laboratories, most with the aim of amassing markers for genome-wide association studies (GWAS) and subsequent fine mapping and functional studies, or for inferring canine history. As the diversity, density, and quality of available sequences have improved, so too has the resolution for identifying putative functional variants, although these studies have not kept pace with the larger field of mammalian biology [4, 26, 27]. The publication of new, high-quality, long-read assemblies of the Basenji [28], Great Dane [29], German Shepherd Dog [30, 31], Labrador Retriever [32], a revised version of the original Boxer [33], dingo [34], and gray wolf [35] have aided the community’s effort to address historical topics of interest and permit the analysis of previously inaccessible genomic features such as gene promoters, regulatory elements, repeated sequences, and mobile elements [29, 30]. Although phase-resolved canine assemblies are not currently available, the continued development of long-read assemblies will enable future analyses of variation using a pangenome approach [36]. In this study, we discover and characterize canine variation through alignment of Illumina sequencing reads to the recently published assembly of Mischka, a German Shepherd Dog (UU_Cfam_GSD_1.0) [30].

The Dog10K dataset includes samples from 321 dog breeds, with 261 breeds represented by three or more individuals, containing a worldwide distribution of rare and common breeds, collectively spanning variation in morphology, disease susceptibility, and behavior. Our dataset uniquely possesses a worldwide sampling of village dogs and niche populations, both of which fall outside the umbrella of pure or mixed breed dogs. The inclusion of 1929 individuals makes the Dog10K reference panel the largest to date, allowing for the imputation of canine genotypes across diverse breeds and genotyping platforms, including low-pass sequencing data. Finally, the inclusion of wild canid populations, including wolves and coyotes, completes the most comprehensive and inclusive dataset of canines assembled, allowing us to perform detailed analyses of genome architecture.

In the analysis herein, we present Illumina sequencing data from 1987 canids, with joint calling across the mitochondrial and nuclear genomes revealing over 144,000 structural variants (deletions, insertions, duplications, and inversions ≥ 50 bp in size), 14.4 million indels, and 34 million single-nucleotide variants (SNVs), the most extensive variant catalog produced in canines to date. Clade analysis with the nuclear SNV dataset reveals both expected and new relationships among breed dogs sampled. The sequencing of > 330 village dogs and wolves demonstrates a wealth of variation previously undiscovered in breed dogs, with almost one third of all observed variation exclusive to these two groups. Analysis of mitochondrial data reveals surprisingly few haplotypes in dogs, with greater observed variation in wild canids.

## Results

### Sample selection and data harmonization

**Fig. 1:**
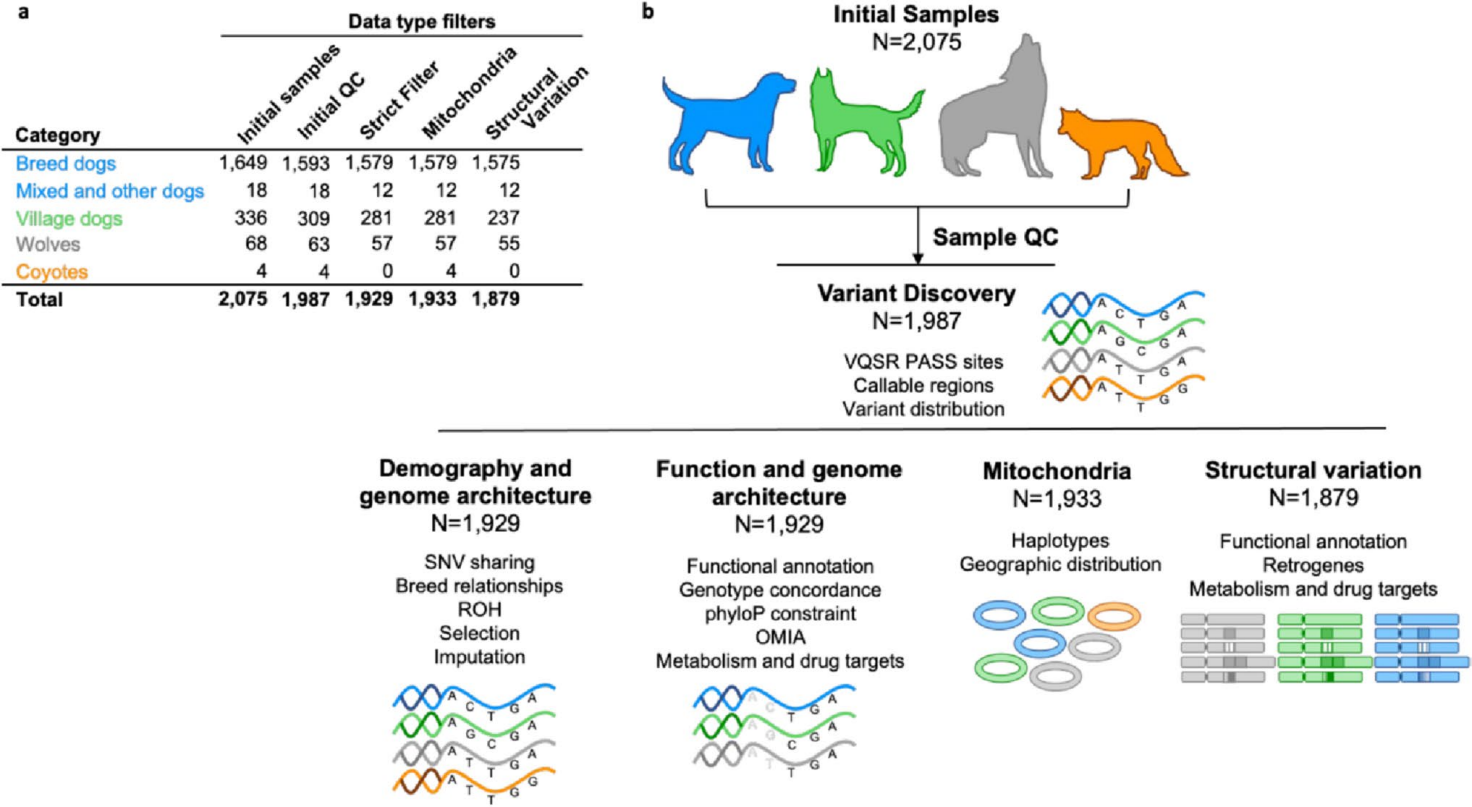
Overview of the Dog10K collection. **a** Sample collection and sample filtering for (**b**) the varied demographic, genome function, and architecture examined in the program. QC, quality control. ROH, runs of homozygosity. OMIA, Online Mendelian Inheritance in Animals

The 2075 samples collected for Dog10K were selected to represent a wide variety of breeds of differing morphology, history, and behavior (1649 samples); dogs that represent local niche populations or breeds that are not nationally registered (18 samples); village dogs from multiple locations (336 samples); and wild canids (68 wolves, 4 coyotes) (Fig. 1, Additional file 1: Table S1). At the time of collection, breed samples were free from known disease, and efforts were made to balance sex across all populations (52.6% female).

A reference genome consisting of the German Shepherd Dog genome assembly [30] (UU_Cfam_GSD_1.0, GCF_011100685.1), supplemented by three Y chromosome contigs from a Labrador Retriever (ROS_Cfam_1.0, GCF_014441545.1), was used as the foundation for all analyses. A pipeline based on bwa-mem2 and GATK best practices was used for the uniform sequence alignment and processing across four centers (Additional file 2: Sect. 1) [37–39]. Variant calling (mitochondrial genome: SNVs and indels; nuclear genome: SNVs, indels, and SVs) and quality filtering were performed across the entire sample set. Sample and variant filters were used to generate different datasets for addressing specific questions (Fig. 1).

In brief, primary SNV and small indel variant discovery was performed on 1987 samples fulfilling initial quality thresholds (Additional file 2: Sect. 2). Of these, 1929 samples passed additional quality control thresholds and were used in most SNV analyses. In these steps, variants were selected using either VQSR PASS criteria or additional strict variant filters (Additional file 2: Sect. 9). For SV analyses, 1824 samples were available after additional quality controls (Additional file 2: Sect. 7).

### Genome-wide pattern of sequence variation in canines

**Fig. 2:**
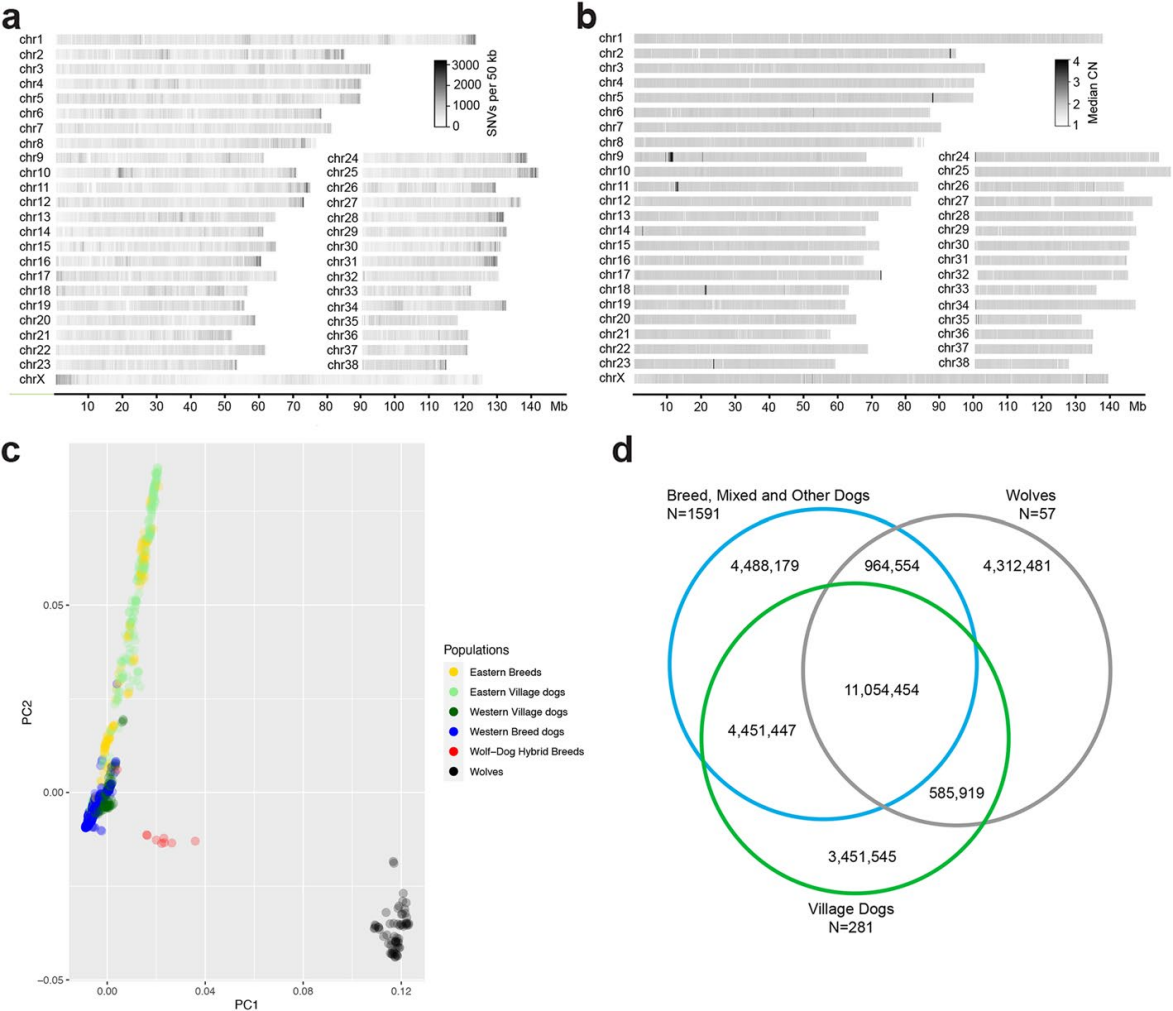
Variant distribution across the genome. **a** SNV density in 50-kb windows, drawn from 1987 samples. Increased SNV density is observed at the X-PAR region and the ends of most autosomes. **b** Median copy number (CN) for 1824 dogs reveals a large, common duplication on chr9 relative to the reference genome. **c** Principal component (PC) analysis separates dog and wolf samples along the first axis while axis two separates dogs from Eastern and Western Eurasia. **d** SNV sharing between the three categories of samples

Our initial variant callset, derived from 1987 dogs, wolves, and coyotes, contains 33,374,690 SNVs across the autosomes and pseudoautosomal region of the X chromosome (X-PAR), and 1,191,860 SNVs on the non-homologous portion of the X chromosome. Using hard filters, we identify a total of 14,414,501 indel and mixed variants. Subsequent analyses are focused on SNVs due to the paucity of validated canine indels available to train refined filters. Based on read depth and mapping quality profiles, we developed a “callable” region annotation and estimated that 96% of the assembly is amenable to short-read variant calling. SNVs show an uneven distribution across chromosomes with a 65% increase in SNV density observed near chromosome ends (*p* < 1 × 10^−30^, Welch’s unequal variances *t*-test) and a moderate correlation with GC content as measured in 50-kb windows (Pearson’s *r* = 0.37) (Fig. 2a).

We subsequently performed a deep analysis of variation in 1929 dog and wolf samples that passed more stringent quality filters (Fig. 1, Additional file 2: Sects. 3 and 9). These samples include 321 breeds, with 261 breeds represented by three or more individuals, 281 village dog samples from 26 different countries, and 57 wolf samples from across Eurasia. Principal component (PC) analysis of SNV genotypes reflects the ancestry of dog and wolf samples (Fig. 2c). The first component accounts for 4.1% of total variation and separates wolves and dogs. PC2 (1.7% of variation) stratifies village dogs and breed dogs based on their origin, with Eastern Eurasian breeds and village dogs at one end of the continuum, and Western Eurasian samples at the other. Samples from the Saarloos Wolfdog and Czechoslovakian Wolfdog breeds, both of which have recent wolf ancestry, show an intermediate placement along PC1. The sole Shiloh Shepherd in the dataset, a breed which may have partial Czechoslovakian Wolfdog ancestry [40], is placed among other Western Eurasian breeds.

### Variation among and within sample groupings

Direct ascertainment of variation from whole genome sequencing permits an assessment of shared genetic variation among breed dogs (including mixed and other breeds), village dogs, and wolves. We first assessed the level of allele sharing among the 1929 analyzed samples (Fig. 2d). The alternative allele was detected in all three sample categories at 37.7% of the 29,308,579 biallelic autosomal SNV sites. Despite making up only 3% of the analyzed samples (57/1929), 14.7% of the variants were present only in sampled wolves, while 15.2% of total sites are absent from wolves. This may be a reflection of the small number of wolves in the study. A total of 11.8% of the variants are found only in village dogs, which represent 14.6% of the samples (281/1929). The combined breed and mixed/other samples represent 82.4% of the total samples (1591/1929), yet only 15.3% of variants are private to this group.

Since rare variants may be informative for inferring recent genetic relationships, we examined variants that were found in only two individuals, i.e., F_2_ sites [41, 42]. We identified 2,384,354 autosomal SNVs which are found in exactly two of the 1929 samples. Most F_2_ sharing was found within groups: 87% of wolf F_2_ sites are shared with another wolf, while 69% of F_2_ sites in breed dogs or village dogs were shared with another breed or village dog, respectively. Reflective of recent shared ancestry, we identified 10 breed dogs who share ≥ 20% of their F_2_ sites with at least one wolf (Additional file 1: Table S2). As expected, this includes the Saarloos Wolfdog and Czechoslovakian Wolfdog breeds [43, 44]. The sole Shiloh Shepherd shares 78% of F_2_ sites with wolves; however, we note that D-statistic analyses do not detect significant allele sharing between the Shiloh Shepherd and wolves relative to that observed in German Shepherd Dogs (Additional file 2: Sect. 3).

To guide future sequencing studies, we estimated the fraction of total variation found in the diverse breeds sampled. Using the observed distribution of non-reference allele counts observed in each breed, we estimated the total number of SNVs expected in a hypothetical set of 100 individuals [45], and compared this value to the total already found in the existing Dog10K call set. Not surprisingly, the predicted fraction of discovered variation varies widely among the 261 breeds represented by at least three individuals (mean = 82.8%, range 47.1–98.4%), and is weakly correlated with the number of individuals sampled per breed (Pearson’s *r* = 0.29). For 22 breeds, we determine that > 90% of the total predicted variants have been identified, while for 20 breeds, ≤ 75% of the total variation has been discovered (Additional file 1: Table S3). For instance, we estimate that the five Norwegian Lundehunds sequenced here capture 98.4% of variation that would likely be discovered in 100 individuals from the same breed. This reflects their well-established closed breeding population structure that was derived from 5–6 individuals [46–48]. In contrast, four Czechoslovakian wolfdog samples capture only 47% of the variation that would be captured in a sample of 100 such dogs. These estimates assume that the sampled individuals are representative of the breed as a whole, and so may be biased if there is within-breed population structure. As these calculations do not account for variation shared between breeds, these predictions represent the lower bounds of the total fraction of variation for each breed already captured in the Dog10K collection.

### Breed relationships and haplotype sharing

**Fig. 3:**
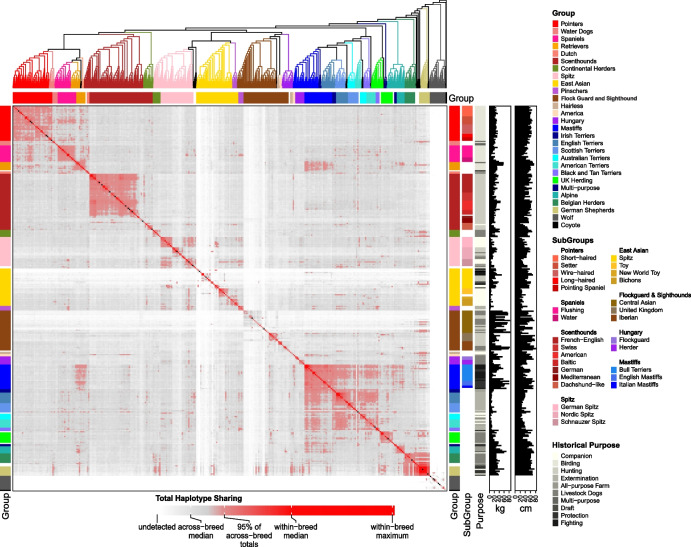
Genetic relationships between 1563 samples spanning 292 breeds. The plot is annotated for major and minor breed groups, breed purpose, and key morphological features. The fraction of haplotype sharing is indicated by the heatmap

We used the Dog10K SNVs to assess the relationships among the sampled breeds. We combined breed subtypes and varieties, resulting in a dataset of 292 breeds represented by more than one dog (Additional file 1: Table S4, Additional file 2: Sect. 3). The output cladogram is based on genomic distance and assessed through 100 resampled data sets. We defined clades as clusters of two or more breeds that share the same branch in > 65% of samplings. We found that 277 of 292 breeds (94.9%) formed monophyletic clusters with 100% confidence, and two additional breeds formed monophyletic clusters with > 90% confidence (Fig. 3). Seven of the 13 breeds that did not comprise a single branch were within the Scenthound clade, where breeds are frequently defined by single morphological features such as color or height.

Overall, the analyzed breeds form 25 major clades comprising two to 49 breeds that cluster in > 65% of permutations (average 90%). Only two breeds, the Norwegian Lundehund and the Löwchen, cluster consistently within clades with which they do not appear to share obvious clade-defining traits. However, the Norwegian Lundehund does not display significant haplotype sharing with any breed, including the Terriers with which it clusters, or with Nordic Spitz types. This is likely related to the drift associated with high levels of homozygosity and random IBS with Terriers. By comparison, the Löwchen shares haplotypes with other small fluffy-type dogs, but clusters with the small hounds and has recent haplotype sharing with the dachshund, suggesting a common origin for the size and coat variation found in these breeds.

The major clades are made up of breeds sharing occupation, morphological traits, and/or geographic origin. Within the larger clades, additional structure can be found with subclades (97% average cluster confidence) displaying a second layer of similarity. In some cases, clade structure reflects the relationships among breed varieties. For example, the German Spitz are split by size, with the Klein and Mittel varieties clustering with the Pomeranian and Volpino Italiano breeds, while the German Giant Spitz and the samples labeled simply as German Spitz clustered with the Keeshond breed. The next most closely related group contains the American Eskimo Dog and Japanese Spitz, two breeds that were created from the German Spitz. Within the scenthound clade, a clade described by occupation, subclades correspond to the geographical origin of the breeds. Alternatively, in the Hungary clade, a clade defined by geographical origin, subgroups can be found that indicate the occupation of member breeds.

We next assessed levels of haplotype sharing among breed dogs. Consistent with previous studies [49], the average haplotype sharing of dogs within a breed is > 40 times greater than the average among dogs from different breeds (average across breeds = 23.5 Mb). Dogs representing breeds within the same clade, as identified on the consensus neighbor joining cladogram, share haplotypes at 3.6 times the average observed in breeds from different clades, and sharing is seven-fold higher for breeds within subclades compared to breeds in distinct clades [(Mann–Whitney test for all above comparisons is *p* < 2.2 × 10^−16^) (Fig. 3)]. The Asian clade, as well as the Flockguards and Sighthounds clade, have the lowest amount of sharing with other clades.

We observe excess haplotype sharing among the terrier clades as well as between the terriers and the Mastiff clades. This is reflective of breed development via admixture or recent ancestry involving multiple clades, where the extent of haplotype sharing correlates with the method of breed development. For instance, there is a long-standing history of terrier and mastiff-type breeds being crossed in the mid-1800s to form multiple bull terrier- and terrier-like breeds such as the Staffordshire Bull Terrier and the Boston Terrier (see the Bull Terrier subclade). There is also excessive sharing between the Mastiff clade and the Retriever clade that has not been observed in previous phylogenies, but suggests recent admixture between these breeds or their ancestors. German Shepherd Dogs and related breeds show the largest number of admixture events with independent breeds from multiple clades (Fig. 3). German Shepherd Dogs, specifically, have sharing values greater than 95% of background levels with 29 breeds from 13 clades and three of the non-clade breeds. Breeds within the German Shepherd clade are the only ones showing significant levels of haplotype sharing with wolves. Since a similar analysis with SNV genotyping arrays and the CanFam 3.1 Boxer reference genome revealed the same result [49], using a German Shepherd Dog reference genome is unlikely to contribute significantly to this observation.

### Runs of homozygosity within sample categories

**Fig. 4:**
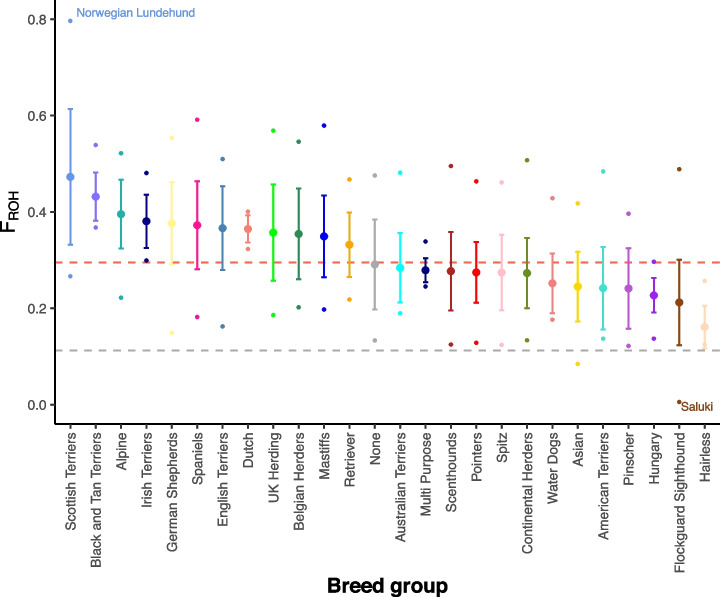
Proportion of the genome covered by ROH (F_ROH_). Mean and standard deviation are plotted for breed groups and are colored as per Fig. 3. Red dashed line shows mean F_ROH_ for breed dogs; gray dashed line shows mean F_ROH_ for wolves. Breeds containing individuals with the highest and lowest F_ROH_ are labeled

Runs of homozygosity (ROH) in an individual’s genome result from the inheritance of two copies of an ancestral haplotype in that individual, and so ROHs are autozygous (homozygous by descent). The estimated proportion of a genome(s) that is in ROH gives a measure of individual or population level inbreeding. For all dog breeds, selection has involved some level of inbreeding and this has resulted in a wide range in ROH across breeds [50–52]. For each genome in the Dog10K collection, we estimated the proportion in ROH (F_ROH_) (Additional file 1: Table S5). This provides high-resolution estimates of historical levels of inbreeding within breeds and breed groups, as well as the genomic coordinates of regions where ROH are never found. Regions lacking ROH may indicate locations where heterozygosity is maintained for correct function. As expected, wild canids show the lowest genome proportions in ROH, with coyotes possessing the smallest total average ROH length (45.2 Mb), and breed dogs having the largest (665 Mb, Table 1). However, there is large variation in these averages, with some individuals and breeds showing particularly elevated, and others particularly low, ROH (Fig. 4, Table 1). For example, a Norwegian Lundehund had the largest number of ROH bases (total ROH = 1842 Mb; F_ROH_ = 78.8%), while a Saluki (sighthound) had the fewest (total ROH = 12.8 Mb; F_ROH_ = 0.56%).

**Table 1:** Runs of homozygosity (ROH) by sample category

	Average ROH		Total ROH	
Category	Count	Length (Mb)	Largest (Mb; genome %)	Smallest (Mb; genome %)
Breed dogs	1267	0.525	1842; 79.6% (Norwegian Lundehund)	12.8; 0.6% (Saluki)
Village dogs	670	0.373	872; 37.7% (Nepal)	8.3; 0.4% (China)
Wolves	570	0.438	946; 40.9% (Sweden)	20.2; 0.9% (Tajikistan)
Coyotes	152	0.298	61.2; 2.6%	38.8; 1.7%

The sample or breed population with the largest or smallest total amount of ROH is indicated

We identified 389 genomic regions that were devoid of ROH in any sample. The ROH-free regions have a mean length of 64.5 kb and range in size from nine bp to a 1.3-Mb region at the start of chr35. The telomeres of all 38 autosomes lack any ROH. We compared all 389 ROH-absent regions to regions of the genome with low depth and mapping quality (“uncallable” regions). We found that 369 of the 389 overlapped for at least 80% of their length with uncallable regions, and all but two overlapped with uncallable regions along at least 20% of their length. The two regions with ROH outside of uncallable regions are gene free, short stretches (1.4 and 0.9 kb) at the ends of chrs22 and 30, respectively. The Dog10K dataset therefore provides sufficient resolution to show the presence of ROH across almost the entirety of the dog genome. A previous study [52] identified a set of 27 genes where at least one exon did not overlap ROH in any of 4342 dogs analyzed by SNV genotyping arrays. Exons of all 27 of these genes were found to be present in at least one ROH in our dataset, suggesting that the lack of ROH in the previous analysis was likely due to the lower-density data derived from genotyping arrays rather than the presence of recessive lethal variants.

### Imputation

**Fig. 5:**
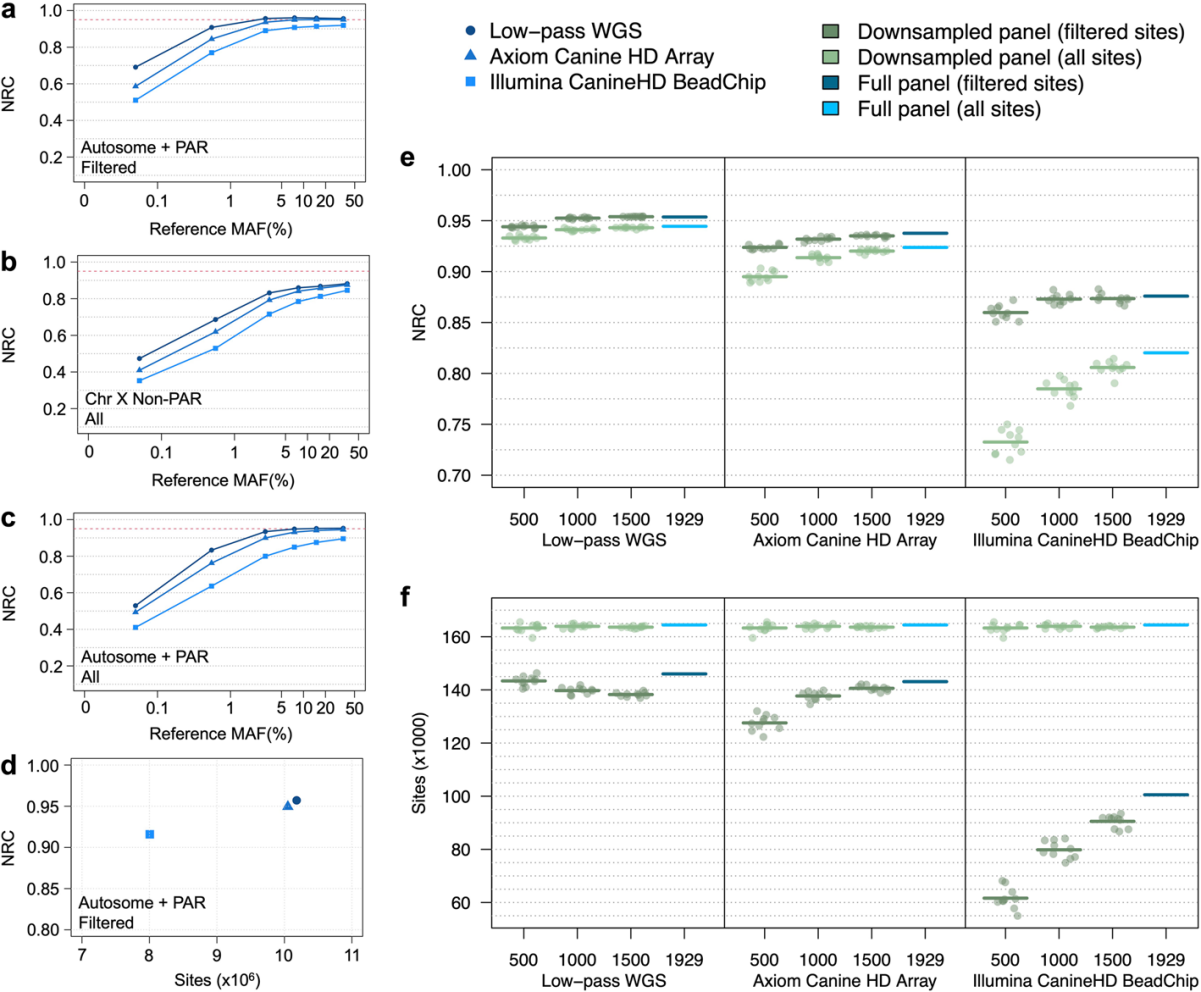
Genotype imputation accuracy of the Dog10K reference panel. **a** NRC rates of imputed genotypes across autosomes and the PAR segment of chromosome X. Variant sites are filtered according to GLIMPSE and IMPUTE5 imputation quality scores (INFO > 0.9). **b** NRC rates of imputed genotypes across the non-PAR segment of chromosome X. Variants are not filtered by imputation quality score, as imputation software does not provide scores for haploid genotypes. **c** NRC rates of imputed genotypes across autosomes and the PAR segment of chromosome X prior to filtering on imputation quality. **d** NRC rates and total number of imputed sites for each platform. Sites were filtered according to imputation quality score > 0.9 and reference MAF > 1%. **e** NRC rates for downsampled and full chromosome 38 reference panels for sites with reference MAF > 1%. Results show both quality and non-quality filtered sites. Data points show NRC rates for a single downsampled reference panel. Horizontal bars indicate mean NRC rates for each reference panel population size. **f** Number of imputed variants for downsampled and full chromosome 38 reference panels for sites with reference MAF > 1%. Results show both quality and non-quality filtered sites. Data points show the number of imputed variants for a single downsampled reference panel. Horizontal bars indicate the mean number of variants for each reference panel population size

The size and breed diversity within the Dog10K dataset provide an excellent opportunity for genotype imputation. The Dog10K imputation reference panel includes all 1929 samples phased for biallelic SNVs with a missing genotype rate < 5%. To test imputation utility, we analyzed 10 publicly available WGS samples representing 10 breeds; five of these breeds were included in the Dog10k collection (Additional file 1: Table S6). Data from each WGS sample were downsampled to represent three separate genotyping platforms; (i) low-pass WGS, (ii) Axiom Canine HD Array, and (iii) Illumina CanineHD BeadChip. Imputation accuracy was positively correlated with platform variant density. For example, imputation based on autosomal and X-PAR sites from low-pass WGS data achieved non-reference concordance (NRC) rates of 0.95 using a reference MAF > 1%. Accuracy rates were maintained for genotypes imputed from the Axiom Canine HD Array sites, but only at a higher reference MAF (> 5%) (Fig. 5a). In contrast, the X chromosome non-PAR had lower imputation accuracy for all three platforms (NRC rates < 0.90, Fig. 5b). Requiring an INFO score > 0.9 improved NRC rates across all platforms, with the largest gain noted for rare alleles (reference MAF < 1%) (Fig. 5a,c). Accuracy rates were similar between the majority of individuals, regardless of whether the imputed individual’s breed was represented in the Dog10K reference panel or not (Additional file 2: Fig. S1).

We next tested the impact of the Dog10K imputation reference panel size on imputation quality and genotype ascertainment. Here, chr38 genotypes from the publicly accessed samples were assessed. From the full Dog10K panel, ten reference panels were created for each of 500, 1000, or 1500 randomly selected individuals. Independent of the modeled genotype platform, the larger reference panels show increased imputation accuracy, although the gains in NRC rates were reduced using panel sizes > 1000 (Fig. 5e). Specifically, NRC rates differed by only 0.001 between the 1000 and 1929 sample panels. Compared to the low-pass WGS platform, NRC rates for the Axiom Canine HD Array and Illumina CanineHD BeadChip array differed by 0.006 and 0.003, respectively. Despite small gains in NRC rates, the larger reference panels revealed increased counts of imputed variants with high-quality scores. For example, the transition from 1000 to 1929 samples resulted in the ascertainment of 6268 chr38 variants for the low-pass WGS platform, 5394 for the Axiom Canine HD Array platform and 20,707 for the Illumina CanineHD BeadChip platform (Fig. 5f).

### Mitochondrial sequence analysis

**Fig. 6:**
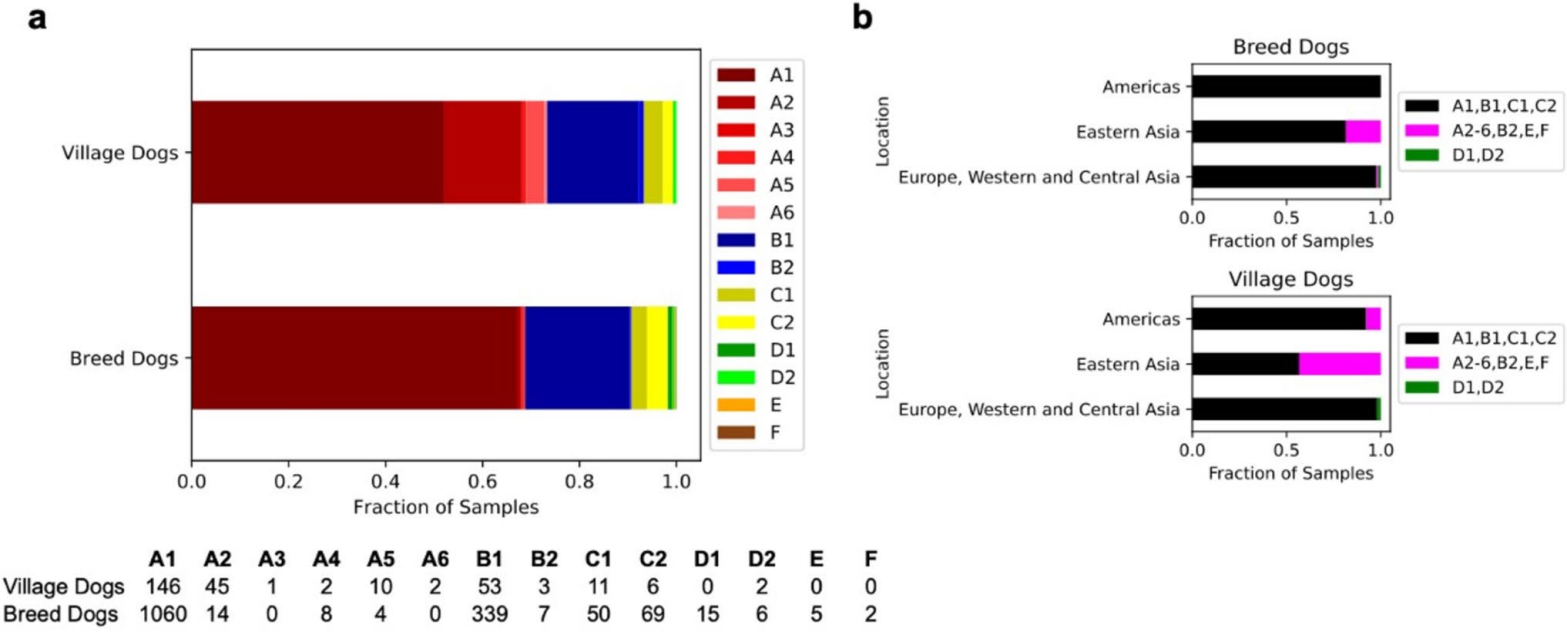
Assignment and distribution of haplogroups in breed and village dogs. **a** Each breed and village dog was assigned to an existing mitochondrial haplotype. **b** Broad geographic categories based on sample origin

The mitochondrial genome is often overlooked in large nuclear genome sequencing projects, despite the importance of mitochondrial variation for forensics [53–56] and studies of ancient and modern canine diversity [10, 57–60]. Here, we reconstruct the mitochondrial genome of 1933 samples, including 1929 dogs and wolves, and four coyotes (Additional file 2: Sect. 6). Consistent with previous expectations [61], most dog mitochondrial genomes (85.8% of dogs) belong to the A1 or B1 haplogroup. Other subclades of A and B as well as clades C, D, E, and F are represented at lower frequencies (Fig. 6a, Additional file 1: Table S1). The most common haplogroups (A1, B1, C1, and C2) have a broad geographic distribution. In contrast, rarer haplogroups such as A2, A3, A4, A5, A6, B2, E, and F are found primarily in Eastern Asia (Fig. 6b).

Across the 1933 individuals, only 887 unique mitochondrial sequences (haplotypes) were observed. The most common was present in 52 individuals (2.69% frequency), and the 12 most common haplotypes were observed in 20% of samples (393/1933 individuals). We calculated the average number of pairwise differences for each group containing at least three samples (six wolf and 18 village dog populations, based on country of origin, and 261 breeds). On average, village dogs contain the highest level of mitochondrial diversity, but a range of variability is seen across groupings. Remarkably, 23 of the 261 breeds with at least three individuals contain only a single mitochondrial haplotype. Linking the mitochondrial and nuclear genomes, we observe a weak correlation between within-breed mitochondrial and autosomal sequence diversity (Spearman correlation of 0.29, *p* = 1.7 × 10^−6^). This correlation is reduced when breeds with no mitochondrial diversity are omitted (Spearman correlation of 0.19, *p* = 0.004).

### Structural variation

Structural variation is an important source of genome variation, and it plays a variety of roles in genome evolution, adaptation, and gene expression [62, 63]. We assessed structural variation (> 50 bp) in a reduced set of 1879 samples with uniform read depth profiles. We constructed paralog-specific copy-number maps based on uniquely mapping 30-mers, and observed notable regions of increased copy number (Fig. 2b), including previously described duplications on chrs9 (*SOX9*) and 18 (*MAGI2*) (Additional file 2: Sect. 7). The UU_Cfam_GSD_1.0 reference, similar to other dogs, lacks the *MAGI2* duplication inserted within the *SOX9* locus. However, both these regions are polymorphic across dogs, contributing to the increased copy number patterns and genome assembly errors [30]. The multi-locus copy number pattern was also evident in wolves (Additional file 2: Fig. S2).

We also noted a 32-kb locus with an extremely large copy-number range located at chr26:31,435,296–31,467,885. The region is duplicated in the UU_Cfam_GSD_1.0 assembly and is highly polymorphic in the Dog10K collection, with QuicK-mer2 [64] copy number estimates of 60–70 copies in dogs, and up to 120 copies for wolves. The region lacks annotated genes and is not found in the human reference genome (hg38). We intersect QuicK-mer2 copy number estimates with coordinates of 18,162 protein-coding genes and observe only 22 genes with a median copy number > 3, including the expected *AMY2B* locus [65] (Additional file 1: Table S7). In total, 1745 protein-coding genes have a copy number range > 2.5 across the Dog10K collection; of these, 546 genes have a single sample that has an outlier estimated copy number.

**Fig. 7:**
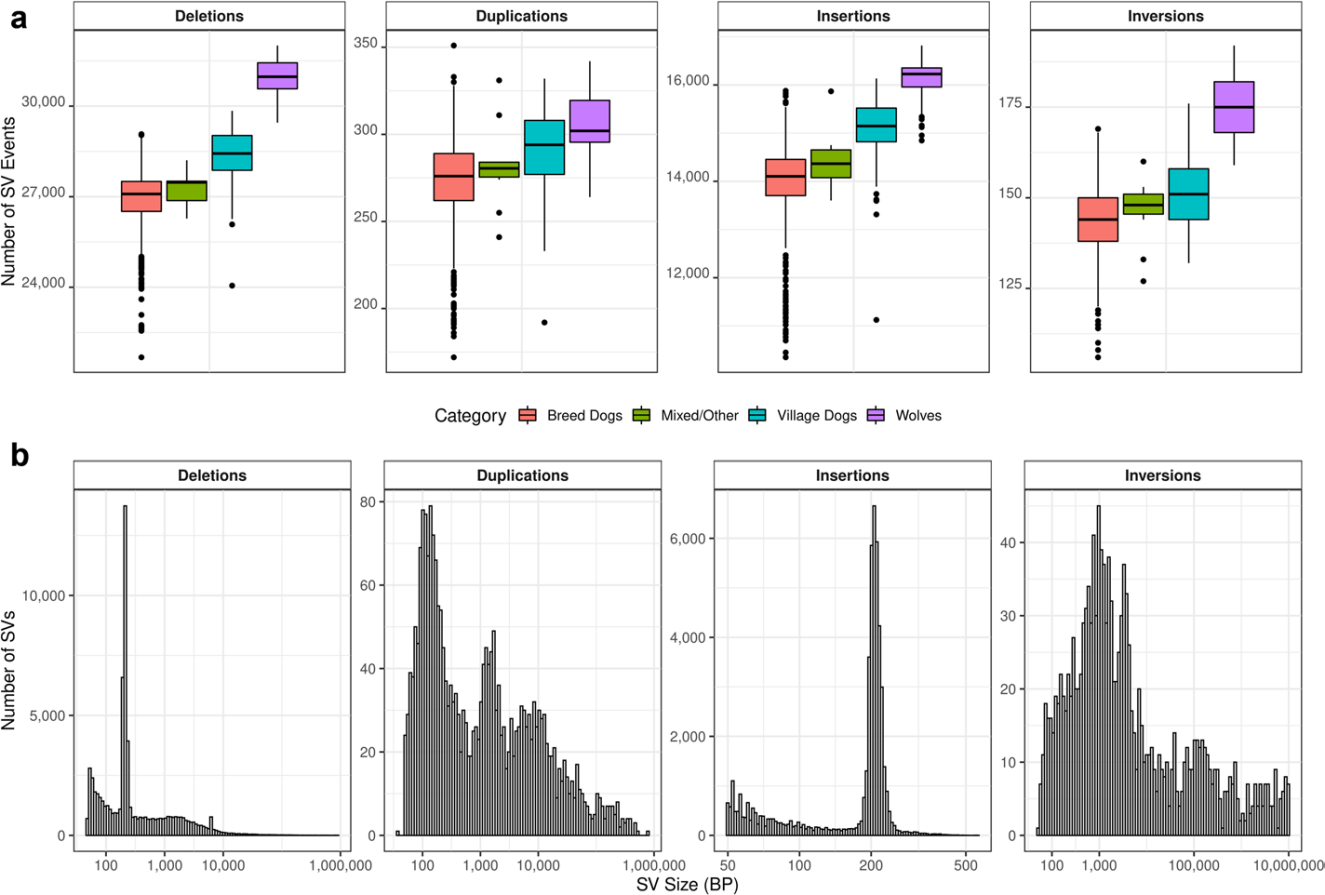
Structural variation detected across 1879 samples. **a** Boxplots of the number of deletion, duplication, insertion, and inversion variants are shown broken down by sample category. **b** Histograms of the size distribution of each class of detected structural variants are shown. An increase in variant count at the ~ 200 bp size bin is apparent for deletions and insertions. This corresponds to the size of SINEC elements

Using Manta [66], which utilizes read-pair and split-read signatures to identify variation, and GraphTyper2 [67], which genotypes structural variants using pangenome graphs, we quantified 147,113 deletion, tandem duplication, insertion, and inversion structural variants (Fig. 7). We assessed linkage disequilibrium (LD) between genotyped structural variants and SNVs and found that 64.7% of deletions, 58.6% of insertions, and 43.8% of duplications are in strong LD (*r*^2^ > 0.8) with a flanking SNV. The lower levels of LD found with duplications likely reflect both a higher mutational recurrence rate and lower genotype accuracy for this SV type. On average, we find 26,960 deletions (affecting a total of 69,950,356 bp) and 14,034 insertions (affecting a total of 2,566,573 bp) in each purebred dog. We detect an average of 14% more structural variants in wolves than breed dogs, including 30,943 deletions (affecting a total of 66,291,676 bp) and 16,071 insertions (affecting a total of 2,761,848 bp) per sample.

Insertion and deletion variants were further queried for intersection with genes. Due to the length range of the deletions, some structural variants impacted multiple genomic feature types (e.g., intron and exon, splice region and untranslated region. Additional file 2: Sect. 7). A total of 31,950 deletions were identified that intersected 12,522 genes, including 5372 genes with an exon deleted. This includes deletion variants identified by Manta that perfectly correspond to the coordinates of introns; additional examination revealed that many Manta deletion calls correspond to the presence of a retrogene. This includes the *FGF4* locus, where both the retrogene variation associated with the chondrodysplasia and chondrodystrophy phenotypes are present in multiple dog breeds [68–70], as well as a 133-kb multi-gene duplication responsible for the dorsal hair ridge in Rhodesian and Thai Ridgebacks [71]. The 133 kb duplication is present in all Rhodesian and Thai Ridgebacks in the Dog10K collection, as well as three African village dogs (Congo, VILLCG000006; Kenya, VILLKE000001; Liberia, VILLLR000017).

We searched the Manta deletion calls for the intron-deletion signature indicative of retrogenes and identified 926 parent genes that have candidate retrogenes (Additional file 1: Table S8). Strikingly, 464 candidate retrogenes were not identified in a recently completed survey of retrogenes in 293 canids [72]. Additional retrogene examples include *G3BP1*, found in 50.4% of breed dogs and only 1.8% of wolves, and *MCMBP*, found in 62.6% of breed dogs and 1.8% of wolves. Both genes were previously identified as having retrogenes in dogs [72, 73].

Retrogene formation utilizes the reverse transcription activity encoded by LINE-1 transposable elements [74, 75]. A LINE-1 encoded protein is also required to mobilize SINEs [76], including the carnivore-specific SINEC elements that make a large contribution to genome differences among canines [29, 77]. The contribution of SINEC elements to canine genomic diversity is apparent as a visible spike in insertion and deletion variant counts ~ 200 bp in size (Fig. 7). RepeatMasker analysis indicates that SINEC sequence represents 31.7% of the deletion and 52.7% of the insertion variants identified (Additional file 2: Fig. S3). Of the 51,950 insertions, 701 intersect with an annotated exon. This includes a 223-bp insertion at chr15:18,164,073, in the second of two exons in *RNASE1* (NM_001313784.1), which is present in 47% of wolves and 0.06% of breed dogs, as well as a 214-bp insertion at chr1:108,879,297 in the third of eight exons of *ELSPBP1* (NM_001002931.1) (found in 47% of breed dogs and 11% of wolves). RepeatMasker analysis indicates that both insertion sequences are SINEC_Cf elements.

### Signatures of selection across breed ancestries

**Fig. 8:**
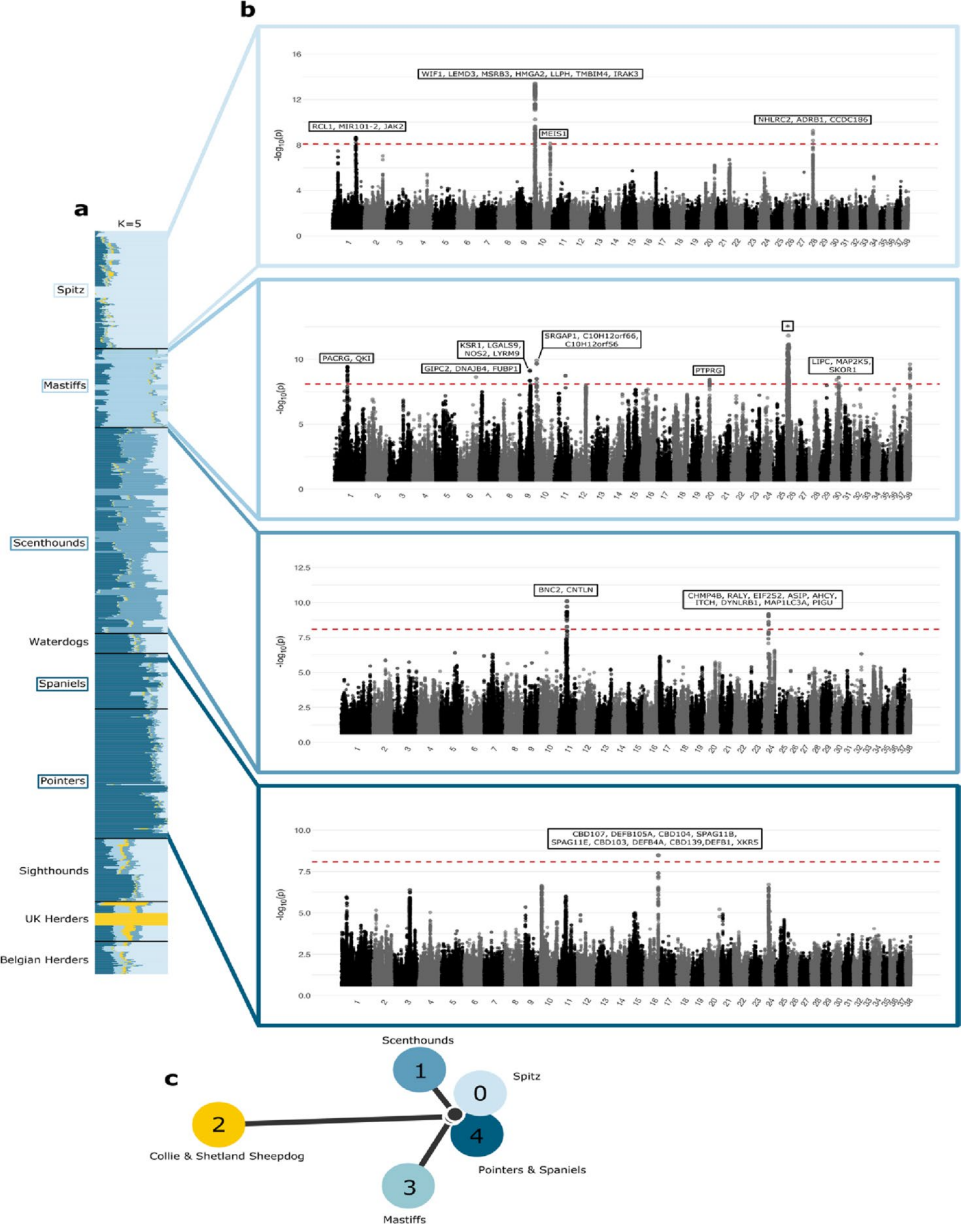
Signatures of selection inferred with ancestry components. **a** Population structure inferred from Ohana for the nine selected dog groups using *K* = 5. **b** Manhattan plots for four of the five ancestral components. Top to bottom: Spitz, Mastiffs, Scenthounds, Pointers, and Spaniels. The red dotted line represents the Bonferroni cutoff and genes either overlapping or within 100 kb from a significant site are indicated at each peak. The asterisk within the Manhattan plot for the Mastiff component contains 88 candidate genes listed in Table S7. **c** Population tree connecting the ancestral components with colors corresponding to the ancestries are shown in the admixture plot. Each ancestral component is labeled based on the dog group(s) for which it is maximized

To test for signatures of selection among major breed clades, we assigned 790 breed dogs into nine groups (Spitz, Sighthounds, Waterdogs, Scenthounds, Pointers, Belgian Herders, UK Herders, Spaniels, and Mastiffs) based on genetic similarity and morphological features (Fig. 3, Additional file 2: Sect. 8). To balance the risks of overfitting with the interpretability of results, we focus on analysis of *K* = 5 ancestral components. These five components are distributed across the analyzed breed dogs and are maximized in the Spitz, Mastiffs, Scenthounds, Pointers, and Spaniels, and a subset of the UK Herders (Collies and Shetland Sheepdogs) (Fig. 8). Using Ohana [78], we then searched for signals of selection in each ancestral component by identifying variants with population differentiation that is not consistent with the genome-wide estimated allele frequency covariance matrix. We set significance levels based on the number of tests performed and considered genes either overlapping or within 100 kb of the significant sites as potential candidates for selection, resulting in 15 loci (Fig. 8, Additional file 1: Table S9).

Several of the candidate regions contain genes associated with variation in size, morphology, and coloration. A region on chr24 that shows selection in the ancestral component maximized in the Scenthounds contains *ASIP*, a major contributor to coat color variation [79–81]. A region on chr16 that shows selection in the ancestral component maximized for Pointers and Spaniels contains multiple beta-defensin and sperm-associated antigen genes (e.g., nine genes across *CBD*, *DEFB*, *SPAG* families). Beta-defensins have been previously linked to coat color; notably, *CBD103* (the K locus) which is located in this region, contains variants associated with black or brindle coat color [82, 83], and may be under selection in wolf populations for resistance to canine distemper virus [84]. The region on chr26 that shows selection in the ancestral component maximized in the Mastiffs is under selection in boxers (five boxers are included in the Mastiff group) [85], contains genes involved in skeletal and muscular development and function and tissue morphology, and is associated with canine body size and height [4].

We found four regions with signals of selection in the ancestral component that is maximized in the Spitz (Fig. 8). The region on chr1 includes *RCL1*, which has been associated with snout ratio and tail curl [86] as well as *JAK2*, which contributes to human [87] and canine primary polycythemia [88]. The strong peak on chr10 includes genes previously linked to ear morphology (*WIF1*, *LEMD3*, *MSRB3*, *LLPH*, and *IRAK3*) [4, 13, 86, 89], body size in humans and dogs [13, 89–91] and beak size in Darwin’s finches (*HMGA2*) [92]. We applied iSAFE [93], a method which ranks candidate favored mutations during a selective sweep based on haplotype and allele frequency patterns, to disentangle the signature in the chr1 locus. Setting Spitz dogs as cases and the remaining samples as controls, we found that the sites with the highest iSAFE scores, including several sites identified by Ohana, cluster in *HMGA2.* Application of iSAFE to other loci revealed broad patterns of high-scoring variants that do not pinpoint a single gene (Additional file 1: Table S10, Additional file 2: Sect. 8).

### Function inference from Dog10K variation

Since variation predicted to alter gene function is expected to be enriched for false positives [94], we applied additional depth and genotype quality filters to the biallelic SNVs identified in 1929 dogs and wolves. These filters removed 0.7% of total available VQSR PASS sites, resulting in 27,878,361 autosomal and 847,128 chrX SNVs utilized for functional analysis (Additional file 2: Sect. 9). On autosomes, 78.9% of filtered sites had an observed MAF > 1%, but the allelic profile for removed sites on chrX differed, with 58.5% of sites observed with a MAF > 1% (Additional file 2: Sect. 9). Both the VQSR PASS and strict-filtered biallelic SNV sets had concordance rates ≥ 99.6%, based on the genotypes of 168 individuals also typed on the Illumina Canine HD Array (Additional file 2: Sect. 10). The strict-filtered SNV set used for functional analyses captures 1.27% of the theoretically possible chrX and autosomal variation (Additional file 1: Table S11), with one SNV every 80 bp when all 1929 individuals were considered.

**Fig. 9:**
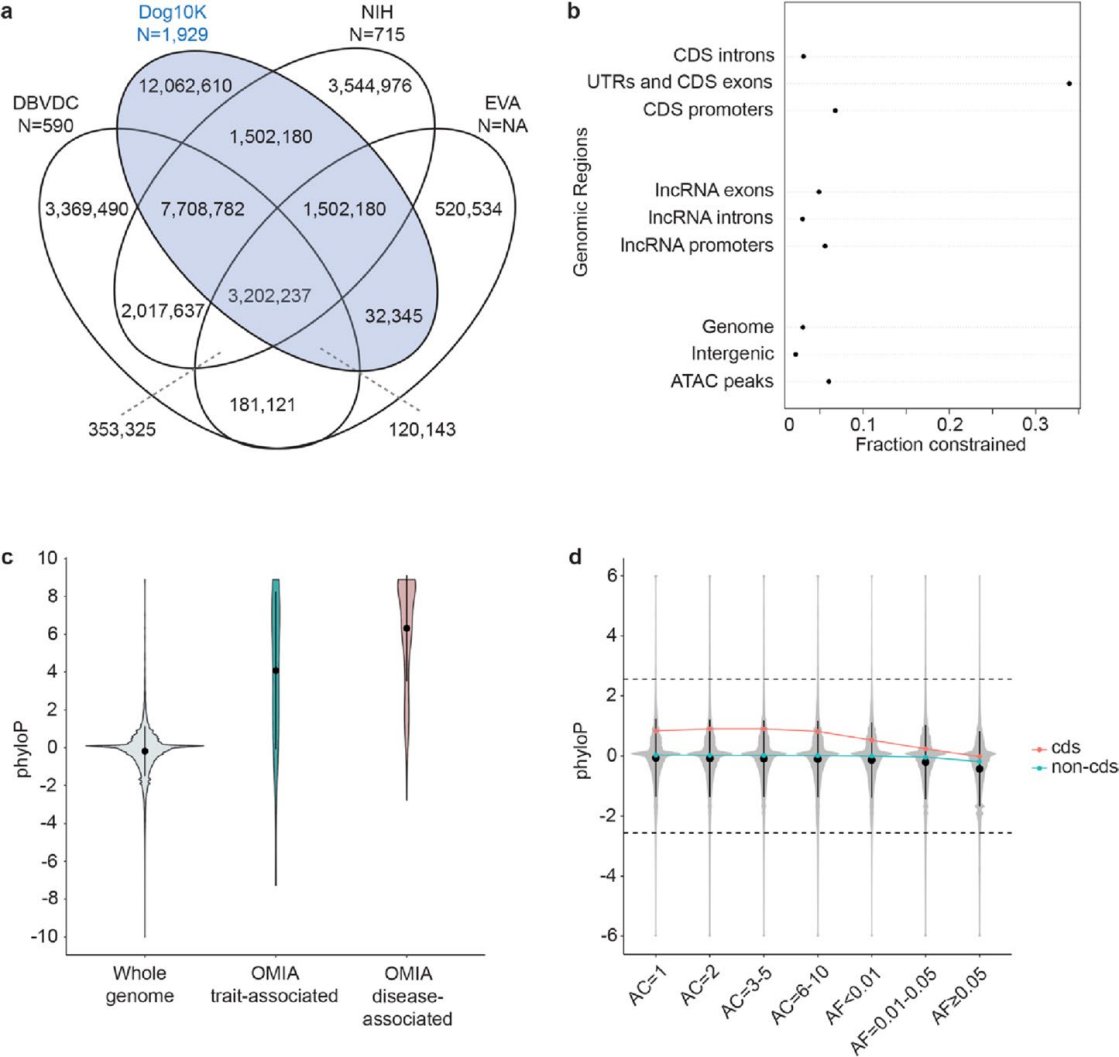
Comparison among variant data sets. **a** Number of variable positions shared between major databases. **b** Fraction of genome spaces under constraint (5% FDR, phyloP > 2.56). **c** Enrichment of constrained bases in OMIA trait (blue), and disease (red) sets compared to the genome as a whole (gray). **d** Relationship between allele count (AC), allele frequency (AF), and phyloP score for the coding (CDS, red) and non-coding (non-CDS, green) bases in the whole genome (gray)

Panels of normal variation are key to prioritizing SNVs for downstream functional analyses. We compared the composition and biallelic SNV sites contributed from only dogs and wolves (when known) for three such panels, DBVDC (590 samples, 20,443,472 SNVs) [6], NIH panel (715 samples, 18,468,060 SNVs) [4], and the European Variation Archive (EVA) RS Release 3. Additional file 1: Table S12 summarizes the sample acquisition and distinct alignment and site filtering strategies for each panel. These factors, including minimum coverage depth (ranging from 2 × to 10 ×), impact the number of samples and variants available for downstream analyses (Additional file 2: Sect. 11). We find that 43% of SNVs are unique to the Dog10K collection (Fig. 9) and that 98% of these unique variants are rare (AF < 1%) and are not due to differences between the CanFam3.1 [2] and UU_Cfam_GSD_1.0 [30] assemblies. This variation is in part a reflection of the diversity and uniqueness of the dog breeds included in Dog10K (60% of breeds are only found in the Dog10K collection, Additional file 1: Table S13), as well as the limited sample sharing between this and the other sets (only 10/1929 samples were shared; Additional file 2: Sect. 11). As expected, given gene density, recombination rates and other demographic pressures, genetic variation within the Dog10K dataset was not evenly spread across the genome, with example outlier peaks observed on chrs12 and 18 which harbor the dog leukocyte antigen (DLA) and olfactory receptor genes, respectively (Additional file 2: Fig. S4).

### Base-pair constraint for functional prioritization

One of the goals of the Dog10K consortium is to provide the community with a set of SNVs that can be used to aid in the identification of phenotypic associations. Within the coding region of the genome, we identify 7607 high and 129,766 moderately deleterious SNVs within the 1591 breed dog dataset (67% with AF < 1%; Additional file 1: Tables S14 and S15). Across the entire genome, we used estimates of evolutionary constraint to infer function, with Zoonomia single base-pair phyloP scores calculated from an alignment of 240 mammalian species [95]. Here, CanFam3.1 referenced phyloP scores were converted to UU_Cfam_GSD_1.0 coordinates, revealing that 3.5% of the genome is under constraint (purifying selection; 5% FDR, phyloP ≥ 2.56). A large fraction of constraint bases is observed in protein-coding genes (CDS and UTRs), but an appreciable 2.2% of intergenic space is also constrained (Fig. 9b).

To benchmark the utility of the Zoonomia phyloP scores, we examined the distribution of positions classified as disease-associated or other trait-associated in the curated Online Mendelian Inheritance in Animals (OMIA) database [96]. The median phyloP score for both sets was greater than the 5% FDR for constraint, indicating that the associated bases are enriched for regions of the genome under selection (Fig. 9c). Within breed dogs, a negative correlation was observed between allele frequency and phyloP score (Fig. 9d), although as noted from studies in other species, common variants in constrained positions may be involved in local adaptation. In breed dogs under selection, variation at these positions may also result in favorable trait outcomes, such as the *HPS3* g.44487038G > A variant (phyloP = 7.03), associated with the “cocoa” brown color segregating in French Bulldogs [97].

### OMIA variants found in the Dog10K collection

Breed dogs were submitted to Dog10K with the expectation that they were free from known disease. However, they cannot be evaluated for phenotypes that develop late in adulthood, nor can health be ascertained for village dogs or wolf samples. We therefore examine the frequency distribution of trait-associated (morphology or other) and disease-associated variants accessed from OMIA [96]. For this analysis, 337 SNVs and small indels, each variant included in the OMIA database, and each spanning less than 20 consecutive bases, were selected for interrogation. Of these, 76 variants were detected in at least one individual in the Dog10K collection (Additional file 1: Table S16). As expected, the alternative allele frequency of the morphology-associated variants spanned all frequency classes (14 variants, Alt AF = 0.2–72%), and for each, all three genotype classes were observed (Additional file 1: Table S17). Overall, 58 OMIA disease-associated variant positions were detected in Dog10K, with 62 homozygous occurrences detected across 15 disease traits (Table 2, Additional file 1: Table S16). This was not unexpected for some diseases where affected individuals have variants associated with a mild phenotype, sex-limited inheritance, late-onset, or incomplete penetrance.

**Table 2:** Dog10K samples with likely causal homozygous genotypes for autosomal recessive diseases, risk factors, or traits

Trait	OMIA ID	Gene	Homozygous samples (N)	Dog10K breeds or village dogs carriers
Lens luxation	000588–9615	*ADAMTS17*	1	American Toy Terrier
Persistent Mullerian duct syndrome	000791–9615	*AMHR2*	1	Miniature Schnauzer
Laryngeal paralysis and polyneuropathy	002301–9615	*CNTNAP1*	1	Pyrenean Shepherd
Exercise-induced collapse	001466–9615	*DNM1*	2	Curly Coated Retriever
Dwarfism, growth-hormone deficiency	001473–9615	*GH1*	6	Bolonka, Brussel Griffon, Petit Brabancon Griffon
Lundehund syndrome	002031–9615	*P3H2*	4	Norwegian Lundehund
Ichthyosis, PNPLA1-related	001588–9615	*PNPLA1*	2	Golden Retriever
Progressive rod-cone degeneration	001298–9615	*PRCD*	4	Australian Cattle Dog, Entlebucher Mountain Dog, Portuguese Podengo, Swedish White Elkhound
Hypotrichosis, recessive	001279–9615	*SGK3*	4	American Hairless Terrier
Urolithiasis	001033–9615	*SLC2A9*	6	Dalmatian, Majorca Mastiff
Oculocutaneous albinism, type IV	001821–9615	*SLC45A2*	1	Bullmastiff
Degenerative myelopathy (risk factor)	000263–9615	*SOD1*	22	many (incl. village dogs)
Thrombocytopenia, TUBB1-related	002434–9615	*TUBB1*	2	Norfolk Terrier
Von Willebrand disease I	001057–9615	*VWF*	2	Kromfohrländer
Von Willebrand disease II	001339–9615	*VWF*	4	Boykin Spaniel, German Spitz

### HWE deviation to identify candidate disease variants

Since individuals within the Dog10K collection were assumed to be healthy at the time of sampling, we hypothesize that disease variants would show depleted homozygous frequencies [98]. Using the VQSR PASS SNV VCF as an input, we find 42 missense variants that pass the initial filtering criteria, with genotype and variant site quality statistics further narrowing the candidate list to seven SNVs. After visual inspection of alignment files, it was evident that in each case, other factors such as the existence of pseudogenes, assembly artifacts, and structural variation provided more parsimonious explanations for observed departures from HWE. We note that 13/42 HWE deviation candidates are retained within the strict-filtered VCF (Additional file 1: Table S18).

### Variants affecting metabolism and drug targets

**Fig. 10:**
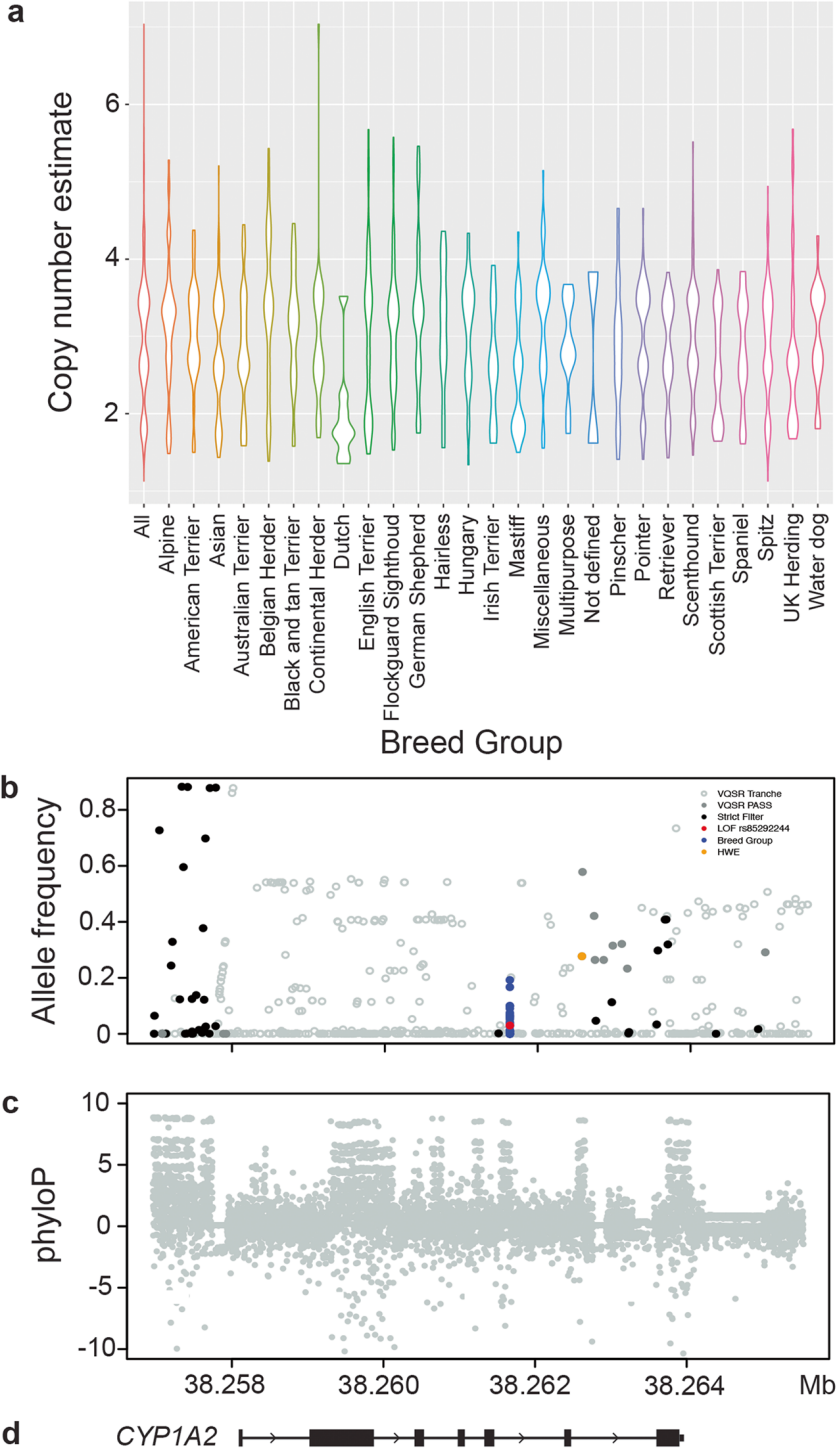
Structural and point variants impacting *CYP1A2*. **a** Distribution of copy number variation within all breed dogs, and the major clades. **b** Location of SNVs across *CYP1A2* inclusive of filtering or impact status. **c** Mammalian phyloP scores (bounded by 10, − 10). **d** Illustration of the region from the reference genome perspective

To illustrate the utility of the Dog10K collection, we searched for genetic variation affecting druggable targets. From the 1427 genes in the human Tier 1 druggable gene set [99], we identified 79 genes with their full coding sequence impacted by SVs, and 249 genes with high-impact snpEff SNV annotations (375 SNVs, median phyloP = 3.16). At the known SV variable selective phosphodiesterase type 4 inhibitor, *CYP1A2* (28), 49.7% of samples (934/1,879) are estimated to have a copy number ≥ 3. This variability is noted in all major breed clades (Fig. 10a). This locus provides the opportunity to visualize the impact of SNV filtration and functional consequence (Fig. 10b). The allele frequency of SNVs failing the VQSR PASS tranche (open gray circles), passing this tranche (filled gray circles), and available after strict filters (black circles) are plotted. Highlighted in red is the loss of function SNV, rs852922442, for which the C > T causes a premature stop codon and decreased *CYP1A2* expression [100, 101]. While rare across dog breeds overall (AF = 0.03), the rs852922442-T allele is common in individuals from the German Shepherd and Scenthound clades (AF = 0.19 and 0.07, 26 and 202 samples respectively), while notably absent from the Mastiff, American Terriers, Australian Terriers and Belgian Herder clades (96, 31, 27, and 26 samples, respectively; Fig. 10b, blue dots). This wide AF distribution likely explains the observed interindividual variability associated with the pharmacokinetics of *CYP1A2*-substrate drugs in dogs. This locus also includes an HWE deviation candidate variant (orange circle), discounted due the presence of the locus spanning SV.

**Fig. 11:**
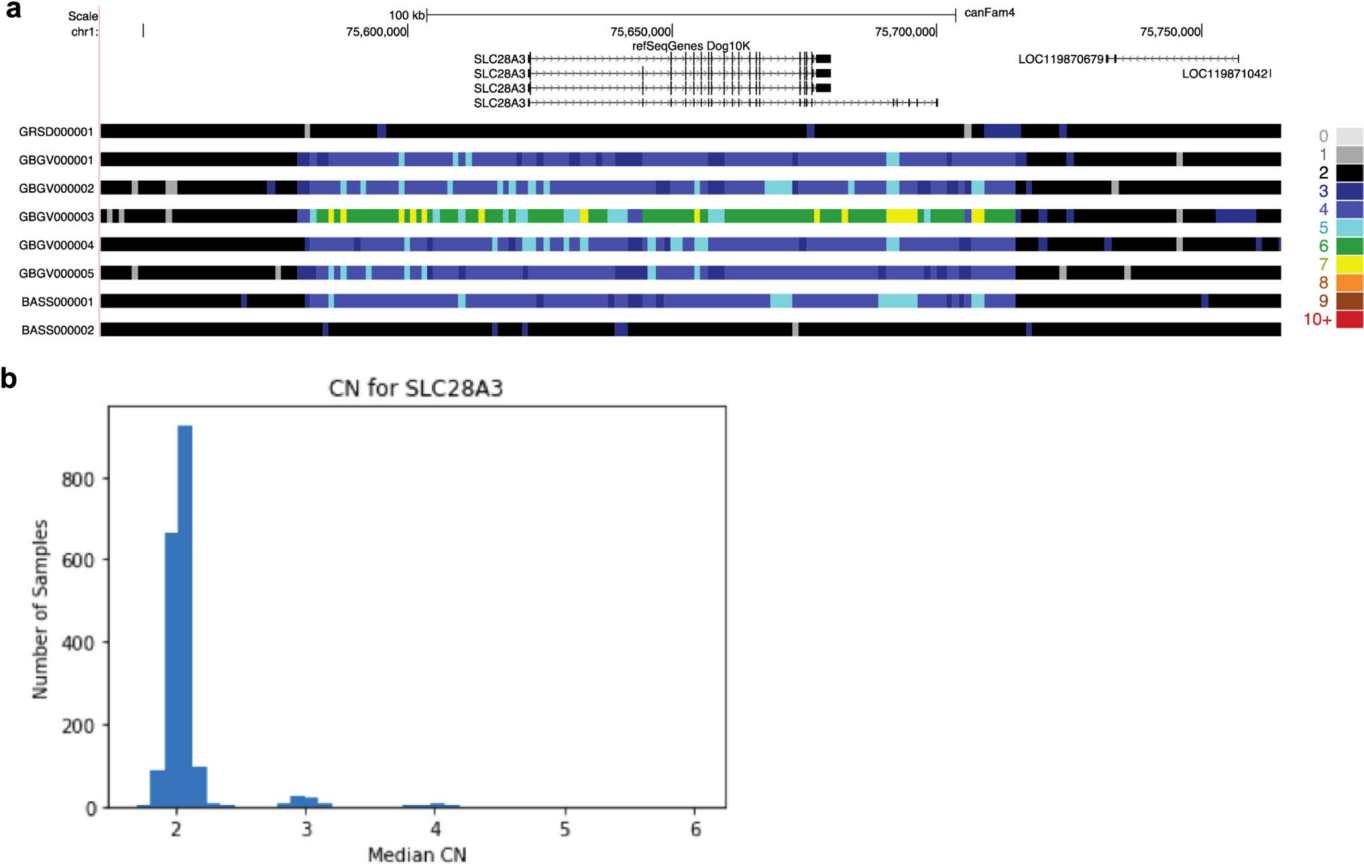
*SLC28A3* locus copy number expansion. **a** Location and span of the copy number element in polymorphic Grand Basset Griffon Vendéen (GBGV) and basset hound (BASS) individuals contrasted with copy number two samples from the German Shepherd Dog (GRSD) and BASS breeds. **b** Distribution of the median copy number count across the 1879 samples assayed

Another interesting druggable target is a previously unknown SV, which spans the entire coding sequence of *SLC28A3* (Fig. 11a). Across breeds, we observe clearly defined profiles corresponding to gene copy numbers of two, three, and four (Fig. 11b). All Grand Basset Griffon Vendéen dogs (GBGV) have a copy number of ≥ 4 at this locus, with one individual (GBGV000003) inferred to have a copy number of six (Fig. 11a). SLC28A3 (previously CNT3) is a concentrative nucleoside transporter with many functions. While no high-impact coding variants are present in the Dog10K strict variant catalog, coding variation in the human *SLC28A3* ortholog are known to influence the metabolism of gemcitabine [102, 103], a drug used to treat solid tumors in human [104] and canine patients [105, 106]. Outside of the clinic, one of the most interesting observations has been the *SLC28A3* association with advanced maternal age in studies of the methylome [107]. At least one study, which profiled the DNA methylomes of paired parental peripheral blood and cord bloods from nuclear families, revealed that methylome-associated expression changes in many genes, including *SLC28A3*, are related to adverse outcomes in advanced maternal age pregnancy, a serious consideration in canines.

## Discussion

The Dog10K consortium sequenced and analyzed a large and diverse genomic sampling of canids. Our results encompass a harmonized resource of more than 48 million SNVs, indels, structural variants, and mitochondrial sequences as well as a set of pipelines and best practices for expansion to larger data sets. The identified variants are a valuable dataset that will enable future studies into the demographic and selective history of canids and serve as a panel of variation useful for the exploration of diseases and other phenotypes.

Our refined dataset includes 1579 samples from 321 breeds or defined populations, of which 261 are represented by three or more dogs (Additional file 2: Sect. 3). In addition, we sequenced 293 mixed breed and village dogs, and 57 wolves sampled from multiple geographic regions which, in aggregate, allows us to capture not only considerable levels of phenotypic diversity but permits the ascertainment of substantial levels of genetic variation. Our comprehensive ROH analysis is likely to prove key to understanding the historical relationships among modern breeds, the history of breed development, and the relationships between modern, historical, and ancient canids. With one variant every 80 bp, the Dog10K collection has captured most of the genetic variation present in 22 common breeds. This allows future efforts to focus on other rare breeds or geographically isolated populations to reveal the role of undiscovered variation in canine biology and evolution.

Our variant filtering pipeline leveraged sites routinely genotyped in commercial arrays as a training set to identify 34.5 million high-quality SNVs. Since a robust truth set is not available for indel variants, we applied hard filters based on criteria recommended by the GATK best practices to identify 14.4 million indels. The indel total includes sites with a mixture of SNV and indel alleles. Our indel to SNV ratio of 2.4 is similar to that reported by two other recent surveys of dog and wolf variation [6, 30]. However, another study of canines, which included additional outgroup samples from the *Canis*, *Cuon*, and *Lycalopex* genera, reports an SNV to indel ratio of 4.2 [4], while studies of equines [108], bovines [109], and humans [110] report SNV to indel ratios greater than 10. It is not clear to what degree the apparent excess of indel variation in canines reflects true biological differences or technical artifacts in calling. Given this uncertainty, our analysis is primarily focused on SNVs.

Our analyses demonstrate the utility of the Dog10K variant dataset as a reference panel for use in genotype imputation [111], an approach which has been shown to be effective in making use of low-pass or poor-quality sequence data [112, 113]. Canine studies have successfully incorporated this approach [114–117], particularly for disease GWAS, leading to identification of a risk haplotype for congenital laryngeal paralysis in Alaska sled dogs [118], and a locus for canine idiopathic pulmonary fibrosis in West Highland white terriers [114], among others.

The largest previous study, based on a panel of 676 dogs from 91 breeds with 97 high-coverage WGS dog samples downsampled to approximately 1 × coverage per sample, demonstrates that both quality filtering and MAF were critical to accuracy [117]. Both affect power to conduct successful GWAS, with a previous study demonstrating that as the MAF difference between cases and controls is reduced, the number of samples required for imputation of low-pass WGS to reach the same power in a GWAS as high-coverage WGS grows exponentially [117]. While this study suggested discarding sites with a MAF < 0.05, our data argues for selecting variants with imputation quality > 0.90 and reference MAFs > 1%. This reflects both the large number of dogs and breeds as well as the variation captured in village dogs in our dataset, both of which are critical for the development of any reference panel, in dogs [119, 120] or otherwise [121]. For the Illumina CanineHD BeadChip platform, the criteria we propose will provide imputed genotypes with NRC rates > 0.85 for over 8 M sites, whereas for the low-pass WGS and Axiom Canine HD Array platforms, these criteria provide NRC rates of approximately 0.95 for over 10 M sites (Fig. 5d). It is important to note, however, that any imputation analysis is only as accurate as the samples in the reference panel, and expansion to a panel even larger than we present here, is an important long-term goal.

By modeling allele frequency changes and admixture, we identified regions that show frequency differentiation across five ancestral components found throughout the analyzed breeds. Analysis reveals that many of these signals likely reflect selection for body size and coat color during the establishment of breeds, several of which were identified previously, thus validating the completeness of the Dog10K dataset. The precise identification of the genes targeted by selection during breed formation is hindered by the extended range of linkage disequilibrium in breed dogs [122]. As a result, identified loci often contain multiple genes previously associated with disparate phenotypes. For example, a large region on chr26 shows signatures of selection in the ancestral component that is maximized in the Mastiff group. This 7.5-Mb region shows an extended reduction in nucleotide diversity relative to other clades (Additional file 2: Fig. S5), and includes genes associated with canine body size and height [4] as well as glioma risk [123] and other cancer phenotypes [124–126].

To refine the selection candidates, we applied iSAFE [93], a method for fine mapping mutations favored during selective sweeps, to the regions we identified. Our analysis nominates *HMGA2*, a known regulator of canine body size, as the likely target in the chr10 locus that was selected in the ancestral component that is maximized in Spitz dogs. However, for the remaining loci, we observe a broad pattern of high-scoring candidate variants distributed throughout the candidate region. Further dissection of such loci will require combinations of selection scans, association studies with well measured phenotypes, preferably including samples from multiple breeds, and functional follow-up.

By combining tools for the discovery and genotyping of structural variants, we present a genome-wide catalog of insertion, deletion, duplication, and inversion variants. Association of these variants can now be assessed in ongoing genome-wide studies. Examination of the size spectrum of the detected variants highlights the disproportionate contribution of LINE-1 encoded proteins to canine genome diversity. Although the variant discovery approach we used is unable to resolve large insertions associated with LINE-1 sequences, we found that 31.7% of deletions and 52.7% of discovered insertions are SINEC sequences that are variably present among the samples in the Dog10K collection. Unexpectedly, many of the deletion variants we identified reflect the presence of retrogenes. These insertions are missing from the UU_Cfam_GSD_1.0 reference and are derived from 926 parent genes. Since our retrogene discovery was limited to deletions corresponding to the full length of introns, a targeted discovery approach is likely to identify additional retrogenes present in the Dog10K collection, reinforcing retrogenes as an important class of canine genetic variation [72].

Rigorous quality controls and filters are essential to identify rare variants that have a functional impact. The Dog10K collection utilizes a common sequencing source, as well as harmonized alignment and variant calling pipelines that aim to reduce the impact of batch effects on variant quality. We note however, from comparisons with OMIA and analysis of Hardy–Weinberg Equilibrium, that even our most strictly filtered callset is not free of false positives. These errors are multicausal and illustrate the challenges encountered in analysis of large-scale sequencing studies or diverse breeds. For example, the genotype of a female village dog from Azerbaijan was heterozygous for the chrX variant, NSDHL:c.700G > A (p.Gly234Arg). The same genotype has been reported in a Chihuahua with verrucous epidermal keratinocytic nevi [127], a disease with X-chromosomal semi-dominant inheritance and presumed embryonic lethality in hemizygous males (OMIA002117-9615). Inspection of the short-read alignments revealed that the village dog heterozygous variant call was a false-positive, a technical artifact caused by the insertion of an *NSDHL* retrogene with a c.[700G > A] allele on chr14.

This example further highlights the challenges that retrogene insertions pose for canine clinical genetics [17, 128]. Additionally, our variant set includes a site in *CYP1A2* that is out of Hardy–Weinberg equilibrium (Fig. 10). This position is targeted on existing SNV genotyping arrays and was included in the VQSR truth training set, and thus survived the resulting variant filters. In both the *NSDHL* and *CYP1A2* examples, access to alignment files and additional SNV quality metrics allowed the errors to be identified. Despite advances in variant filtration methodologies, manual curation of rare, functionally important variants remains essential.

The scale of the Dog10K variant collection makes this a valuable resource for functional prioritization. Again using the OMIA analyses, we find 179 samples homozygous for a *BTBD17* variant associated with a 46,XX disorder of sex development and embryonic lethality [129]. This non-coding single-base insertion (XM_038546704.1:c.85 + 206_85 + 207insG) was observed at a frequency of 22%, which is higher than expected for a variant that causes a severe disease (Additional file 1: Table S17). While the original finding of homozygous lethality was reported only in German Shorthaired Pointers, this is a timely reminder that putative disease-associated variants should be carefully investigated prior to use in non-discovery breeds or populations, where the association between variant and pathogenic effect has yet to be confirmed.

We also examined the potential of the Dog10K collection to aid in the translation of pharmacogenetics. Here, the collection could point towards breed groups fixed for LOF variants, or highlight groups that need additional care in veterinary prognostic treatment. The *CYP1A2* locus has known clinical significance, as it plays a rate limiting step in the metabolism of multiple veterinary drugs including theophylline, clozapine, and tacrine [130]. We find that the *CYP1A2* locus is copy number variable across all defined breed groups (Fig. 10a), suggesting that this expansion predates breed formation. We also see breed group variability at the *CYP1A2* loss of function allele, rs852922442-T (Fig. 10b). Here, both sampled Keeshonds were homozygous LOF, providing a spontaneous canine model to study the compensatory effects of this gene knockout [131, 132]. While the role of *CYP1A2* is well-established, the roles of other potential drug targets examined in this study remain to be elucidated and require cautionary comment. Genes such as *SLC28A3* play roles in many biological processes spanning nutrient metabolism to COVID-19 therapy pharmacogenomics [103, 133], and we cannot assume a phenotypic outcome from gain or LOF variants. It is important to establish an a priori hypothesis, collect large numbers of samples, and phenotype each sample meticulously before appling genome level observations to clinical decisions.

## Conclusions

Variants identified in the Dog10K collection represent a global view of canine genome diversity that informs functional interpretation and enables future studies. The mapping and processing pipelines of Dog10K are open to the community, allowing for the expansion of additional samples to capture and exploit the full extent of canine diversity.

## Methods

### Sample selection, sequencing and read alignment

DNA was isolated from 2075 canids, comprising 1649 breed dogs, 336 village dogs, 18 dogs of mixed origin or that are not recognized by any international registering body (labeled as “mixed/other”), 68 wolves, and four coyotes supplied by investigators from eight sites (Additional file 1: Table S1). Samples were collected as per each institution’s animal care, collection, and use protocols (Additional file 1: Table S19). Whole genome sequencing was carried out using the Illumina HiSeq X Ten platform by Novogene (Inc.) in Tianjin, China. Approximately 0.2 µg of DNA from each sample was sheared into ~ 350 bp with the Covaris system, and sample index libraries generated using the NEB Next® Ultra™ DNA Library Prep Kit for Illumina (NEB, USA) following the manufacturer’s recommendations.

To analyze the nuclear genome, raw sequencing reads were aligned to a modified version of the Wang et al. German Shepherd Dog genome assembly [30] (UU_Cfam_GSD_1.0, GCF_011100685.1), supplemented by three Y chromosome sequences from a Labrador Retriever (ROS_Cfam_1.0, GCF_014441545.1) assembly. Read data was processed across multiple centers using a shared GATK-based pipeline prior to centralized genotyping and filtration of candidate variants on the autosomes and chrX. Following alignment, samples were removed due to low coverage (< 10 ×), the presence of sample duplicates, mislabeled or unknown breed identity, or potential contamination indicated by reference read fraction at heterozygous positions. To identify candidate SNVs, we applied the Variant Quality Score Recalibration (VQSR) procedure with cutoffs that retain 99% of variants present on the Illumina CanineHD BeadChip and Axiom K9 HD genotyping arrays. The alignment pipeline is described in more detail in Additional file 2: Sect. 1.

Different analyses require different levels of variant stringency and sample membership. These are summarized in Fig. 1 and in Additional file 2: Sects. 2 and 9. For genome-wide assessment of SNV density, the VQSR tranche 99 primary PASS SNV (VQSR PASS) call set was used. This is derived from 1987 samples (1593 breed dogs, 309 village dogs, 18 mixed/other, 63 wolves, and 4 coyotes). Most analyses utilize a set of 1929 samples (1579 breed dogs, 281 village dogs, 12 mixed/other, 57 wolves) that pass more stringent quality controls. For *SNV functional analyses*, additional filters were applied to the VQSR PASS SNV set. These included filters based on depth (–minDP 5), genotype quality (–minGQ 20), and an in-house allelic balance (0.70 ≥ AB ≤ 0.30) filter based on the vcf4.2 allele depth (AD) INFO field. Parameters were adjusted to suit autosomes or chrX. Filtering was followed by iterative steps of variant and sample missingness. Mitochondrial analysis utilized the set of 1929 samples supplemented by the additional inclusion of four coyotes (Additional file 2: Sect. 6). For *structural variant studies*, 1879 samples with uniform depth profiles were utilized. SVs detected using Manta v1.6.0 [66] and genotyped using GraphTyper2 v2.7.2 [67]. Genome-wide copy-number estimates were created using QuicK-mer2 [64]. Detailed methods are included in Additional file 2: Sect. 7.

### Reference annotation

The reference genome was annotated with the reference appropriate NCBI gene annotation files (https://ftp.ncbi.nlm.nih.gov/genomes/all/annotation_releases/9615/106/). All gene, transcript, exon, and CDS annotation attribute fields were updated following annotation conventions used by Ensembl. When duplicate copies of the gene ID annotation were observed, version numbers were modified in accordance with annotation copy number within the UU_Cfam_GSD_1.0 assembly, rather than annotation copy number across all assemblies annotated under NCBI release 106. This process identified 118 duplicate gene IDs, including 33 with protein-coding biotype (Additional file 1: Table S20, Additional file 2: Sect. 12). Using liftover, the reference genome was further annotated with Zoonomia CanFam3.1 phyloP scores (accessed March 2022) and regions of open chromatin (BarkBase ATAC annotation [3]).

### Identification of regions amenable to SNV calling with short sequencing reads

A genome callability mask was created to facilitate downstream analyses. Positions marked (i) “N” in the genome reference, (ii) where ≥ 10% of aligned reads have a mapping quality (MQ) of 0, or (iii) where the total coverage was more than 50% away from the median coverage were identified as “unmappable regions”. Separate cutoffs were determined for the autosomes and X-PAR region, and the non-PAR segment of the X chromosome.

### SNV variation within and between groups

To interrogate recent shared ancestry, we measure the statistic F_2_ in 1929 samples. F_2_ notes variants found in only two samples regardless of their zygosity and is similar to the count of f_2_ variants (or doubletons), i.e., those present exactly twice in a sample [41, 42]. We utilized F_2_ rather than f_2_ due to the wide range of inbreeding found across individuals. We utilized 2,384,354 autosomal F_2_ sites that have no missing genotypes. We predicted the total number of variants present in each breed that has at least three individuals based on the distribution of non-reference allele counts (the non-reference site frequency spectrum). Based on this distribution, we predicted the number of non-reference variants that would be discovered in a sample of 100 individuals of the same breed by applying a linear program method to the observed site frequency spectrum [45].

### Breed genetic distance and haplotype sharing

Autosomal variation from the VQSR PASS set (26,486,238 SNVs) sourced from 1579 breed dogs, 57 wolves, and four coyotes were used as inputs. Genomic distance (1-IBS) was calculated in PLINK (v1.9) [134]. The distance matrix was transformed into a cladogram using *neighbor* in the PHYLIP suite of programs [135] and visualized with FigTree (v1.44, http://tree.bio.ed.ac.uk/ software/figtree). To determine the significance of branch placement in the cladogram, the dataset was resampled 100 times by pulling a random 10% of the SNVs to make 100 distance matrices. The cladograms created from each of the random variant-set matrices were combined using *consense* in the PHYLIP suite of programs. Clades were defined as clusters of two or more breeds that share the same branch in > 65% of samplings. We additionally made a comparison dataset by first randomly identifying one dog from each breed then removing SNVs with a linkage disequilibrium value of *r*^2^ > 0.5 within a 500-kb window leaving 3,106,329 SNVs. Bootstrapped distance matrices were created by randomly drawing 3.1 million SNVs from this dataset with replacement 100 times. Cladograms were created with *neighbor* and combined using *consense* (part of PHYLIP). The placement of samples relative to each other was assessed and individuals were removed from the breed analysis if they (i) did not cluster with the multi-breed clade that contained all other members of the same breed; (ii) they were listed as an ambiguous breed; or (iii) they were part of a population that included first-generation hybrids. Shared haplotypes of at least 250 kb were estimated using BEAGLE v4.1 [136] and haplotypes with a LOD > 3.0 were predicted to be identical-by-descent. The length of all shared segments was totaled for every pair of dogs and these totals were averaged within each breed, within each clade, and across clades. D-stats were calculated for German Shepherd-like breeds to assess wolf admixture. The R package *admixr* [137] was used to run Admixtools v7.0.2 [138] on the tree structure (W, X)(Y, Z) where W = German Shepherd Dog, Z = Coyote, X = list of German Shepherd-related breeds, and Y = list of wolf populations. Significance was set at |*Z*|≥ 3. Additional detail is provided in the Additional file 2: Sect. 3.

### Runs of homozygosity

Runs of homozygosity (ROH) for all samples were defined using the sliding-window approach implemented in PLINK v1.90b4.9 [134] with the “--homozyg” function. Settings were based on those previously recommended for high-density SNP datasets [139], with minimum average SNP density (--homozyg-density 50), maximum gap between adjacent SNPs (--homozyg-gap 1000), the size of the sliding window (--homozyg-kb 200), and the minimum number of variants needed to detect ROH (--homozyg-window-snp 100) set to reflect the average SNP density of the dataset. The “--homozyg-window-het” and “–homozyg-window-missing” flags were set to 3 and 2 respectively to account for potential sequencing errors and missing data. The number of heterozygous sites to allow within a window (--homozyg-window-het 3) was set based on the average number of heterozygous sites called in male dogs outside of the pseudoautosomal regions on chrX (i.e., where all males are haploid and therefore any heterozygous calls are errors). The coefficient of inbreeding was calculated from our ROH estimates (F_ROH_) by dividing the total length of all ROH within a sample by the genome size (i.e., F_ROH_ is the proportion of the genome within ROH). The BEDTools v2.29.2 [140] *subtract* function was used to identify all regions in the genome that are absent of ROH across all samples. These were intersected with genome callability mask (BEDTools *intersect*).

### Imputation

The Dog10K reference panel was created using all 1929 samples and the VQSR PASS SNV VCF. Multiallelic and sites with missingness > 5% were removed. SHAPEIT5 was used for phasing [141]. In total, 29,234,830 autosomal and 965,534 chrX SNVs are included. Public WGS were used to assess imputation outputs. The 10 samples are drawn from 5 breeds in, and 5 not in, the Dog10K collection (Additional file 1: Table S6). Each WGS was processed as described above. To represent low-pass WGS, alignment files were downsampled to 1 × coverage. To represent array genotypes, Axiom CanineHD Array sites (530,104 sites) and Illumina CanineHD BeadChip sites (134,037 sites) were first lifted to UU_Cfam_GSD_1.0 (liftover [142]) and subsequently extracted from each WGS. Different methods were required to impute the three downsampled genotype methods. For low-pass WGS data, genotype likelihoods were calculated using bcftools v1.17 mpileup and call commands, followed by GLIMPSE v1.1.1 imputation of genotypes from genotype likelihoods [143]. For Axiom and Illumia array data, genotypes were phased using SHAPEIT5, rare allele MAF cutoff set to 0, and the Dog10K reference panel for reference haplotypes followed by IMPUTE5 imputation of the phased array data [144]. Both genotype imputation tools were run with default parameters. For chrX non-PAR, males and females were imputed separately, with males run using the haploid settings of both imputation tools. Genotype imputation accuracy was measured as the non-reference genotype concordance (NRC) between imputed genotypes and WGS genotypes. NRC rates were assessed for site imputation quality (imputation info metric > 0.9), MAF within the reference panel, and genotype chromosomal context (autosome/PAR or X chromosome). Ten additional chr38 smaller reference panels were created for each of 500, 1000, and 1500 individuals by selecting samples at random from the full sample list of 1929 individuals. Chromosome 38 genotypes from the selected dogs were then extracted from the VQSR PASS VCF, processed, and phased in accordance with the methodology used to create the full Dog10K reference panel.

### Mitochondrial analyses

A modified GATK Mutect2 pipeline [145] was used to call mitochondrial variation. Read-pairs from the nuclear genome alignment process were extracted if (i) at least one read aligned to the UU_Cfam_GSD_1.0 chrM sequence, or (ii) to a nuclear mitochondrial segment that is at least 300 bp long with at least 95% identity to the reference mitochondrial genome sequence. Extracted read-pairs were aligned to two linear references based on the NC_002008.4 mitochondrial genome reference sequence. The first reference was identical to NC_002008.4, the second is rotated to start at position 8000. Using two genome sequences compensates for the bias in the lower rate of alignment for reads derived from the ends of the linear sequence (bwa-MEM [146] v0.7.15). Read depth was calculated (GATK v4.2.5.0 *CollectHsMetrics*) and downsampled to a depth of 5000 (GATK v4.2.5.0 *DownsampleSam*). Mutect2 was used to identify candidate variants from each alignment with options --mitochondria-mode, --max-reads-per-alignment-start 75, --max-mnp-distance 0, and --annotation StrandBiasBySample. The resulting VCF was filtered with GATK FilterMutectCalls --mitochondria-mode. VCF files from both references are then merged, with variants in the first and last 4 kb taken from the alignment to the rotated reference. Sites where the most frequent alternative allele fails the strand_bias filter or represents a heteroplasmy (an allele fraction less than 0.5) were removed. Regions with a coverage less than 100 and regions that overlap positions 15,512–15,535 or 15,990 were masked to “N”. The accuracy of the mitochondrial variation discovery pipeline was assessed by comparing the mitochondrial sequence constructed from Illumina data to that reported in five published long-read canine genome assemblies. Assignment to mitochondrial haplogroups was performed based on similarity to previously defined samples [60]. More detail is provided in the Additional file 2: Sect. 6.

### Structural variation

CNVs were detected with the QuicK-mer2 [64] search command with default parameters (*k* = 30, edit distance = 2, depth-threshold 100). Control regions for copy number and GC normalization were defined by excluding non-autosomal chromosomal sequence, regions that are duplicated in the genome assembly based on assembly self-alignment [147], reported CNVs [30], and regions with an elevated copy number identified in a preliminary analysis using fastCN [148]. Samples with a median absolute copy number estimate deviation greater than 0.25 were excluded from the analysis. The paralog-specific copy number for each gene was estimated based on the median QuicK-mer2 estimate of intersecting windows for each sample. This analysis was limited to the 18,162 protein-coding genes that were fully encompassed by at least one k-mer window. Structural variants were identified with Manta v1.6.0 and default parameters [66]. Inversions were converted to event representation using the Manta convertInversion.py utility. Raw calls were merged using svimmer and genotyped across all samples using GraphTyper2 v2.7.2 with default parameters [67]. For break-end (BND), insertion (INS), deletion (DEL), and duplication (DUP) calls, the “AGGREGATED” genotyping model was used. For inversion (INV) candidates, the breakpoint model was used as reported by GraphTyper2. SVs were filtered for quality, depth, and allelic balance, with a maximum size of 10 Mb. Candidate retrogenes were identified by deletions that have a 99% reciprocal overlap with annotated introns (Additional file 2: Fig. S6). See Additional file 2: Sect. 7 for full details.

### Signatures of selection

Ohana [78] was used to detect signals of selection shared across nine dog breed groups we defined based on allele sharing patterns (See “Breed genetic distance and haplotype sharing”). The 790 individuals within the broader Spitz, Sighthounds, Waterdogs, Scenthounds, Pointers, Belgian Herder, UK Herding, Spaniel, and Mastiffs groups possess similar morphological traits (Additional file 1: Table S21, Additional file 2: Sect. 8). Only biallelic PASS SNVs with a minor allele frequency (MAF) of 5% and no missing data were considered (6,181,086 autosomal sites). Ohana ran with the number of ancestral components ranging from *K* = 2 up to *K* = 11, with *K* = 5 selected as a compromise between low risk of overfitting and interpretability of component identity. The five inferred ancestral components were maximized for the following dog groups: Mastiffs, Scenthounds, Spitz, Pointers and Spaniels, and the Collie and Shetland Sheepdog. The log-likelihood ratio test statistic of Ohana’s *selscan* module was used to evaluate the likelihood of selection for each variant. Genomic control was carried out, and *p*-values were calibrated using a mixed chi-squared distribution with the *emdbook* R package (version 1.3.12) [149]. A 5% Bonferroni threshold for the number of sites analyzed was used as a significance threshold (− log_10_*p* = 8.09). The *intersect* function of BEDTools v2.30.0 [140] was used to identify genes overlapping or within 100 kb of the significant sites.

To refine the targets of selection, we applied iSAFE [93], a method to fine-map variants targeted by selection that does not rely on additional demographic information of the study populations or functional annotation of the mutations under focus. We applied iSAFE to each region, including flanking regions, setting the cases as the clade in which each ancestral component was maximized and using the remaining clades as controls. The loci on chr26 and chr38 were not analyzed due to their large size. Additional details are provided in the Additional file 2: Sect. 8.

### Variant concordance

The quality of our variant collection was assessed by comparing genotypes for 168 sequenced samples that were previously genotyped on the Illumina CanineHD array. Concordance was calculated for both sites retained in the VQSR PASS and strict filter sets (151,197 and 145,271 polymorphic sites respectively, Additional file 2: Sect. 10).

### SNV functional annotation

The annotated VCF catalog (See “Reference annotation”) was further filtered by sample category (Breed Dog And Other *N* = 1591, Village Dog *N* = 281, Wolf *N* = 57) and functional annotation with s*npsift* from snpEFF 4.3t [150]. SNV density was calculated in 100-kb bins for various sample categories, allele frequencies (vcftools 0.1.16 [151]: rare, AF ≤ 1%; Intermediate, 1% < AF < 5%; common, AF ≥ 5%) in both the coding and non-coding fractions of the genome (Additional file 2: Sect. 12).

### Comparison of public variation catalogs

The strict-filtered Dog10K dataset was compared to three other publically available datasets in multiple ways, (i) methods used to call variants within each set, (ii) sharing of individuals between sets, and (iii) sharing of breed types. The sets were strict-filtered Dog10K VCF (1929 samples, 28,725,482 SNVs), DBVDC (590 samples, 20,443,472 SNVs) [6], NIH (715 samples, 18,468,060 SNVs) [4], and EVA v3 (4,548,628 SNVs) (http://ftp.ebi.ac.uk/pub/databases/eva/rs_releases/release_3/by_species/canis_lupus_familiaris/). CanFam3.1 referenced datasets were lifted to UU_Cfam_GSD_1.0 coordinates, with variants on unplaced scaffolds excluded from further analysis. The full NIH panel contains multiple canid outgroups (Additional file 1: Table S12). These were removed, allowing for the comparison of positions variable in dogs and wolves. For (i) the methods and filters used to call variants was tabulated, and due to this variability, for (ii) individuals were considered shared between datasets if their proportion of IBD was in excess of that observed for the closest pair in the Dog10K dataset (i.e., PLINK (v1.9) [134] PiHAT > 0.9451 based on 145,845 random SNVs). For (iii) breed types, breed names and descriptors were harmonized and compared across sets.

### Fraction of theoretical variation discovered

The fraction of possible variants captured by the strict filter set was calculated by first summing the number of each base contained in the callable region of UU_Cfam_GSD_1.0. In this analysis, complementary bases were combined, i.e., C and G, and T and A. Observed base changes were extracted using the strict-filtered VCF with BCFtools *stats* function [152]. Calculations were performed separately for the autosomes and chrX.

### Intersection with OMIA database

Variant information for 463 published likely causative variants for canine inherited traits and diseases were downloaded as a CSV file from OMIA [96] (omia.org; March 2022). The analysis was restricted to SNVs, and small indels spanning less than 20 consecutive nucleotides, leaving 352 “small” variants. Positions were lifted from CanFam3.1 to UU_Cfam_GSD_1.0 using the chain file downloaded from UCSC (https://hgdownload.soe.ucsc.edu/goldenPath/canFam4/liftOver/). The lifted positions were extracted from the functional Dog10K dataset using BCFtools *isec* (samtools version 1.10) [152]. The resulting file was manually curated, genotype distributions were tabulated, and the OMIA traits for the identified variants were annotated.

### Variants affecting metabolism and drug targets

The Tier 1 of 1427 human druggable target genes [99] was downloaded and where matching with a UU_Cfam_GSD_1.0 referenced NCBI annotation (“Reference annotation”) taken forward for analysis. Tier 1 includes gene targets of approved small molecules or biotherapeutic drugs, as well as clinical-phase drug candidates from the time of publication. The Tier 1 gene space was intersected with high effect coding variants from the “SNV functional annotation” and CNVs from the Quick-mer2 [64] “Structural variation” analysis. Only genes completely covered by a Quick-mer2 window were considered. See Additional file 2: Sect. 12.

### SNV deviations from HWE

Using the VQSR PASS VCF as an input, deviations from HWE were determined for each biallelic, missense, or loss of function variant. Calculations were based on the chi-square distribution at a Bonferroni adjusted *p*-value < 0.05 (R v4.2.0). For each of 42 HWE candidate positions, variant site statistics (BaseQRankSum, FS, MQ, MQRankSum, QD, ReadPosRankSum, SOR, and VQSLOD) and genotype statistics (depth, allele depth, and phred-scaled genotype likelihoods) were extracted using the *vcfR* package [153]. Each genotype statistic was analyzed according to its assigned genotype of either reference or alternate. Based on quality scores and potential biological interest, the relevant alignment files of seven variants were selected for additional visual analysis (IGV v2.10.0 [154]). In addition, reads containing the variant of interest were mapped to the ROS_Cfam_1.0, UMICH_Zoey_3.1, UNSW_CanFamBas_1.0, UU_Cfam_GSD_1.0, and Dog10K_Boxer_Tasha long-read assemblies using the NCBI blast tool to identify potential alternative causes of HWE deviation.

### Supplementary Information

**Additional file 1: Table S1.** Sample metadata and analyses where used. **Table S2.** Samples with high F2 allele sharing with wolves. **Table S3.** Variation yet to be discovered based on 100 samples per breed. **Table S4.** Breed group placement of each sample. **Table S5.** Runs of homozygosity (ROH) statistics per sample. **Table S6.** Publicly available test samples used to measure imputation accuracy. **Table S7.** Copy number variable genes. **Table S8.** Potential retrogenes. **Table S9.** Candidate genes either overlapping or within a 100kb distance of a significant site for each targeted ancestry. **Table S10.** iSAFE top 10 sites per selection signature and ancestry component. **Table S11.** Observed fraction of theoretically possible SNVs. **Table S12.** Alignment and filtering strategies for three panels of normal variation. **Table S13.** Breed categories included in three panels of normal variation. **Table S14.** Summary of variant counts in Dog10K sample sets. **Table S15.** Distribution of SNVs across functional classes and Dog10K sample sets. **Table S16.** Genotypes observed for 76 OMIA variants. **Table S17.** Allele frequency distributions detected in Dog10K for OMIA categories. **Table S18.** Variant positions deviating from HWE. **Table S19.** Animal protocols, approving board, and date of approval. **Table S20.** Summary of duplicate genes within NCBI release 106. **Table S21.** Samples used in Ohana selection analysis.

**Additional file 2:** Supplementary Methods [165–182]. **Fig. S1.** Imputation accuracy of individual samples for sites with MAF > 1%. **Fig. S2.** Median copy-number across the genome for wolves. **Fig. S3.** Repeatmasker classification of SINE variation. **Fig. S4.** Distribution of variation across the genome for breed and other dogs (n=1,591). **Fig. S5.** Nucleotide diversity along chr26. **Fig. S6.** Signature of a retrogene detected at the *TEX2* locus.

**Additional file 3.** Review history.

## Data Availability

Raw sequence data is available from the SRA under accessions PRJNA648123 [155] and PRJNA188158 [156] and are listed in Additional file 1: Table S1. SNV and SV variants have been deposited to the European Variation Archive (PRJEB62420) [157] and mitochondrial genomes are available in GenBank (accessions OQ339232-OQ341164). Variant files and associated annotations are available at https://kiddlabshare.med.umich.edu/dog10K/ and at https://zenodo.org/record/8084059 [158]. Code to perform genome alignment, variant calling, and mitochondrial processing is available under the MIT Open Access License at https://github.com/jmkidd/dogmap [159], https://github.com/jmkidd/doggenotype [160], and https://github.com/jmkidd/callmito [161]. Archival versions are available under the MIT Open Access License at https://zenodo.org/record/8087879 [162] https://zenodo.org/record/8087891 [163] and https://zenodo.org/record/8087897 [164].
