# Author Correction: Genome sequencing of 2000 canids by the Dog10K consortium advances the understanding of demography, genome function and architecture

**DOI:** 10.1186/s13059-023-03101-w

**Published:** 2023-11-07

**Authors:** Jennifer R. S. Meadows, Jefrey M. Kidd, Guo-Dong Wang, Heidi G. Parker, Peter Z. Schall, Matteo Bianchi, Matthew J. Christmas, Katia Bougiouri, Reuben M. Buckley, Christophe Hitte, Anthony K. Nguyen, Chao Wang, Vidhya Jagannathan, Julia E. Niskanen, Laurent A. F. Frantz, Meharji Arumilli, Sruthi Hundi, Kerstin Lindblad-Toh, Catarina Ginja, Kadek Karang Agustina, Catherine André, Adam R. Boyko, Brian W. Davis, Michaela Drögemüller, Xin-Yao Feng, Konstantinos Gkagkavouzis, Giorgos Iliopoulos, Alexander C. Harris, Marjo K. Hytönen, Daniela C. Kalthof, Yan-Hu Liu, Petros Lymberakis, Nikolaos Poulakakis, Ana Elisabete Pires, Fernando Racimo, Fabian Ramos-Almodovar, Peter Savolainen, Semina Venetsani, Imke Tammen, Alexandros Triantafyllidis, Bridgett vonHoldt, Robert K. Wayne, Greger Larson, Frank W. Nicholas, Hannes Lohi, Tosso Leeb, Ya-Ping Zhang, Elaine A. Ostrander

**Affiliations:** 1 Science for Life Laboratory, Department of Medical Biochemistry and Microbiology, Uppsala University, 75132 Uppsala, Sweden; 2 Department of Human Genetics, University of Michigan, Ann Arbor, MI 48107 USA; 3 State Key Laboratory of Genetic Resources and Evolution, Kunming Institute of Zoology, Chinese Academy of Sciences, Kunming, 650223 China; 4 National Human Genome Research Institute, National Institutes of Health, 50 South Drive, Building 50 Room 5351, Bethesda, MD 20892 USA; 5 Section for Molecular Ecology and Evolution, Globe Institute, University of Copenhagen, Øster Voldgade 5-7, 1350 Copenhagen, Denmark; 6 University of Rennes, CNRS, Institute Genetics and Development Rennes - UMR6290, 35000 Rennes, France; 7 Institute of Genetics, Vetsuisse Faculty, University of Bern, 3001 Bern, Switzerland; 8 Department of Medical and Clinical Genetics, Department of Veterinary Biosciences, University of Helsinki and Folkhälsan Research Center, 02900 Helsinki, Finland; 9 School of Biological and Behavioural Sciences, Queen Mary University of London, London, E14NS, UK and Palaeogenomics Group, Department of Veterinary Sciences, Ludwig Maximilian University, D-80539 Munich, Germany; 10 Broad Institute of MIT and Harvard, Cambridge, MA 02142 USA; 11 BIOPOLIS-CIBIO-InBIO-Centro de Investigação Em Biodiversidade E Recursos Genéticos - ArchGen Group, Universidade Do Porto, 4485-661 Vairão, Portugal; 12 Department of Public Health, Udayana University, Bali, 80361 Indonesia; 13 Department of Biomedical Sciences, Cornell University, 930 Campus Road, Ithaca, NY 14853 USA; 14 Department of Veterinary Integrative Biosciences, School of Veterinary Medicine and Biomedical Sciences, Texas A&M University, College Station, TX 77843 USA; 15 Department of Genetics, School of Biology, Aristotle University of Thessaloniki, Thessaloniki, Macedonia 54124, Greece and Genomics and Epigenomics Translational Research (GENeTres), Center for Interdisciplinary Research and Innovation (CIRI-AUTH, Balkan Center, Thessaloniki, Greece; 16 NGO “Callisto”, Wildlife and Nature Conservation Society, 54621 Thessaloniki, Greece; 17 Natural History Museum of Crete & Department of Biology, University of Crete, 71202 Irakleio, Greece; 18 Biology Department, School of Sciences and Engineering, University of Crete, Heraklion, Greece; 19 Palaeogenomics and Evolutionary Genetics Lab, Institute of Molecular Biology and Biotechnology (IMBB), Foundation for Research and Technology - Hellas (FORTH), Heraklion, Greece; 20 Department of Gene Technology, Science for Life Laboratory, KTH - Royal Institute of Technology, 17121 Solna, Sweden; 21 Department of Genetics, School of Biology, Aristotle University of Thessaloniki, 54124 Thessaloniki, Macedonia Greece; 22 Sydney School of Veterinary Science, The University of Sydney, Sydney, NSW 2570 Australia; 23 Department of Ecology and Evolutionary Biology, Princeton University, Princeton, NJ 08544 USA; 24 Department of Ecology and Evolutionary Biology, Ecology and Evolutionary Biology, University of California, Los Angeles, CA 90095-7246 USA; 25 Palaeogenomics and Bio-Archaeology Research Network, School of Archaeology, University of Oxford, Oxford, OX1 3TG UK

**Author Correction: Genome Biol 24, 187 (2023)**

**https://doi.org/10.1186/s13059-023-03023-7**

Following publication of the original article [1], the authors reported errors in Fig. 5 and Figure S1. The blue geometric symbols for Illumina Canine HD BeadChip and Axiom Canine HD Array were swapped in the figure keys. The colors of the lines and symbols in the plots are correct. The corrected Fig. 5 is given below. The updated additional file 2 is published in this correction article.

**Fig. 5:**
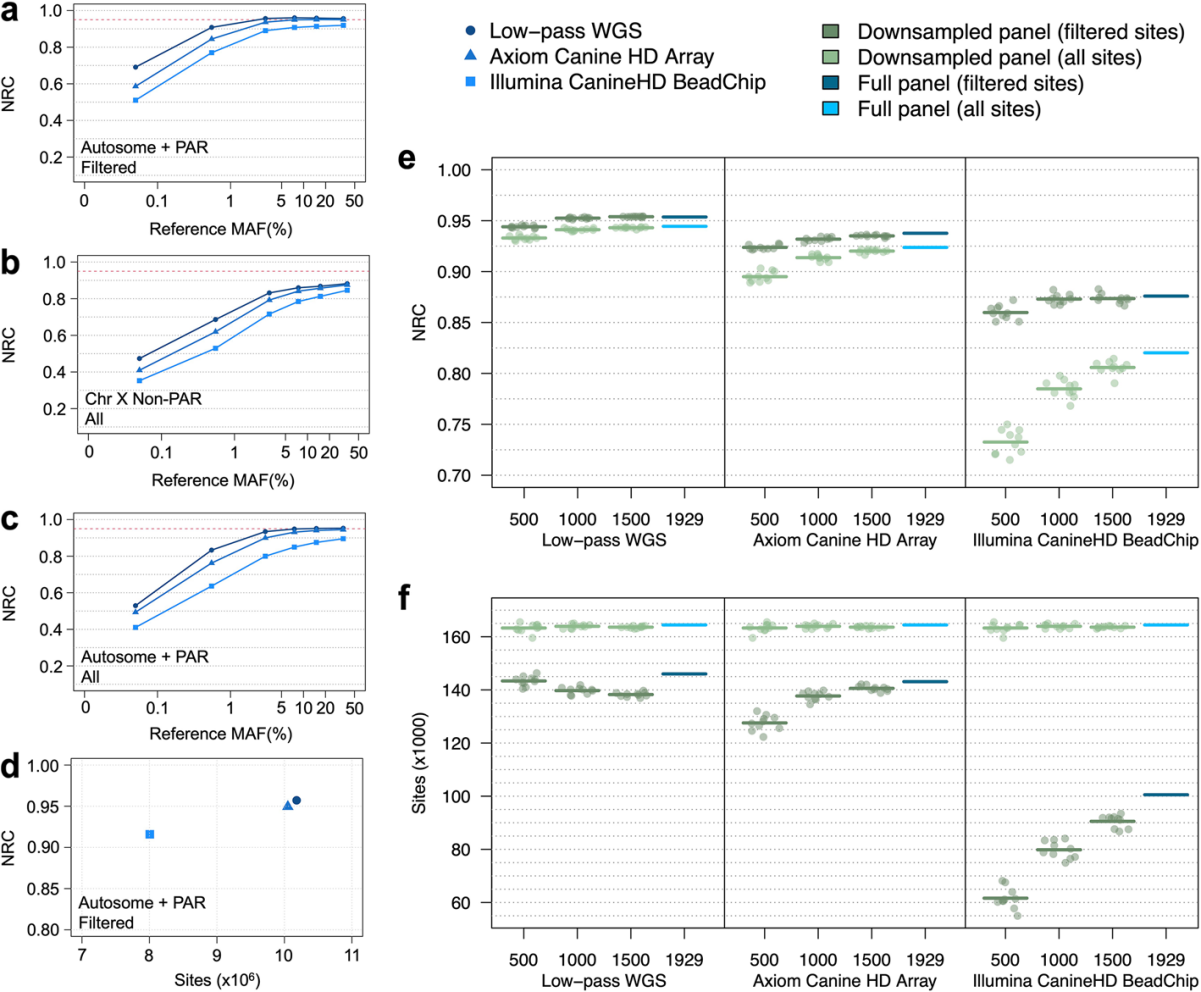
Genotype imputation accuracy of the Dog10K reference panel. **a** NRC rates of imputed genotypes across autosomes and the PAR segment of chromosome X. Variant sites are filtered according to GLIMPSE and IMPUTE5 imputation quality scores (INFO > 0.9). **b** NRC rates of imputed genotypes across the non-PAR segment of chromosome X. Variants are not filtered by imputation quality score, as imputation software does not provide scores for haploid genotypes. **c** NRC rates of imputed genotypes across autosomes and the PAR segment of chromosome X prior to filtering on imputation quality. **d** NRC rates and total number of imputed sites for each platform. Sites were filtered according to imputation quality score > 0.9 and reference MAF > 1%. **e** NRC rates for downsampled and full chromosome 38 reference panels for sites with reference MAF > 1%. Results show both quality and non-quality filtered sites. Data points show NRC rates for a single downsampled reference panel. Horizontal bars indicate mean NRC rates for each reference panel population size. **f** Number of imputed variants for downsampled and full chromosome 38 reference panels for sites with reference MAF > 1%. Results show both quality and non-quality filtered sites. Data points show the number of imputed variants for a single downsampled reference panel. Horizontal bars indicate the mean number of variants for each reference panel population size

These errors do not affect the main results and conclusions of the paper. The original article [1] has been updated.

### Supplementary Information

**Additional file 2:** Supplementary Methods [165-182]. **Fig. S1.** Imputation accuracy of individual samples for sites with MAF > 1%. **Fig. S2.** Median copy-number across the genome for wolves. **Fig. S3.** Repeatmasker classification of SINE variation. **Fig. S4.** Distribution of variation across the genome for breed and other dogs (*n*=1,591). **Fig. S5.** Nucleotide diversity along chr26. **Fig. S6.** Signature of a retrogene detected at the TEX2 locus.
