# Supplementary material for: Genome sequencing of 2000 canids by the Dog10K consortium advances the understanding of demography, genome function and architecture
Source: Genome Biol. 2023 Aug 15;24:187. doi: 10.1186/s13059-023-03023-7 (PMC10426128; doi:10.1186/s13059-023-03023-7)
Supplement: Supplementary file 3 — Additional file 3. Review history. [file 13059_2023_3023_MOESM3_ESM.docx]

Review History

**First round of review**

**Reviewer 1**

**Were you able to assess all statistics in the manuscript, including the appropriateness of statistical tests used?**

Yes, and I have assessed the statistics in my report.

**Comments to author:**

The Dog10K project aims at collecting whole-genome sequence data from 10K dogs and their wild undomesticated realtives. This paper describes first results from the analysis of 34 million sequence variants discovered in almost 2'000 canids. Authors look into genetic diversity, relationships between breeds and wild canids, and signatures of selection. Moreover, the release of comprehensive raw and processed data by Dog10K (including variant calls in VCF files which may serve as future reference panels) is highly commendable.

While the effort to compile a comprehensive catalogue of variants across canids is commendable, the present paper lacks novel biological insights that are gained from the data. I think the authors should make a strong effort to strengthen this point, for instance by prioritizing candidate causal variants underpinning the detected signatures of selection.

The authors release comprehensive variant data in VCF format. A vignette how these data can be used to impute sequence variant genotypes into low-pass sequenced or array-typed animals might further increase the appeal of the paper.

Here are a number of questions as I encountered them in the manuscript.

line 78: I'm not sure the first sentence in the introduction is really needed and supported by the following sentences.

line 80: close square bracket

line 107-109: This is an odd statement. I would argue that a pangenome approach would make use of these resources, but I don't quite get how reference-guided alignment and variant calling against one assembly (which inevitably suffer from reference bias) can help realizing the potential of the new assemblies.

line 113: be better to replace aesthetics by morphological traits or sth. similar (?)

line 120: please define structural variants and explain SNVs (is the 'S' for small or single, i.e., do you also consider small insertion and deletion polymorphisms). It may also be useful to report that all analyses are based off short read sequencing data. According to line 152 / line 160 it seems you differentiated between single nucleotide variants (SNV) and small indels? Why did you exclude small insertions and deletions?

line 119-136: Previous work from a subset of the authors (https://doi.org/10.1038/s41467-019-09373-w) investigated 91 million sequence variants identified from 722 canine whole-genome sequences. Several other efforts sequenced hundreds of dog genomes. Would be good if the authors presented some numbers on how many of the 2k canid genomes reported herein are 'novel', i.e., have not been analysed before; and how many of them were analysed earlier, and by which studies? Why is the number of variants reported here so small despite the large number of high coverage samples?

line 146: revise sentence - the GATK workflow is independent from the raw sequence alignment method

line 160: you report 33.3 million variants, which is only a third compared to a previous report (doi.org/10.1038/s41467-019-09373-w). How many of the samples and variants overlap between the current study Plassais et al? Why is there such a huge difference in variant counts between the studies?

line 193: Is it appropriate to draw such conclusions from a Venn-diagram (Figure 2D) that shows overlap of variants between differently-sized groups?

line 203-213: I'm not quite sure about the implications of this paragraph and the message the authors want to convey? Unless this statistic provides useful information (which I am not aware of), I suggest to delete.

line 224: refs 42,43 provide an Ne estimate of 15-30. How is it possible that 5 individuals capture 98.4% of all variants for a population with Ne 15-30? This casts some doubt on the results presented in lines 215 - 229.

line 378-384: the numbers presented here appear very high. To put them a bit into context, the authors report 40k SVs in an average dog genome, where deletions are more frequent than insertions. The high deletion/insertion ratio is a typical bias for short read based SV analysis. What does it mean that a typical canid genome has in excess of 60 Mb deleted sequence? In contrast, the human pangenome consortium reports less than 20k SVs per genome, with insertions being more frequent than deletions (https://www.biorxiv.org/content/10.1101/2022.07.09.499321v1). Can the authors present some indication on the reliability of SV genotyping in their cohort? To improve the SV analyses, I suggest to use them also to screen for sigantures of selection. This could be particularly relevant for signatures of selection that are driven by SVs that are not well tagged by SNVs.

line 428-452: The signature of selection analyses agree well with previous findings, but they don't really reveal anything novel. Do the data permit the fine-mapping of some of these regions and prioritization of candidate causal variants? Otherwise, there are not really any novel insights presented in these paragraphs.

line 429: check sentence

line 431: which morphological features are shared among these nine groups?

line 436: what does 'after Bonferroni correction in the ancestral component' mean?

line 488: How much did the concordance (99.8%) improve compared to the full data set that contained 33,374,496 variants? I have to admit that I find it odd to present 33.3M variants throughout the manuscript but now apply filters as they might contain false positives. Wouldn't it make more sense to exclude low-quality variants from the very beginning?

line 491-493: How many of them overlap with the 91 million variants reported earlier by a subset of the authors (https://doi.org/10.1038/s41467-019-09373-w)?

line 612/616: is canids and canines used interchanegably?

**Reviewer 2**

**Were you able to assess all statistics in the manuscript, including the appropriateness of statistical tests used?**

No, I do not feel adequately qualified to assess the statistics.

**Comments to author:**

The presented manuscripts details aspects of an analysis of about 2000 dog genomes within the 10K Dog project.

Propoably due to the nature and the stage of the 10K project a lot of the presented material is quite descriptive. For instance sentences like "A total of 11.8% of the variants are found only in village dogs, which represent 14.6% of the samples (281/1,929)." stand alone without trying to genrate a greater picture of what is actually novel and noteworthy.

In the abstract it was mentioned that the dataset "reveals fine patterns of population history". However in the main text I did not find on this topic. The section "Runs of homozygosity within sample categories" mentions "history of population size change"

"The history of population size change, selection, and breed formation has resulted in distinct levels of homozygosity among canids (44-46). Across the Dog10K collection there is a wide range in the fraction of the genome present in runs of homozygosity (ROH), with coyotes possessing the smallest total average ROH length (45.2 Mb), and breed dogs having the largest (665Mb) (Fig. 3, Table 1). As expected, the Norwegian Lundehund again has the largest number of ROH bases ..."

but this then again very descriptive. The cited figure 3 is more or less useless. The information it carries is quite low, i.e. just a ranking of the species with respect to the mean F_ROH, where the species names are abrviated and very hard to guess. I think a table would be more appropriate unless one adds more information using colors, for instance to indicate the sample to some groups (breeds, village dogs, wolves, coyotes or the color scheme given in figure 4?). The colums in Table 1 seem to be shifted for some rows.

In the paragraph about the mitochondrial genome the text says that it would offer "a unique perspective on canid relationships." Howver again facts are presented but not put into context to other evidences about relationships among dogs. What is unique here?

I further read about a locus P2RX7 implicated in gliomas. The authors write "This could be an example of the so-called "hitch-hiking", in which strong selection for a breed-defining trait, in this case aspects of skull shape, increases risk for a disease." There are very sophisticated tool to detect so-called "hitch-hiking" by the reduction of the heterozygocity. Nowadays such a sentence should not be in conjuctive.

**Authors Response**

**Point-by-point responses to the reviewers’ comments:**

Reviewer comments are given verbatim in *blue italics*.

***Reviewer #1***

*The Dog10K project aims at collecting whole-genome sequence data from 10K dogs and their wild undomesticated relatives. This paper describes first results from the analysis of 34 million sequence variants discovered in almost 2'000 canids. Authors look into genetic diversity, relationships between breeds and wild canids, and signatures of selection. Moreover, the release of comprehensive raw and processed data by Dog10K (including variant calls in VCF files which may serve as future reference panels) is highly commendable.*

*While the effort to compile a comprehensive catalog of variants across canids is commendable, the present paper lacks novel biological insights that are gained from the data. I think the authors should make a strong effort to strengthen this point, for instance by prioritizing candidate causal variants underpinning the detected signatures of selection.*

We appreciate the reviewer’s perspective and have worked to revise the abstract and manuscript text to increase clarity and to highlight the most important analyses. As suggested by the reviewers and the editor, we have performed a detailed assessment of the use of Dog10K data for genotype imputation and have attempted to fine-map the loci driving the selection signals we detected. Please see our responses to the other queries from both reviewers for detailed descriptions of these findings.

*The authors release comprehensive variant data in VCF format. A vignette how these data can be used to impute sequence variant genotypes into low-pass sequenced or array-typed animals might further increase the appeal of the paper.*

As the reviewer and editor suggested, we performed an analysis of the use of Dog10K data for genotype imputation. We assessed accuracy by down sampling WGS data from additional samples that are not included in the panel and demonstrate that a non-reference concordance rate of 0.95 can be achieved. We further show that imputation panel size leads to an increase in the total number of imputed variants with high quality scores.

These new findings, including a new figure and an additional supplementary figure, are described in the results and discussion section.

Pg 19:
“The size and breed diversity within the Dog10K dataset provide an excellent opportunity for genotype imputation. The Dog10K reference panel includes all 1,929 samples phased for biallelic SNVs with a missing genotype rate <5%. To test imputation utility, we analyzed 10 publicly available WGS samples; selected to include five breeds included in the Dog10k collection (Table S6). Data from each WGS sample were downsampled to represent three separate genotyping platforms; i) low-pass WGS, ii) Axiom Canine HD Array and iii) Illumina CanineHD BeadChip. Imputation accuracy was positively correlated with platform variant density. For example, imputation based on autosomal and X-PAR sites from low-pass WGS data achieved non-reference concordance (NRC) rates of 0.95 using a reference MAF>1%. Accuracy rates were maintained for genotypes imputed from the Axiom Canine HD Array sites, but only at a higher reference MAF (>5%) (Fig. 5a). In contrast, the X chromosome non-PAR had lower imputation accuracy for all three platforms (NRC rates<0.90, Fig. 5b). Requiring an INFO score >0.9 improved NRC rates across all platforms, with the largest gain noted for rare alleles (reference MAF<1%) (Fig 5a,c). Accuracy rates were similar between the majority of individuals, regardless of whether the imputed individual’s breed was represented in the Dog10K reference panel or not (Figure S1).

We next tested the impact of the Dog10K imputation reference panel size on imputation quality and genotype ascertainment. Here, chr38 genotypes from the publicly accessed samples were assessed. From the full Dog10K panel, ten reference panels were created for each of 500, 1,000, or 1,500 randomly selected individuals. Independent of the modeled genotype platform, the larger reference panels show increased imputation accuracy, although the gains in NRC rates were reduced using panel sizes >1,000 (Fig. 5e). Specifically, NRC rates differed by only 0.001 between the 1,000 and 1,929 sample panels. Compared to the low-pass WGS platform, NRC rates for the Axiom Canine HD Array and Illumina CanineHD BeadChip array differed by 0.006 and 0.003, respectively. Despite small gains in NRC rates, the larger reference panels revealed increased counts of imputed variants with high quality scores. For example, the transition from 1,000 to 1,929 samples resulted in the ascertainment of 6,268 chr38 variants for the low-pass WGS platform, 5,394 for the Axiom Canine HD Array platform and 20,707 for the Illumina CanineHD BeadChip platform (Fig. 5f). “

Pg 41:

“Our analyses demonstrate the utility of the Dog10K variant dataset as a reference panel for use in genotype imputation (105), an approach which has been shown to be effective in making use of low pass or poor quality sequence data (106,107). Canine studies have successfully incorporated this approach (108–111), particularly for disease GWAS, leading to identification of a risk haplotype for congenital laryngeal paralysis in Alaska sled dogs (112), and a locus for canine idiopathic pulmonary fibrosis in West Highland white terriers (108), among others.

The largest previous study, based on a panel of 676 dogs from 91 breeds with 97 high-coverage WGS dog samples downsampled to approximately 1x coverage per sample, demonstrates that both quality filtering and MAF were critical to accuracy (111). Both affect power to conduct successful GWAS, with a previous study demonstrating that as the MAF difference between cases and controls is reduced, the number of samples required for imputation of low-pass WGS to reach the same power in a GWAS as high-coverage WGS grows exponentially (111). While this study suggested discarding sites with a MAF <0.05, our data argues for selecting variants with imputation quality >0.90 and reference MAFs >1%. This reflects both the large number of dogs and breeds as well as the variation captured in village dogs in our dataset, both of which are critical for the development of any reference panel, in dogs (113,114) or otherwise (115). For the Illumina CanineHD BeadChip platform, the criteria we propose will provide imputed genotypes with NRC rates >0.85 for over 8M sites, whereas for the low-pass WGS and Axiom Canine HD Array platforms, these criteria provide NRC rates of approximately 0.95 for over 10M sites (Fig. 5d). It is important to note however, that any imputation analysis is only as accurate as the samples in the reference panel, and expansion of even a large panel, as we present here, is an important long term goal.”

3

**Fig. 5. Genotype imputation accuracy of the Dog10K reference panel**. **a.** NRC rates of imputed genotypes across autosomes and the PAR segment of chromosome X. Variant sites are filtered according to GLIMPSE and IMPUTE5 imputation quality scores (INFO>0.9). **b.** NRC rates of imputed genotypes across the non-PAR segment of chromosome X. Variants are not filtered by imputation quality score, as imputation software does not provide scores for haploid genotypes. **c.** NRC rates of imputed genotypes across autosomes and the PAR segment of chromosome X prior to filtering on imputation quality. **d.** NRC rates and total number of imputed sites for each platform. Sites were filtered according to imputation quality score >0.9 and reference MAF>1%. **e.** NRC rates for downsampled and full chromosome 38 reference panels for sites with reference MAF >1%. Results show both quality and non-quality filtered sites. Data points show NRC rates for a single downsampled reference panel. Horizontal bars indicate mean NRC rates for each reference panel population size. **f.** Number of imputed variants for downsampled and full chromosome 38 reference panels for sites with reference MAF>1%. Results show both quality and non-quality filtered sites. Data points show the number of imputed variants for a single downsampled reference panel. Horizontal bars indicate the mean number of variants for each reference panel population size.

4

**Figure S1. Imputation accuracy of individual samples for sites with MAF > 1%.** Samples along the x-axis are ordered according to breed membership within the Dog10K reference panel and NRC rates of the Illumina CanineHD BeadChip platform in A. Sample names are colored according to sex, where red sample names are males. (A) NRC rates of quality filtered and (B) non-filtered imputed genotypes across autosomes and the PAR segment of chromosome X. (C) NRC rates of imputed genotypes across the non-PAR segment of chromosome X.

5

*Here are a number of questions as I encountered them in the manuscript.*

*line 78: I'm not sure the first sentence in the introduction is really needed and supported by the following*

*sentences.*

As the reviewer suggests we have revised the indicated sentence.


“Recent advances in comparative genomics have enhanced the utility of the domestic dog and other

canines for studies of mammalian biology, disease and domestication.”

*line 80: close square bracket*

We have added the missing bracket.

*line 107-109: This is an odd statement. I would argue that a pangenome approach would make use of these resources, but I don't quite get how reference-guided alignment and variant calling against one assembly (which inevitably suffer from reference bias) can help realizing the potential of the new assemblies.*

The reviewer raises an important point about the value of pangenome approaches that directly utilize the long-read assemblies. Unfortunately, canine assemblies are not at the same level as in humans and are not phase-resolved. All of the new long-read assemblies are massive improvements over canFam3.1 with better representation of promoters, regulatory elements, and exons and thus aligning Illumina reads to a new reference offers many advantages. We meant that mapping Illumina reads to one of these assemblies would be a clear improvement over canFam3.1. We have revised the indicated text to clarify.


“Although phase-resolved canine assemblies are not currently available, the continued development of long-read assemblies will enable future analyses of variation using a pangenome approach (9). In this study we discover and characterize canine variation through alignment of Illumina sequencing reads to the recently published assembly of Mischka, a German Shepherd Dog (UU_Cfam_GSD_1.0) (28)”

*line 113: be better to replace aesthetics by morphological traits or sth. similar (?)* As the reviewer suggests we have revised the indicated text.

Pg 5:

“...collectively spanning variation in morphology, disease susceptibility, and behavior.”

*line 120: please define structural variants and explain SNVs (is the 'S' for small or single, i.e., do you also consider small insertion and deletion polymorphisms). It may also be useful to report that all analyses are based off short read sequencing data. According to line 152 / line 160 it seems you differentiated*

*between single nucleotide variants (SNV) and small indels? Why did you exclude small insertions and deletions?*

As the reviewer suggested, we clarified the text in this section to state that variants were discovered using Illumina sequencing, report the number of small indels discovered, define structural variation, and define SNV.

The discovery of indel variants is further described in Supplementary Material Section 2. We have added to this section to clearly report the total number discovered and to state the limitations of the indel call set. Unfortunately, there is not a “gold standard” truth set of indels in canines that can be used to robustly train filters. As a result, we employed hard filters following the GATK best practices and most of our analyses focus on SNV and SVs.

Pg 6:

“In the analysis herein, we present Illumina sequencing data from 1,987 canids, with joint calling across the mitochondrial and nuclear genomes revealing over 144,000 structural variants (deletions, insertions, duplications, and inversions ≥50 bp in size), 14.4 million indels, and 34 million single nucleotide variants (SNVs); the most extensive variant catalog produced in canines to date.”

*line 119-136: Previous work from a subset of the authors (*[*https://doi.org/10.1038/s41467-019-09373-w)*](https://doi.org/10.1038/s41467-019-09373-w)) *investigated 91 million sequence variants identified from 722 canine whole-genome sequences. Several other efforts sequenced hundreds of dog genomes. Would be good if the authors presented some numbers on how many of the 2k canid genomes reported herein are 'novel', i.e., have not been analysed before; and how many of them were analysed earlier, and by which studies? Why is the number of variants reported here so small despite the large number of high coverage samples?*

We appreciate the reviewer’s point that direct comparisons among variant data sets are complicated by differences in the sample acquisition and data processing of each set. For example, the Plassais et al. CanFam 3.1 referenced dataset (which we refer to as the ‘NIH Dataset’ in our manuscript) also included variants discovered from aligning diverged outgroup species, i.e. coyote (*Canis latrans*), Golden Jackal (*Canis aureus*), dhole (*Cuon alpinus*), and Andean fox (*Lycalopex culpaeus*), which resulted in the discovery of a lot more variation than that found only in dogs and wolves. In addition, the Plassais dataset also had a minimum depth filter of 2x, while Dog10K is 10x, and used different settings for base recalibration and SNV filtering.

In our analyses of function, we aimed to provide the community with a panel of normal variation that could be used for functional prioritization, and so only considered SNVs contributed by either wolves or dogs. The species contribution and SNV counts available were noted in the methods and in Fig. 9 of the revision, but we have now made explicit reference to these in the results as shown below. We have also included updated analyses as suggested by the reviewer, summarizing how each public panel and Dog10K was sampled and processed, including the depth and site filters used for variant calling (Table S12). We also assessed the individual and breed groups shared between the sets (Table S13 and Supplementary Section 11), highlighting the diversity and uniqueness of breed groups included in Dog10K.

Pg 30

“Panels of normal variation are key to prioritizing SNVs for downstream functional analyses. We compared the composition and biallelic SNV sites contributed from only dogs and wolves (when known) for three such panels, DBVDC (590 samples, 20,443,472 SNVs) (6) NIH panel (715 samples, 18,468,060 SNVs) (4) and the European Variation Archive (EVA) RS Release 3. Table S12 summarizes the sample acquisition and distinct alignment and site filtering strategies for each panel. These factors, including minimum coverage depth (ranging from 2x-10x), impact the number of samples and variants available for downstream analyses (Supplementary Information Section 11).”

Pg 31:

“This variation is in part a reflection of the diversity and uniqueness of the dog breeds included in Dog10K (60% of breeds are only found in the Dog10K collection, Table S13), as well as the limited sample sharing between this and the other sets (only 10/1929 samples were shared; Supplementary Information Section 11).”

Pg 55:

“The strict-filtered Dog10K dataset was compared to three other publically available datasets in multiple ways, i) methods used to call variants within each set, ii) sharing of individuals between sets and iii) sharing of breed types. The sets were strict-filtered Dog10K VCF (1929 samples, 28,725,482 SNVs), DBVDC (590 samples, 20,443,472 SNVs) (6), NIH (715 samples, 18,468,060 SNVs) (4) and EVA v3 (4,548,628 SNVs) (<http://ftp.ebi.ac.uk/pub/databases/eva/rs_releases/release_3/by_species/> canis_lupus_familiaris/). CanFam3.1 referenced datasets were lifted to UU_Cfam_GSD_1.0 coordinates, with variants on unplaced scaffolds excluded from further analysis. The full NIH panel contains multiple canid outgroups (Table S12). These were removed, allowing for the comparison of positions variable in dogs and wolves. For i) the methods and filters used to call variants was tabulated, and due to this variability, for ii) individuals were considered shared between datasets if their proportion of IBD was in excess of that observed for the closest pair in the Dog10K dataset (i.e.,PLINK (v1.9) (128) PiHAT > 0.9451 based on 145,845 random SNVs). For iii) breed types, breed names and descriptors were harmonized, and compared across sets.”

”

8

*Inline figure 11.1. Samples shared between three large datasets based on proportion of IBD. Total number of samples per dataset is indicated.*

*line 146: revise sentence - the GATK workflow is independent from the raw sequence alignment method* As the reviewer suggests, we have revised the indicated section to clarify that bwa-mem2 was used for alignment followed by GATK for subsequent processing (i.e, duplicate marking and base quality recalibration) and variant discovery. Details on specific program versions and options are given in Supplementary Material Sections 1 and 2.

Pg 7:

“A pipeline based on bwa-mem2 and the GATK best practices was used for the uniform sequence alignment and processing across four centers (Supplementary Information Section 1) (34–36). Variant calling (mitochondrial genome: SNVs and indels; nuclear genome: SNVs, indels, and SVs) and quality filtering were performed across the entire sample set.”

*line 160: you report 33.3 million variants, which is only a third compared to a previous report (*[*doi.org/10.1038/s41467-019-09373-w)*](http://doi.org/10.1038/s41467-019-09373-w))*. How many of the samples and variants overlap between the current study Plassais et al? Why is there such a huge difference in variant counts between the studies?* As described in the related point above, the Plassais et al. data set includes variants discovered by aligning diverged species (i.e., coyote (*Canis latrans*), Golden Jackal (*Canis aureus*), dhole (*Cuon alpinus*), and Andean fox (*Lycalopex culpaeus*)) to the dog reference genome and thus contains many SNVs due to this divergence. When limited to only dogs and wolves, the Plassais et al. data set contains 18,468,060 SNVs. As the reviewer suggested, we performed a further analysis of potential sample overlap and found that 10 samples, or close relatives, are in common. Please see the additional discussion from the related query above for more detail.

*line 193: Is it appropriate to draw such conclusions from a Venn-diagram (Figure 2D) that shows overlap of variants between differently-sized groups?*

We appreciate the reviewer’s point that the indicated analysis does not explicitly speak to the effects of domestication and breed formation on genetic diversity. We have removed the indicated sentence to focus on a description of our findings rather than speculation as to their cause.

*line 203-213: I'm not quite sure about the implications of this paragraph and the message the authors want to convey? Unless this statistic provides useful information (which I am not aware of), I suggest to delete.*

We prefer to keep this section but would defer to the editor's judgment. Sequencing, unlike genotyping using SNP arrays, permits an analysis of rare alleles and the size and breadth of our study permit such an assessment, which has not been possible before. Rare alleles likely represent the most recent genomic mutations, and as such provide insight into recent population history. F2 sites between groups represent gene flow or admixture, the sparsity of F2 sites found among dogs and wolves indicates a lack of wolf admixture into dogs. However, large collections of wolves have shown variable levels of gene flow from

dogs, a topic of ongoing scientific interest with consequences for conservation and management strategies (for example see Pilot et al. 2018 PMID:29875809) .

*line 224: refs 42,43 provide an Ne estimate of 15-30. How is it possible that 5 individuals capture 98.4% of all variants for a population with Ne 15-30? This casts some doubt on the results presented in lines 215 - 229.*

We appreciate the reviewer’s attention to detail. We argue that these statements are actually in concordance and that potential disagreement can be explained by three factors: (1) different meanings of effective population size and associated estimates, (2) overstated precision and accuracy of effective population size estimates, and (3) details of the documented breed history that are consistent with our findings.

Effective population size, Ne, is a notoriously unclear concept that is often defined as the size of an idealized Wright-Fisher population that experiences the same amount of genetic drift as the studied population. However, different measures of drift have been proposed and the properties of a population captured by different Ne estimates are distinct. Commonly used metrics include the probability of alleles being identical by descent (i.e, the inbreeding effective population size), the variance in offspring allele frequency (the variance effective population size), a summary of the allele frequency transition matrix across generations (the eigenvalue effective population size), and the coalescent effective population size which describes expected amounts of variation under a coalescence process. In terms of the total amount of molecular variation present in a sample, the coalescent effective size may be the most relevant rather than changes in inbreeding or allele frequency trajectories. The various Ne estimates do not always agree with each other and in some cases may not exist, indicating that the underlying Wright-Fisher model is not an appropriate description of the true population dynamics. For further discussion see Ewens 1982 doi:10.1016/0040-5809(82)90024-7, Sjödin et al. 2005 PMID:15489538, Wakely and Sargsyan 2009 PMID:19001293 and Wang et al. 2016 PMID:27353047.

As the reviewer notes, Pfahler and Distl 2015 PMID:25860808 estimated an Ne of 10-13 for the Norwegian Lundehund. This estimate was derived from an estimate of linkage disequilibrium between genotyped SNPs (r^2^) and calculated as Ne=(1-r^2^)/(4cr^2^) where c is the recombination rate in Morgans. The authors assumed a constant recombination rate between SNP markers with a rate of 1 MB = 1 cM. This is a crude assumption since the recombination rate is known to be highly non-uniform in dogs (see Axelsson et al. 2012, PMID: 22006216, Auton et al 2013 PMID: 24348265, Campbell et al. 2016 PMID:27591755 and others). This nonuniformity is not captured by the utilized rate. Further, the estimate of linkage disequilibrium is derived from sites on a SNP-chip with a complex ascertainment history that was biased toward certain breeds. SNP ascertainment is known to impact estimates of linkage disequilibrium, with most ascertainment schemes resulting in decreased levels of marker correlation (Nielsen 2003, PMID:12689795). In the method used by Pfahler and Distl an underestimate of r^2^ results in an overestimate of Ne.

10

Kettunen et al. 2017 PMID: 28107382 took a different approach to estimate Ne in Lundehunds, and reported estimates of 13-82 depending on the method employed. These estimates are based on analysis of a highly complete pedigree of Lundehunds from 1930-2015 compiled by the Norwegian Lundehund breeding club. Estimates of Ne were calculated from the pedigree based on calculated changes in inbreeding through subsequent generations in the pedigree. The range of estimates reflects different ways to estimate the change in inbreeding, including differences in the range of generations in the pedigree considered. Furthermore, the methods assume that the founders of the pedigree are unrelated, an unlikely assumption given the known breed history (see below).

Kettunen et al’s analysis of the pedigree revealed highly skewed dynamics. Two individuals born in the 1960s make a dynamic contribution to the current population. The authors estimate that 76% of alleles in dogs born in 2015 originated in those two individuals. This is consistent with our finding that the vast majority of non-reference SNP alleles have already been captured in the Lundehunds.

Further consideration of the known Lundehund history is also consistent with our finding. The breed underwent sustained population decreases throughout the 19th century. By the 1940s the population was reduced to ~50 individuals. The population almost went extinct following two severe bouts of canine distemper. The breed was reduced to 5-6 surviving members, each of which were related to each other, used to recreate the breed (5 contributors are reported in Melis et al. 2013 PMID:22988964 and 6 contributors are reported in a breed compendium written by the Norwegian Kennel Club Standard Committee in 2010, <http://lundehund.no/images/Pdf-er/Breed_Compendium_Lundehund_English_2015_web.pdf).>

Together, these aspects of Lundehund history make it unsurprising that our analysis of 5 individuals has captured such a large fraction of the sequence variation in the breed. Consistent with our estimate, examination of histogram of the non-reference allele frequency shows little variation among the sampled individuals: 80% percent of non-reference alleles are homozygous in all 5 individuals, and only 2.4% percent of the total discovered variants have an allele count of one. Thus, we feel that our estimate of the variation captured in the Lundehund is consistent with the data we generated as well as with prior reported findings for this breed.

*11*

**Response Figure 1: Histogram of non-reference allele counts for autosomal SNPs found in 5 Lundehunds**. The Y axis is the number of non-reference SNPs. The X axis gives the number of times an allele was observed, ranging from 1 (heterozygous in one individual) to 10 (homozygous in all 5 individuals).

Moreover, we note that our estimate assumes that the sampled individuals are representative of the breed as a whole. Within breed population structure, such as between North American and European isolates of a breed, would bias our estimates. To clarify this point, we have added two sentences to the results that offer additional details on the Lundehunds and clarify a limitation of the method.

Pg 11:

“This reflects their well-established closed breeding population structure that was derived from 5-6

individuals (43–45).”

Pg 11:

“These estimates assume that the sampled individuals are representative of the breed as a whole, so

may be biased if there is within-breed population structure.”

*line 378-384: the numbers presented here appear very high. To put them a bit into context, the authors report 40k SVs in an average dog genome, where deletions are more frequent than insertions. The high deletion/insertion ratio is a typical bias for short read based SV analysis. What does it mean that a typical canid genome has in excess of 60 Mb deleted sequence? In contrast, the human pangenome consortium reports less than 20k SVs per genome, with insertions being more frequent than deletions (*[*https://www.biorxiv.org/content/10.1101/2022.07.09.499321v1)*](https://www.biorxiv.org/content/10.1101/2022.07.09.499321v1))*. Can the authors present some indication on the reliability of SV genotyping in their cohort? To improve the SV analyses, I suggest to use them also to screen for signatures of selection. This could be particularly relevant for signatures of selection that are driven by SVs that are not well tagged by SNVs.*

The reviewer correctly notes that the landscape of structural variation in dogs is strikingly different from the patterns reported in humans. The major driver of this difference is variation involving mobile elements, in particular dimorphic insertions of LINE1 (~6 kbp in size) and SINEC (~200 bp in size) sequences. Indeed, we find that 31.7% of deletions and 52.7% of insertions are SINEC sequences (see pg 26). This is consistent with previous findings that dimorphic mobile element insertions are dramatically more common in canines than in humans. For example, a previous comparison of a Great Dane and a Boxer genome assemblies identified 16,221 dimorphic SINEs and 1,121 dimorphic LINEs, an ~17-fold increase in SINE differences and an ~8 fold increase in LINE differences relative to that found in humans (Halo et al. 2021 PMID:33836575).

As the reviewer suggested, we assessed LD between structural variants and SNVs. Using an r^2^ cutoff of 0.8, we find that 43.8%-64.7% of SVs are in strong LD with a SNV. This is in broad agreement with similar studies of other species (i.e, Sudmant et al. 2015 PMID:26432246 and Geibel et al. 2022 PMID:35264116). The lower LD found with duplications likely reflects both a higher mutational recurrence rate and lower genotype accuracy found with this SV type.

**Inline table 7.3 Linkage disequilibrium between structural variants and SNVs**.

| **SV Type** | **Tested SVs** | **SVs with tag SNV** | **Percent Tagged** |
| --- | --- | --- | --- |
| Deletions | 68,119 | 44,068 | 64.7% |
| Insertions | 50,629 | 29,633 | 58.6% |
| Duplications | 3,005 | 1,317 | 43.8% |

LD was calculated between deletion, insertion, and duplication variants and SNVs using PLINK. Analysis was limited to autosomal structural variants with a minor allele frequency of at least 1%. SNVs within 200 kb of each structural variant were assessed with an r^2^ cutoff of 0.8.

We agree with the reviewer that the difference between insertion and deletion variants we observe reflects the limitations of Illumina short-read sequencing for the discovery and genotyping of insertion variants. As a result, we feel that a comprehensive analysis of canine structural variation will require the integration of variants discovered using long-read sequencing. Accordingly, members of the Dog10K consortium are pursuing long-read SV analysis as a separate study.

The new analysis of LD between SVs and SNVs has been added to the Supplementary Material Section 7 and briefly described in the main manuscript text.

13

Pg 24:

“We assessed linkage disequilibrium (LD) between genotyped structural variants and SNVs and found that 64.7% of deletions, 58.6% of insertions, and 43.8% of duplications are in strong LD (r^2^>0.8) with a flanking SNV. The lower levels of LD found with duplications likely reflects both a higher mutational recurrence rate and lower genotype accuracy for this SV type.”

*line 428-452: The signature of selection analyses agree well with previous findings, but they don't really reveal anything novel. Do the data permit the fine-mapping of some of these regions and prioritization of candidate causal variants? Otherwise, there are not really any novel insights presented in these paragraphs.*

In response to the reviewer's suggestion, as well as comments from the editor, we have revised our description of the selection scan and performed additional analyses to fine-map potential variants. Our fine mapping, which used the iSAFE method, identified *HMGA2* as the likely candidate for selection in the ancestral component maximized in Spitz dogs. However, for other loci we found a broad pattern of high-scoring sites that does not pinpoint a single gene. The iSAFE results are given in Table S10 and in Supplementary Information Section 8. Changes to the results and discussion related to these points are given below.

Pg 28:

“We applied iSAFE (90), a method which ranks candidate favored mutations during a selective sweep based on haplotype and allele frequency patterns, to disentangle the signature in the chr1 locus. Setting Spitz dogs as the cases and the remaining samples as the controls, we found that the sites with the highest iSAFE scores, including several sites identified by Ohana, cluster in *HMGA2.* Application of iSAFE to other loci revealed broad patterns of high-scoring variants that do not pinpoint a single gene (Table S10, Supplementary Information Section 8).”

Pg 43:

“To refine the selection candidates we applied iSAFE (90), a method for fine mapping mutations favored during selective sweeps, to the regions we identified. Our analysis nominates *HMGA2*, a known regulator of canine body size, as the likely target in the chr10 locus that was selected in the ancestral component that is maximized in Spitz dogs. However, for the remaining loci we observe a broad pattern of high-scoring candidate variants distributed throughout the candidate region. Further dissection of such loci will require combinations of selection scans, association studies with well measured phenotypes preferably including samples from multiple breeds, and functional follow up.”

*line 429: check sentence*

We thank the reviewer for identifying this jumbled sentence. Our description of the Ohana analysis has

been revised extensively. The relevant section introducing the method is given below.

Pg 27:

“To test for signatures of selection among major breed clades, we assigned 790 breed dogs into nine groups (Spitz, Sighthounds, Waterdogs, Scenthounds, Pointers, Belgian herders, UK herding dogs, Spaniels, and Mastiffs) based on genetic similarity and morphological features (Fig. 3, Supplementary Information Section 8). To balance the risks of overfitting with the interpretability of results, we focus on analysis of K=5 ancestral components. These five components are distributed across the analyzed breed dogs and are maximized in the Spitz, Mastiffs, Scenthounds, Pointers and Spaniels, and a subset of the UK Herders (Collies and Shetland Sheepdogs) (Fig. 8). Using Ohana (75), we then searched for signals of selection in each ancestral component by identifying variants with population differentiation that is not consistent with the genome-wide estimated allele frequency covariance matrix.”

*line 431: which morphological features are shared among these nine groups?*

To test for selection we divided 790 breed dogs into nine different groups (Spitz, Sighthounds, Waterdogs, Scenthounds, Pointers, Belgian herders, UK herding dogs, Spaniels, and Mastiffs). The breeds within each group are related, as determined by the clade analysis we performed. The groups differ from each other in terms of morphological and behavioral characteristics, such as ear, fur, and body size phenotypes. This reflects the clustering described in Figure 3. We hope that the revised description described above clarifies the approach.

*line 436: what does 'after Bonferroni correction in the ancestral component' mean?*

We have clarified this in the revised description of the approach. We set the significance threshold

based on the number of tests that were performed.

Pg 27:

“We set significance levels based on the number of tests performed and considered genes either overlapping or within 100kb of the significant sites as potential candidates for selection, resulting in 15 candidate loci (Fig. 8, Table S9).”

*line 488: How much did the concordance (99.8%) improve compared to the full data set that contained 33,374,496 variants? I have to admit that I find it odd to present 33.3M variants throughout the manuscript but now apply filters as they might contain false positives. Wouldn't it make more sense to exclude low-quality variants from the very beginning?*

We agree that it may not have been clear how the strict filtering affected the starting set of VQSR PASS variants and have clarified the text. Firstly, in functional inference, we only considered biallelic variants, whereas multiallelic SNVs are also in the VQSR PASS set. Second, variants in the VQSR PASS set are modeled based on a supplied true positive training set (see methods and Table S12). Unlike human data, these training sets have not been validated, and we specifically lack a true negative training set. For these reasons, for the strict-filtered data we include additional filters for properties such as allelic depth, allelic balance, and the rate of missing genotypes. In order for the reader to better assess the impact of these filters, we have also included graphs to illustrate the MAF at the removed sites (see Supplementary Information Section 9). As expected, most are rare (>1%), but the contribution is different on autosomes versus chrX. It may be that, with additional training sets and additional sequencing data, the sites removed by the strict filters will be kept in future analyses.

In terms of the concordance rates, we have updated this section to report ≥99.6% concordance rate for both the PASS and strict-filtered sets as the difference is modest and few of the sites on the Illumina Canine HD Array used for comparison are affected by these filters. Further breakdown of the concordance analysis can be found in Supplementary Information Section 9.

We have updated the main manuscript text to state that a small number of sites are affected and that these are predominantly low frequency.

Pg 30:

“These filters removed 0.7% of total available VQSR PASS sites, resulting in 27,878,361 autosomal and 847,128 chrX SNVs utilized for functional analysis (Supplementary Information Section 9). On autosomes, 78.9% of filtered sites had an observed MAF >1%, but the allelic profile for removed sites on chrX differed, with 58.5% of sites observed with a MAF>1% (Supplementary Information Section 9). Both the VQSR PASS and strict-filtered biallelic SNV sets had concordance rates ≥99.6%, based on the genotypes of 168 individuals also typed on the Illumina Canine HD Array (Supplementary Information Section 10).”

*line 491-493: How many of them overlap with the 91 million variants reported earlier by a subset of the authors (*[*https://doi.org/10.1038/s41467-019-09373-w)?*](https://doi.org/10.1038/s41467-019-09373-w)?)

As described above, the 91 million value from Plassais et al. includes several highly diverged outgroup species. When considering only sites found in dogs and wolves, this data set reduces to 18.4 million variants. We find that ~13.9 million of these SNVs overlap with our set. We note that in practice, most users of the Plassais data set, including the association analyses described in Plassais et al., do not consider the variant discovered in the outgroup species.

We also note that Plassais et al. use a minimum depth filter of 2x in their variant discovery phase, and increase this to 10x when constructing a more robust variant set for use in their GWAS pipeline. This step alone shrinks their set to ~78 million variants. This number is reduced again when filters for the rate of missing genotypes were applied for their GWAS analyses (~14 million variants, Supplementary Figure 3 of Plassais et al.)

Even though the total set of 91 million variants is not used by most users of the Plassais et al. data, and we feel that the overlap statistics we described in the related point above are more appropriate, we compared the Dog10K biallelic SNVs to this total set and found that 8.1 million SNVs are unique to the Dog10K data set.

Please see the response above for a more detailed comparison of variants and samples that overlap among data sets.

*line 612/616: is canids and canines used interchanegably?*

**16**

We thank the reviewer for identifying this ambiguity. The term “canid” refers to the species in the family *Canidae*. We have revised the indicated section to use the less ambiguous term “canid”.

Pg 40:

“..selective history of canids and serve as...”

*17*

**Reviewer #2:**

*The presented manuscripts details aspects of an analysis of about 2000 dog genomes within the 10K Dog*

*project.*

*Probably due to the nature and the stage of the 10K project a lot of the presented material is quite descriptive. For instance sentences like "A total of 11.8% of the variants are found only in village dogs, which represent 14.6% of the samples (281/1,929)." stand alone without trying to generate a greater picture of what is actually novel and noteworthy.*

We appreciate the reviewer’s perspective. We have worked to revise the abstract and manuscript text to increase clarity and to highlight the most important analyses. Please see our responses to the other queries from both reviewers for more elaboration. In addition to describing the variation obtained from uniform processing of a broad sampling of canids, we highlight several findings including:

1. An extensive assessment of the utility of Dog10K for imputation as well as release of required phased genotype files. This new analysis was performed at the suggestion of the reviewers and editor.
2. A heavily revised description of our analysis of signals of selection among major breed clades. This includes a revised presentation of the results that more clearly describes the methodology and highlights how genes involved in morphology and coloration account for many of the detected signals. We have additionally attempted further fine mapping to identify the variants or the genes that drive the signal. We couple this with additional discussion about the limitations of these approaches given the extent of linkage disequilibrium in breed dogs and suggest ways forward for the community using integrated approaches. This revised analysis was also suggested by the reviewers and the editor.
3. We performed a joint analysis of autosomal and mitochondrial variation in the same samples using a robust pipeline that we benchmark using long-read data. This is rare in the literature due to the lack of overlap between researchers focused on mitochondrial and nuclear genetic variation. We benchmark our method to reconstruct the full mitochondrial sequence using long-reads. Our key findings include the high degree of identical mitochondrial sequences found among samples and the limited correlation between mitochondrial and autosomal genetic diversity.
4. Dog10K data permit an assessment of runs of homozygosity (ROH) across the entire genome without the bias found in previous studies that used SNP arrays. Specifically, we find that the results of Mooney et al. PNAS 2021, which claims that there are some genes that are never found in ROH in dogs, potentially because of the presence of heterozygous lethal alleles, are not consistent with the genome-wide data we generated.
5. Our analysis of structural variation highlights the contribution of mobile elements to canine diversity. As reviewer 1 notes, this pattern differs from that found in humans.
6. We report a surprisingly large presence of retrogenes in dogs (from 926 different parent genes), which are biologically important on their own and also present a serious confounding factor in disease mapping efforts.
7. We perform an analysis of variation affecting pharmacogenetic and “druggable” genes, an important consideration for the use of dogs for drug discovery and clinical trials.
8. We provide a cautionary tale about the dangers of the uncritical use of existing large disease gene databases such as OMIA.
9. Our data, including constraint annotations and phased variation directly usable for imputation, are publicly available.

*In the abstract it was mentioned that the dataset "reveals fine patterns of population history". However in the main text I did not find on this topic. The section "Runs of homozygosity within sample categories" mentions "history of population size change"*

In response to the reviewer’s concerns we have reworded the abstract to better focus on our main results, including our new imputation analysis.

*"The history of population size change, selection, and breed formation has resulted in distinct levels of homozygosity among canids (44-46). Across the Dog10K collection there is a wide range in the fraction of the genome present in runs of homozygosity (ROH), with coyotes possessing the smallest total average ROH length (45.2 Mb), and breed dogs having the largest (665Mb) (Fig. 3, Table 1). As expected, the Norwegian Lundehund again has the largest number of ROH bases ..." but this then again very descriptive.*

We have reworded this statement to describe our analysis of allele sharing among groups. Note, that we have made further changes to the abstract to accommodate a description of the new imputation analyses we performed.

Pg 3:

“We have developed a dense dataset of 1,987 sequenced canids that reveals patterns of allele sharing,

identifies likely functional variants, informs breed structure, and enables accurate imputation.”

*The cited figure 3 is more or less useless. The information it carries is quite low, i.e. just a ranking of the species with respect to the mean F_ROH, where the species names are abbreviated and very hard to guess. I think a table would be more appropriate unless one adds more information using colors, for instance to indicate the sample to some groups (breeds, village dogs, wolves, coyotes or the color scheme given in figure 4?).*

We appreciate the reviewer’s comment and agree that the breadth of our data made our original representation challenging to visualize. As the reviewer suggests, we have now included a table (Table S5) that reports this information for each sample group. We have replaced Figure 3 with a revised figure that summarizes the FROH for each breed group. The extent of inbreeding varies dramatically across dog breeds, and this is a topic that is of interest to many dog researchers, both in terms of demography but also breed health. We further emphasize that our sequence-based approach, in contrast to existing SNP arrays, provides sufficient resolution to detect ROH across almost the entirety of the dog genome.

We have revised our description of the ROH analysis to clarify the trends we observed. The revised text and figure are shown below.

Pg 15:

“Runs of homozygosity (ROH) in an individual’s genome result from the inheritance of two copies of an ancestral haplotype in that individual, and so ROHs are autozygous (homozygous by descent). The estimated proportion of a genome(s) that is in ROH gives a measure of individual or population level inbreeding. For all dog breeds, selective breeding has involved some level of inbreeding and this has resulted in a wide range in ROH across breeds (47–49). For each genome in the Dog10K collection we estimated the proportion in ROH (FROH) (Table S5). This provides high-resolution estimates of historical levels of inbreeding within breeds and breed groups, as well as the genomic coordinates of regions where ROH are never found. Regions lacking ROH may indicate locations where heterozygosity is maintained for correct function. As expected, wild canids show the lowest genome proportions in ROH, with coyotes possessing the smallest total average ROH length (45.2Mb), and breed dogs having the largest (665Mb, Table 1). However, there is large variation in these averages, with some individuals and breeds showing particularly elevated and others particularly low ROH (Fig. 4, Table 1). For example, a Norwegian Lundehund had the largest number of ROH bases (total ROH=1,842Mb; FROH=78.8%), while a Saluki (sighthound) had the fewest (total ROH= 12.8Mb; FROH=0.56%).”

**FROH**

0.8

0.6

0.4

0.2

0.0

Norwegian Lundehund

Saluki

**Breed group**

**Scottish Terriers Black and Tan Terriers Alpine Irish Terriers German Shepherds Spaniels English Terriers Dutch UK Herding Belgian Herders Mastiffs Retriever None Australian Terriers Multi Purpose Scenthounds Pointers Spitz Continental Herders Water Dogs Asian American Terriers Pinscher Hungary Flockguard Sighthound Hairless**

**Fig. 4. Proportion of the genome covered by ROH (FROH).** Mean and standard deviation are plotted for breed groups. Red dashed line shows mean FROH for breed dogs; gray dashed line shows mean FROH for wolves. Breeds containing individuals with the highest and lowest FROH are labeled.

20

*The columns in Table 1 seem to be shifted for some rows.*

We thank the reviewer for catching this error. The table has been revised.

**Table 1. Runs of homozygosity (ROH) by sample category.**

|  | Average ROH |  | Total ROH |  |
| --- | --- | --- | --- | --- |
| Category | Count | Length (Mb) | Largest (Mb;  genome %) | Smallest (Mb; genome %) |
| Breed dogs | 1267 | 0.525 | 1,842; 79.6% | 12.8; 0.6% (Saluki) |
|  |  |  | (Norwegian |  |
|  |  |  | Lundehund) |  |
| Village dogs | 670 | 0.373 | 872; 37.7% (Nepal) | 8.3; 0.4% (China) |
| Wolves | 570 | 0.438 | 946; 40.9% (Sweden) | 20.2; 0.9% |
|  |  |  |  | (Tajikistan) |
| Coyotes | 152 | 0.298 | 61.2; 2.6% | 38.8; 1.7% |

The sample or breed population with the largest or smallest total amount of ROH is indicated.

*In the paragraph about the mitochondrial genome the text says that it would offer "a unique perspective on canid relationships." However again facts are presented but not put into context to other evidences about relationships among dogs. What is unique here?*

We have rewritten this sentence to improve clarity and to reference prior studies on the importance of mitochondrial variation. We meant that even though mitochondrial variation has been important for multiple lines of research (such as forensics, ancient DNA, analysis of domestication, breed relationships), mitochondrial variation has often been missing from large scale genome sequencing projects. The unique advance in our study is that we have included mitochondrial variation by implementing a new pipeline to accurately reconstruct mitochondrial variation from Illumina reads. Benchmarking of this approach using long-read data is described in Supplementary Information Section 6 and the method is summarized on pg 52. This is an important addition since previous studies often focus on the amplification of a small mitochondrial segment. Our novel findings include the high degree of identical mitochondrial sequences found among samples and the limited correlation between mitochondrial and autosomal genetic diversity.

The revised sentence clarifies the importance of including mitochondrial variation.

Pg 22:

“The mitochondrial genome is often overlooked in large nuclear genome sequencing projects despite the importance of mitochondrial variation for forensics (50–53) and for studies of ancient and modern canine diversity (10, 54–57).”

21

*I further read about a locus P2RX7 implicated in gliomas. The authors write "This could be an example of the so-called "hitch-hiking", in which strong selection for a breed-defining trait, in this case aspects of skull shape, increases risk for a disease." There are very sophisticated tool to detect so-called "hitch­hiking" by the reduction of the heterozygosity. Nowadays such a sentence should not be in conjunctive.* We appreciate the reviewer's comment about explicitly testing for hitch-hiking in this region. The region on chr26 contains a locus associated by GWAS with body size as well as a locus associated with glioma risk. Candidate genes for both traits are found in the selected region, which is quite broad. To illustrate the point we were making, we plotted nucleotide diversity (**π**) by sample clade along chr26. The putative selection signal is quite large, with a reduction in diversity in the Mastiff group extending over multiple megabases. Such long segments of extended linkage disequilibrium and correlated redactions in diversity are characteristics of breed dogs. These characteristics hinder efforts for statistically disentangling selection signals that are successful in other species. For more description of these limitations see the discussion in Schlamp et al. 2015 PMID:26589239 which assessed statistical properties around known causative loci for breed defining traits.

**Figure S5. Nucleotide diversity along chr26.** Each line depicts observed nucleotide diversity (π) found in each analyzed clade along chr26. The gray box at the left delineates the region identified as under selection in Mastiffs using the program Ohana. The gray box at the right depicts a region of increased copy-number identified in the Dog10K data. The increase in nucleotide diversity likely reflects miscalled variants due to the copy number change.

22

Text describing this issue has now been included in the discussion section.

Pg 42:

“The precise identification of the genes targeted by selection during breed formation is hindered by the extended range of linkage disequilibrium in breed dogs (116). As a result, identified loci often contain multiple genes previously associated with disparate phenotypes. For example, a large region on chr26 shows signatures of selection in the ancestral component that is maximized in the Mastiff group. This 7.5Mb region shows an extended reduction in nucleotide diversity relative to other clades (Fig. S5) and includes genes associated with canine body size and height (4) as well as glioma risk (117) and other cancer phenotypes (118–120).”

**Second round of review**

**Reviewer 1**

The authors present a revised version of their manuscript which has much improved. I appreciate the authors' attention to all comments raised and think that the revised manuscript addresses most previous concerns.

I have two (minor) comments on the indels left, which have not been resolved in the manuscript.

I find it still very difficult to assess at many places in the manuscript if the analyses are based on SNPs, SNP and indels, or SNPs and SVs. Most analyses seem to be conducted with SNPs, but to resolve this and help the reader, I suggest to add an introductory statement at an appropriate place in the manuscript to explain and justify that all subsequent analyses (unless stated otherwise) are based on SNPs (e.g., at line 170). Also, it appears that authors use SNV, biallelic sites, sites, etc. interchangeably. If this is the case, then I suggest to stick to one term e.g., SNV and use this throughout.
Here are some examples, where I found the wording a bit unclear:
line 64: you mention only single nucleotide and SVs, but not indels.
line 131: 14.4 million indels are mentioned here, but not in abstract
line 170ff: still not clear why only SNVs are reported, but not small indels. If analyses in this and subsequent paragraphs are done with SNVs only, the authors may want to add an introdcutory sentence explaining and justifying this.
line 204: biallelic autosomal polymorphic sites - do you refer to indels, SNVs, or SVs, or all types of variants here?
line 928: «total 29,234,830 autosomal, and 965,534 chrX sites are included» - what sites are you referring to, SNVs, SNVs and indels?


The authors argue that no truth set is available for indels, so they excluded them from most analyses. The same is arguably also true for SVs, but they are included in (some of) the analyses. I think there's still more explanation needed in the manuscript regarding the high number of indels reported (14.4 M indels vs. 34.4 M SNVs). Large studies in humans, horse, and cattle identified between 10- and 20-fold more SNPs than Indels (https://www.nature.com/articles/sdata201511, https://onlinelibrary.wiley.com/doi/10.1111/age.12753, https://www.nature.com/articles/ng.3034). A previous study in in dogs reported 5-fold more SNPs than indels (https://www.nature.com/articles/s41467-019-09373-w). So there's a clear excess of indels in the current study. Is this due to flaws in the reference genome used? How does this agree with line 174ff (96% of the assembly is amenable to short read calling)?

**Authors Response**

**Point-by-point responses to the reviewers’ comments:**

In the revised manuscript we have responded to the remaining reviewer requests. The changes are outlined below:

We have made several wording changes to include indel variants in the total variants
reported and to clarify when only single nucleotide changes are considered. These
include:
a. The abstract now states “>48M single nucleotide, indel, and structural variants”
b. On pg. 8 we explicitly state the total number of indels found and justify the focus
on SNVs. The revised text is: “Using hard filters, we identify a total of
14,414,501 indel and mixed variants. Subsequent analyses are focused on SNVs
due to the paucity of validated canine indels available to train refined filters.”

c. On pg. 10 we clarify that we are referring to “biallelic autosomal SNV sites”
d. On pg. 11 we clarify that we are estimating the number of SNVs that remain to be
discovered: “we estimated the total number of SNVs expected...”
e. On pg. 36 we clarify that we are analyzing SNVs: “Using the VQSR PASS SNV
VCF as an input...”
f. On pg. 40 we include indels in the reported total: “more than 48 million SNVs,
indels, structural variants...”
g. On pg. 52 we clarify in the methods that the imputation analysis is based on SNVs
h. We added a new paragraph on pg. 41 that incorporates the reviewer’s idea to
compare the ratio of SNVs to indels across studies and to comment on differences
in the observed ratio. The new paragraph is:
“Our variant filtering pipeline leveraged sites routinely genotyped in
commercial arrays as a training set to identify 34.5 million high-quality
SNVs. Since a robust truth set is not available for indel variants, we
applied hard filters based on criteria recommended by the GATK best
practices to identify 14.4 million indels. The indel total includes sites with
a mixture of SNV and indel alleles. Our indel to SNV ratio of 2.4 is
similar to that reported by two other recent surveys of dog and wolf
variation [6, 30]. However, another study of canines, which included
additional outgroup samples from the Canis, Cuon, and Lycalopex genera,
reports an SNV to indel ratio of 4.2 [4], while studies of equines [108],
bovines [109], and humans [110] report SNV to indel ratios greater than
10. It is not clear to what degree the apparent excess of indel variation in
canines reflects true biological differences or technical artifacts in calling.
Given this uncertainty, our analysis is primarily focused on SNVs.”
