# Supplementary material for: Author Correction: Genome sequencing of 2000 canids by the Dog10K consortium advances the understanding of demography, genome function and architecture
Source: Genome Biol. 2023 Nov 7;24:255. doi: 10.1186/s13059-023-03101-w (PMC10631033; doi:10.1186/s13059-023-03101-w)
Supplement: Supplementary file 1 — Additional file 2: Supplementary Methods [165-182]. Fig. S1. Imputation accuracy of individual samples for sites with MAF > 1%. Fig. S2. Median copy-number across the genome for wolves. Fig. S3. Repeatmasker classification of SINE variation. Fig. S4. Distribution of variation across the genome for breed and other dogs (n=1,591). Fig. S5. Nucleotide diversity along chr26. Fig. S6. Signature of a retrogene detected at the TEX2 locus. [file 13059_2023_3101_MOESM1_ESM.pdf]

Additional file 2: Supplementary Information

SUPPLEMENTARY FIGURES 3

INDEX OF SUPPLEMENTARY TABLES 9

SUPPLEMENTARY METHODS 10

Section 1: Reference genome construction and sample processing 10

- Samples available for processing 10
- Genome assembly used for read alignment 10
- Identification of the pseudoautosomal region 10
- Read alignment 12
- Sequence quality analysis 14
- Identification of duplicate and mislabeled samples 16

Section. 2 Genome wide identification of SNVs and indels 19

- Variant calling 19

Section 3 Breed relationships and haplotype sharing 22

- Breed cladogram 22
- D-statistics 22

Section 4 Variation among breeds 24

- Allele sharing 24
- Estimation of variation to be discovered 25

Section 5 Runs of homozygosity 27

- Identifying ROH regions 27

Section 6 Analysis of mitochondrial sequence 31

- Pipeline overview 31
- Pipeline validation 32
- Analysis of Dog10K samples 34

Section 7 Structural variation analysis 38

- QuicK-mer2 analysis 38
- Structural variant analysis with Manta 41
- A locus identified with QuicK-mer2 and Manta. 43

Section 8 Selection in breed groups 45

- Analysis using Ohana 45
- Fine-mapping using iSAFE 48

Section 9. Strict filtering 55
Section 10. Variant concordance 57
Section 11. Comparison of public variation catalogs 60
Section 12. Genome-wide distribution of genetic variation 61
Section 13. Druggable gene target analysis 65

SUPPLEMENTARY FIGURES

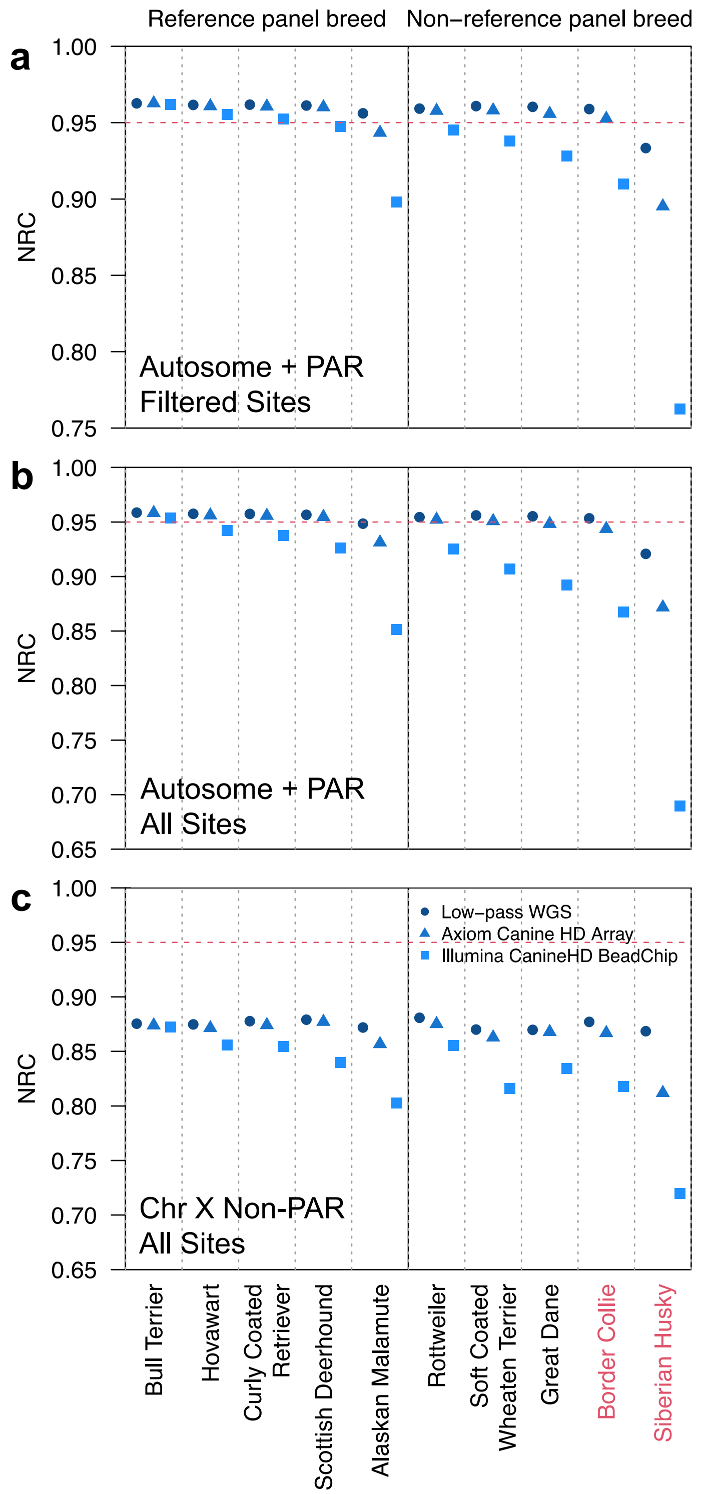

Fig. S1. Imputation accuracy of individual samples for sites with MAF > 1%. Samples along the x-axis are ordered according to breed membership within the Dog10K reference panel and NRC rates of the

Illumina CanineHD BeadChip platform in A. Sample names are colored according to sex, where red sample names are males. (A) NRC rates of quality filtered and (B) non-filtered imputed genotypes across autosomes and the PAR segment of chromosome X. (C) NRC rates of imputed genotypes across the non-PAR segment of chromosome X.

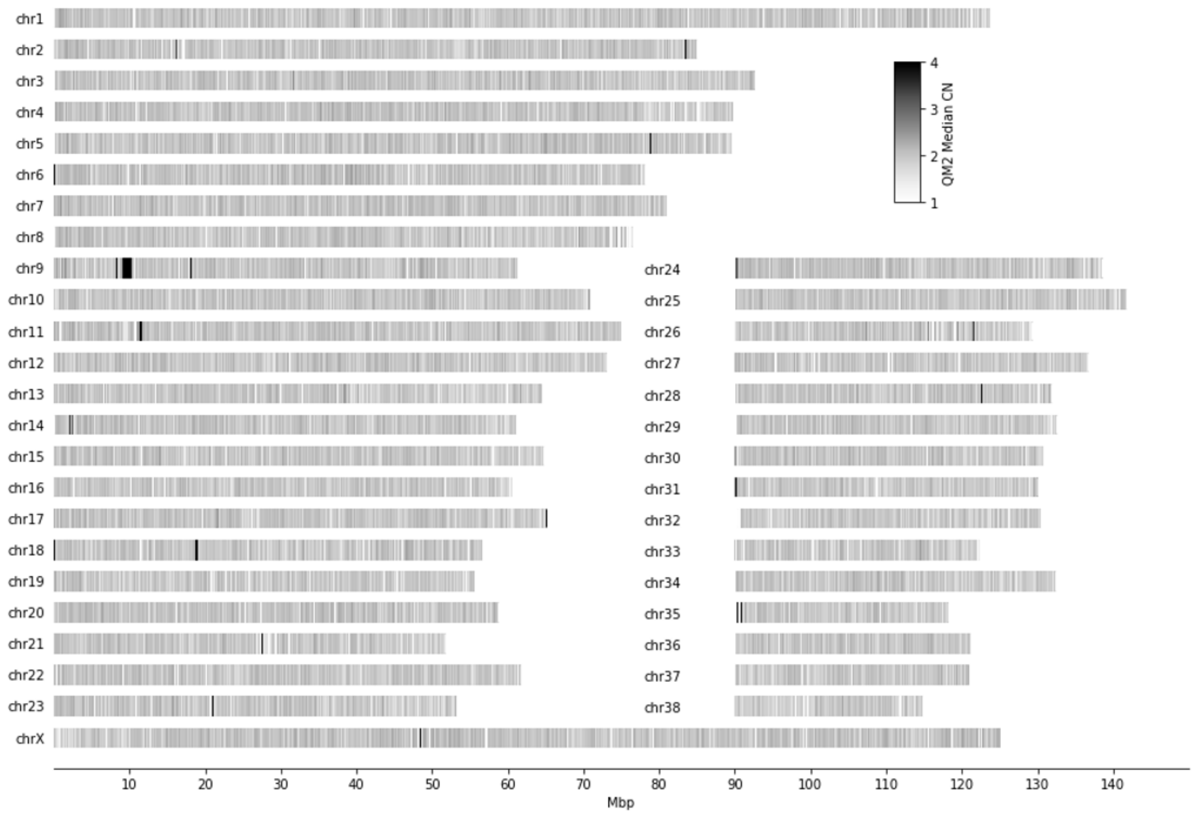

Fig. S2. Median copy-number across the genome for wolves.

The median copy-number estimated by QuicK-mer2 across the genome for wolves is shown. A wolf-specific duplication is apparent on chr26. Note that all estimates are based on depth at 30-mer sequences that are unique in the UU_Cfam_GSD_1.0_ROSY assembly.

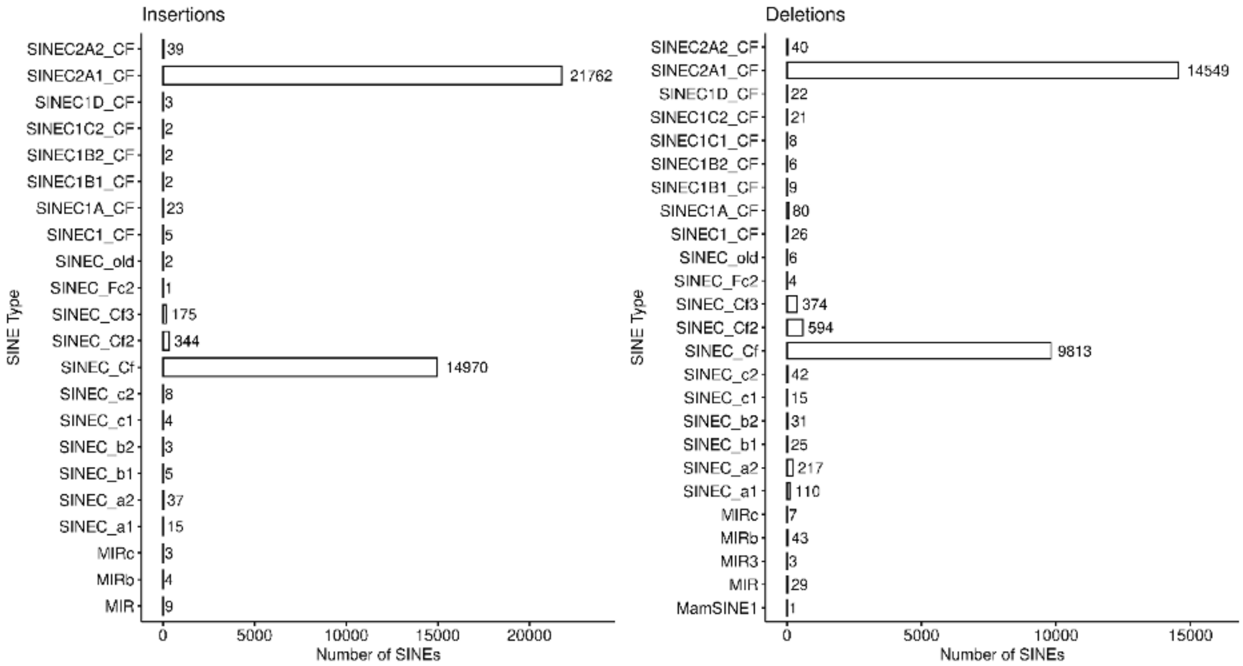

Fig. S3. Repeatmasker classification of SINE variation.

The sequence of structural variants with a size of 150-250 bp were analyzed using RepeatMasker and sequences classified as SINEs were identified. The total count of sequences from each subfamily are shown for insertions (left) and deletions (right) identified by Manta.

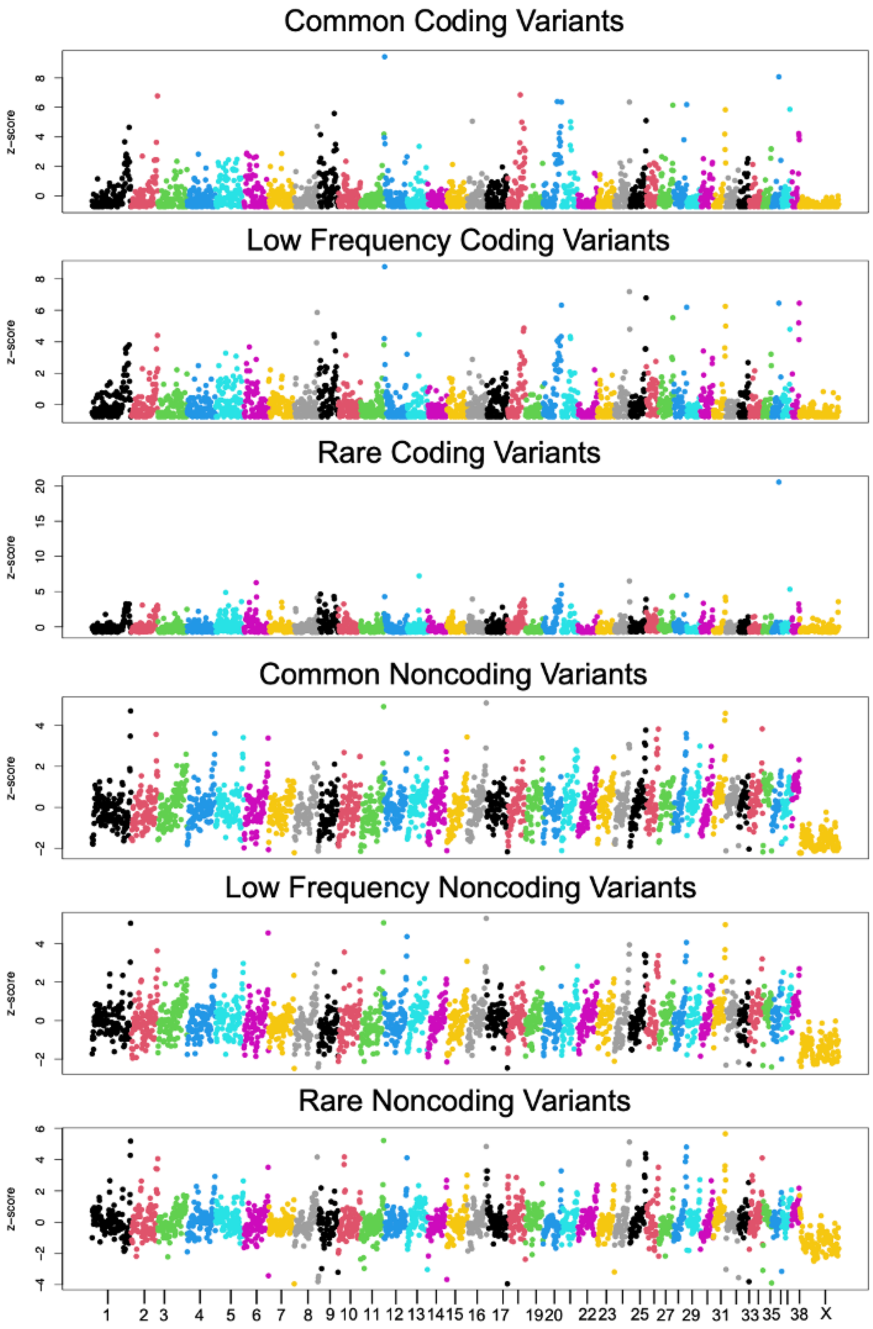

Fig. S4. Distribution of variation across the genome for breed and other dogs (n=1,591).
The genome was divided into 100kb bins, and SNV allele density calculated for breed dog and mixed/other categories (n=1,591), based on the genome region (coding or non-coding) and allele frequency bin (rare, AF≤1%; Intermediate, 1%<AF< 5%; common, AF≥5%). To aid visualization, the distribution was Z-transformed.

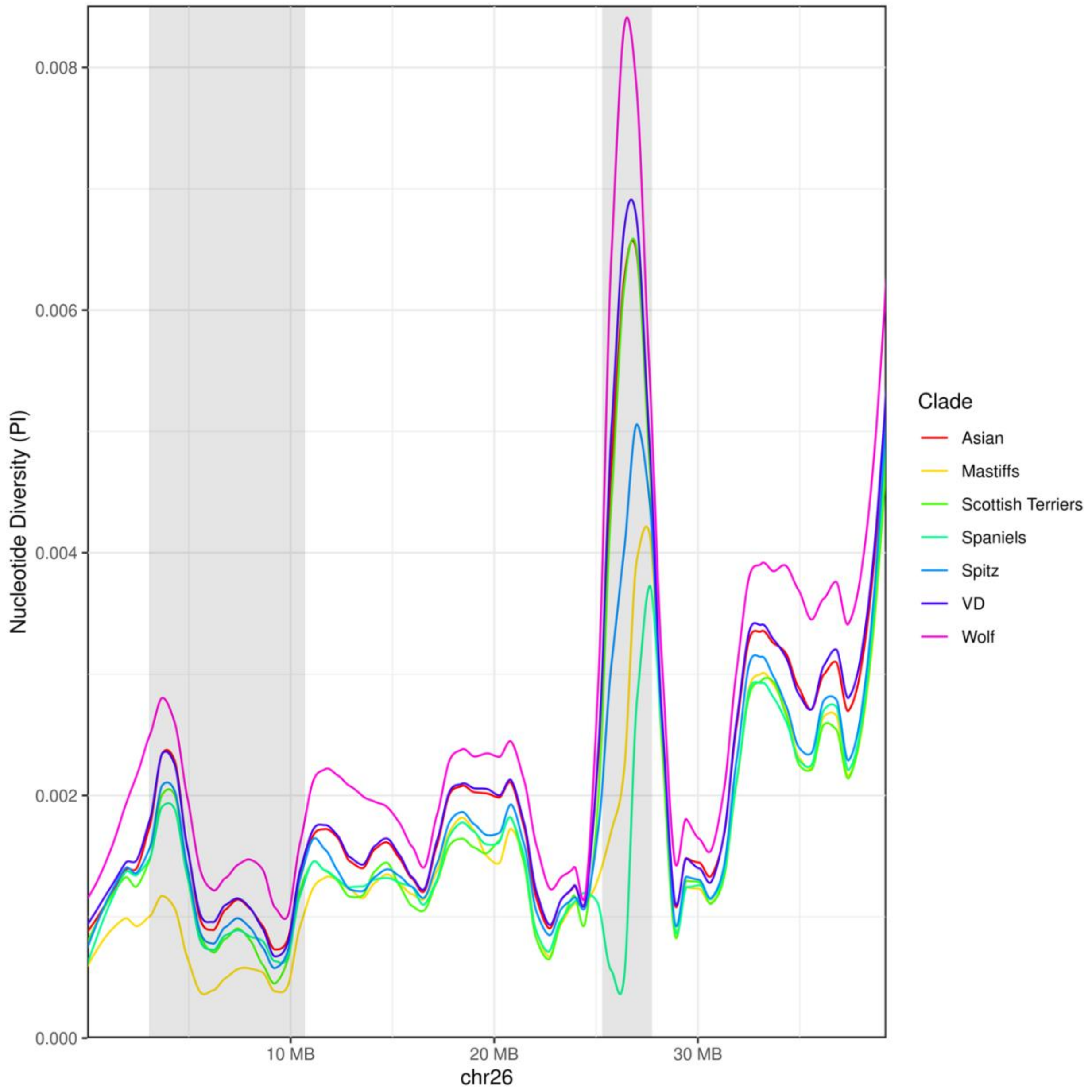

Fig. S5. Nucleotide diversity along chr26. Each line depicts observed nucleotide diversity (**π**) found in each analyzed clade along chr26. The gray box at the left delineates the region identified as under selection in Mastiffs using the program Ohana. The gray box at the right depicts a region of increased copy-number identified in the Dog10K data. The increase in nucleotide diversity likely reflects miscalled variants due to the copy number change.

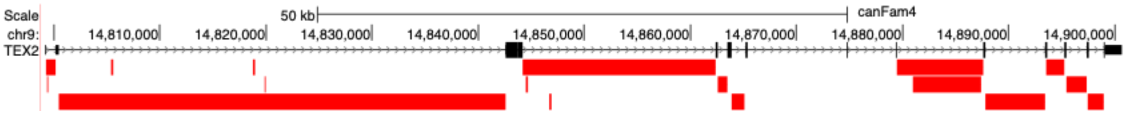

Fig. S6. Signature of a retrogene detected at the *TEX2* locus.

A UCSC browser view of the *TEX2* locus on chr9 is shown. The gene structure is depicted by black rectangles (exons) connected by a black horizontal line (introns). Plotted below in red are the positions of deletion variants detected by Manta. Ten of the *TEX2* introns have a 99% reciprocal overlap with a Manta deletion, suggesting the presence of a *TEX2* retrogene in the analyzed samples.

INDEX OF SUPPLEMENTARY TABLES

Table S1. Sample metadata and analyses where used.
Table S2. Samples with high F2 allele sharing with wolves.
Table S3. Variation yet to be discovered based on 100 samples per breed.
Table S4. Breed group placement of each sample.
Table S5. Runs of homozygosity (ROH) statistics per sample.
Table S6. Publicly available test samples used to measure imputation accuracy.
Table S7. Copy number variable genes.
Table S8. Potential retrogenes.
Table S9. Candidate genes either overlapping or within a 100kb distance of a significant site for each targeted ancestry.
Table S10. iSAFE top 10 sites per selection signature and ancestry component
Table S11. Observed fraction of theoretically possible SNVs.
Table S12. Alignment and filtering strategies for three panels of normal variation.
Table S13. Breed categories included in three panels of normal variation.
Table S14. Summary of variant counts in Dog10K sample sets.
Table S15. Distribution of SNVs across functional classes and Dog10K sample sets.
Table S16 Genotypes observed for 76 OMIA variants.
Table S17. Allele frequency distributions detected in Dog10K for OMIA categories.
Table S18. Variant positions deviating from HWE.
Table S19. Animal protocols, approving board, and date of approval.
Table S20. Summary of duplicate genes within NCBI release 106.
Table S21. Samples used in Ohana selection analysis.

Supplementary Tables are available in Additional file 1: Supplementary Tables.

SUPPLEMENTARY METHODS

Section 1: Reference genome construction and sample processing

By: Jennifer R. S. Meadows, Vidhya Jagannathan, Anthony Nguyen, Chao Wang, Jeffrey M. Kidd

Samples available for processing

Dog10K collected 2,075 samples under the categories of registered breed (1,649), mixed or other (18), village dogs (336), wolves (68) and coyotes (4). Metadata, including sample category, sex, WGS coverage, sampling location and sample provider is included in Additional file 1: Table S1.

Genome assembly used for read alignment

The Dog10K consortium utilized a modified version of the Wang et al. German Shepherd genome assembly for variant calling [30]. Specifically, the UU_Cfam_GSD_1.0 genome assembly was downloaded from the UCSC Genome Browser (CanFam4, GCA_011100685.1). UCSC naming conventions were utilized and the three Y chromosome sequences from the Labrador Retriever (ROS_Cfam_1.0, accession GCF_014441545.1) genome assembly were included. The Y chromosome sequences were included to reduce the effect of erroneous read placement for male samples. We refer to this assembly as 'UU_Cfam_GSD_1.0_ROSY'. The files used for alignment are indicated in *inline table 1.1* and are available at https://kiddlabshare.med.umich.edu/public-data/UU_Cfam_GSD_1.0-Y/ and at https://zenodo.org/record/8084059 [158].

Inline Table 1.1. Additional Y chromosome sequences included in the genome used for alignment.

Sequence Name	Accession
chrY_NC_051844.1	NW_024010443.1
chrY_unplaced_NW_024010443.1	NW_024010443.1
chrY_unplaced_NW_024010444.1	NW_024010444.1

Identification of the pseudoautosomal region

The pseudoautosomal region (PAR) is a segment of homology between the X and Y chromosomes in mammals. During male meiosis, recombination between the X and Y chromosomes occurs in the PAR. The PAR behaves like an autosomal locus and is diploid in males. Homology between the X and Y chromosomes ends in a region known as the pseudoautosomal region boundary, which separates the PAR from sequence specific to each sex chromosome [165]. Cytogenetic and sequencing approaches have localized the canine PAR boundary to the 3' end of the *SHROOM2* gene [166, 167], identifying a PAR approximately 6.5 Mb in length.

To identify the approximate PAR boundary, we constructed read-depth profiles using Illumina whole genome sequencing data from seven male and six female dogs (*inline table 1.2*).

Inline table 1.2. Samples used for PAR identification.

Sample Name	Sample Sex	Read Accession
CH019	Male	SRR7107579
CH027	Female	ERR2750983
DS064	Male	ERR2113150
DS077	Female	ERR3339004
FB081	Female	ERR3687180
GW004	Female	SRR7107585
LB214	Male	ERR2759446
LE2450	Female	ERR2357313
MA437	Male	ERR2750973
MA446	Male	ERR3687200
WE008	Male	ERR2113151
WH083	Male	ERR1688111
WH118	Female	ERR3339005

Coverage profiles were tabulated in 1,000 bp windows using Mosdepth [168]. Inspection of read depth profiles identified the approximate PAR boundary as position 6,605,250 on the X chromosome (*Inline figure 1.1*).

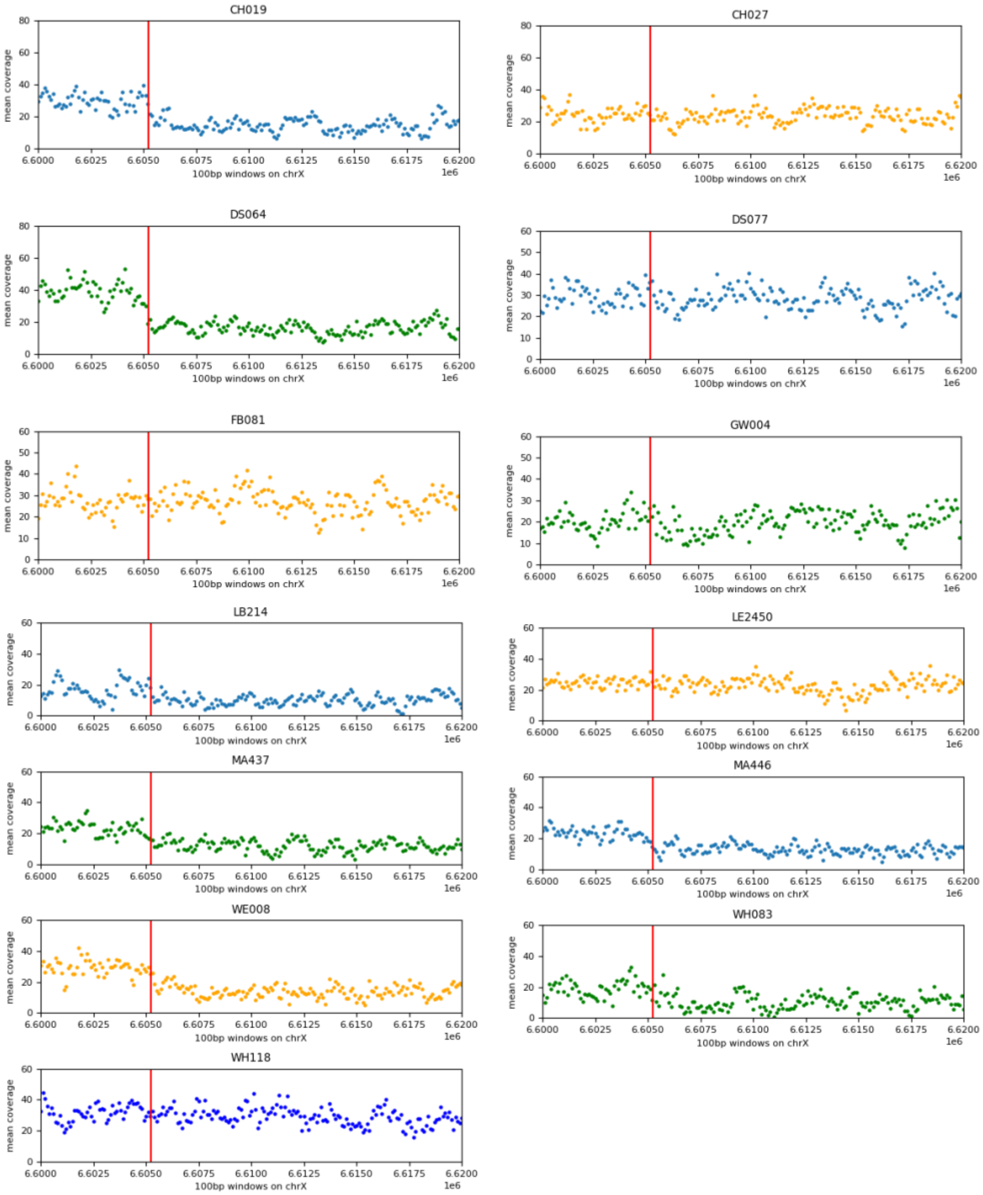

Inline Figure 1.1. Defining the pseudoautosomal region (PAR) on the canine X chromosome. The PAR has been operationally defined based on the read depth profile in male samples. The red line marks the approximate boundary of the PAR at 6,605,250 bp of the X chromosome.

Read alignment

Read alignment and processing was performed at multiple sites using a standardized pipeline inspired by Regier et al. [37]. Reads were aligned using bwa-mem2 version 2.1 against a modified version of the German Shepherd genome assembly described above using the command bwa-mem2 mem -K 100000000 -t NUM_THREADS -Y [39]. Alignment sorting, duplicate marking, quality recalibration, and variant calling was performed using Genome Analysis Tool Kit version 4.2.0.0 [38, 169]. More than 58 million SNV and 7 million indel positions identified based on alignment of existing canine data to canFam3.1 were

converted to UU_Cfam_GSD_1.0 coordinates and used to perform base quality scores recalibration (BQSR) [4, 117].

To reduce file size, base quality scores were quantized using the options --preserve-qscores-less-than 6 --static-quantized-quals 10 --static-quantized-quals 20 --static-quantized-quals 30 and converted to CRAM format using the GATK PrintReads function. Effective coverage statistics were calculated based on coverage at sites included on the Illumina CanineHD BeadChip genotyping array. Scripts and files used for recalibration in this pipeline are available at https://github.com/jmkidd/dogmap [159]. An archival version is available under the MIT Open Access License at https://zenodo.org/record/8087879 [162].

SNVs were discovered and genotyped using the GATK Haplotype Caller. Variant selection was performed using the variant quality score recalibration (VQSR) approach using the union of sites on the Illumina CanineHD BeadChip and Axiom K9 HD genotyping arrays as known sites (602,478 autosomal and 10,680 X chromosome variants). Candidate SNVs were selected based on criteria that included 99.0% of variants at the known training sites. Genotyping was performed separately for the autosomes and PAR region of the X chromosome and for the non-PAR region of the X chromosome. The ploidy of males was set to one for the non-PAR region of the X chromosome.

Samples were aligned at four different centers. Aligned files were gathered at the University of Michigan and subject to data quality control and variant calling. Results of processing of common test files were compared prior to initialization of alignment. A brief description of the alignment process used at each center is given below.

Institute of Genetics, University of Bern, Switzerland (Bern), 259 samples. Samples were processed using the stages described in the dogmap pipeline implemented in a Nextflow [170] pipeline script.

Department of Veterinary Biosciences, University of Helsinki, Finland (Helsinki), 605 samples. Samples were processed using the dogmap pipeline as described above.

Department of Human Genetics, University of Michigan, United States (Michigan), 869 samples. Samples were processed using the dogmap pipeline as described above.

Department of Medical Biochemistry and Microbiology, *Uppsala University*, *Sweden (Uppsala)*, 342 samples. Samples were processed using the main parameters of the dogmap pipeline as described, using a script adapted to the Slurm system in UPPMAX. After read mapping, the alignment was sorted and indexed using SAMtools v1.14 [152] The alignment was then split into 10 similar-size segments by SAMtools to parallelize the process. For each segment, the duplicated reads were marked using MarkDuplicates in Picard tools v2.23.4 (http://broadinstitute.github.io/picard), and BQSR were assessed using BaseRecalibrator (GATK, v4.2.0.0). BQSR segment reports were merged with GatherBQSRReports (GATK) and recalibration was applied. Afterward, the alignment segments were merged, sorted and indexed using SAMtools. The script is available at https://github.com/Chao912/dog_10k/ [171] and an archived version is available at https://zenodo.org/record/8087147 [172].

Sequence quality analysis

Several analyses were performed to assess data quality prior to performing variant discovery. First, a preliminary review of sample meta-data was performed and two samples, GRIF000001 and GRIF000002, were removed because a precise breed identification was not available. The mean coverage reported by the GATK HaplotypeCaller at 146,029 autosomal SNV positions available from the Illumina CanineHD genotyping array was determined for each sample. A total of 21 samples with a mean effective autosomal coverage less than 10x were removed from further analysis (*inline figure 1.2*).

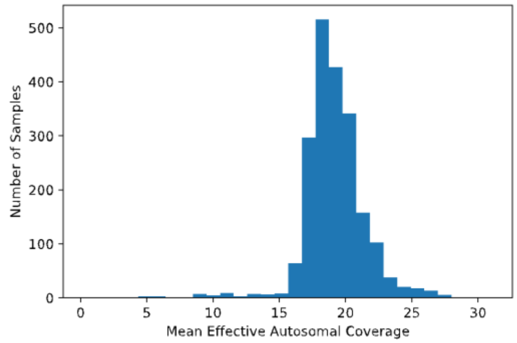

Inline figure 1.2. Mean effective autosomal coverage for 2,073 aligned samples. Samples with a coverage less than 10x were removed from analysis.

Next, we assessed possible contamination by examining the fraction of reads with the reference allele at heterozygous SNP positions. Analysis was limited to autosomal SNPs included on the Illumina CanineHD genotyping array with a heterozygous genotype reported by the GATK HaplotypeCaller. The reference allele is expected to be present in 50% of aligned reads at heterozygous positions, with slight deviations due to random sampling and mapping effects. Contamination skews the read fraction away from 50% (*inline figure 1.3*).

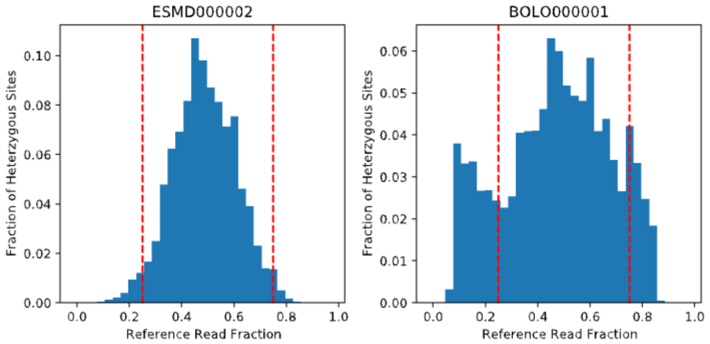

Inline figure 1.3. Reference read fraction at heterozygous sites. The reference allele fraction distribution is shown for two samples. ESMD000002 shows the expected profile whereas BOLO000001 has a reference read fraction indicative of potential contamination. We quantified the deviation in reference read fraction based on the fraction of heterozygous sites with a read fraction ≤ 25% or ≥ 75% (red dashed lines). Sample ESMD000002 has 4.6% of sites with read fractions in these intervals while 29.6% of sites in BOLO000001 have a read fraction in these intervals.

We measured this for each sample by calculating the fraction of heterozygous sites with a reference read fraction ≤ 25% or ≥ 75%, a value we refer to as the read balance tail fraction. The median read balance tail fraction was 0.052, with a subset of samples having a greatly increased read balance score. We identified 50 samples as potentially contaminated using a cutoff of 0.1004, corresponding to seven median absolute deviations above the median (*inline figure 1.4*).

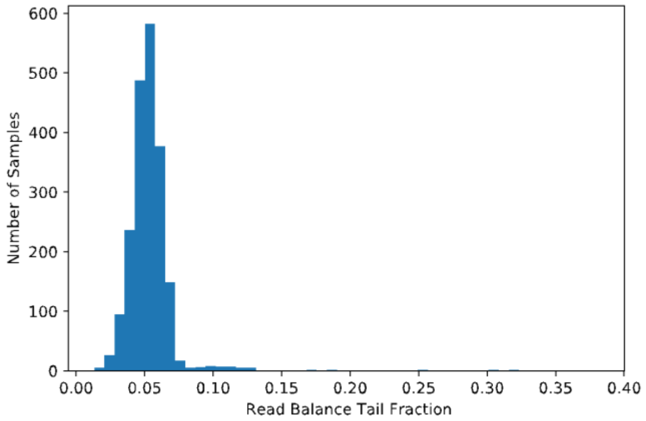

*Inline figure 1.4. Distribution of read balance tail fraction scores for 2,052 samples***.** We identified 50 samples with a tail fraction greater than 0.1004 as potentially contaminated.

Consistent with the conclusion that these 50 samples are contaminated, we found that the filtered samples include individuals that have an intermediate X vs Autosome Depth ratio or that have a large number of heterozygous genotypes on the X chromosome despite having a depth profile consistent with the presence of one X chromosome (*inline figure 1.5*).

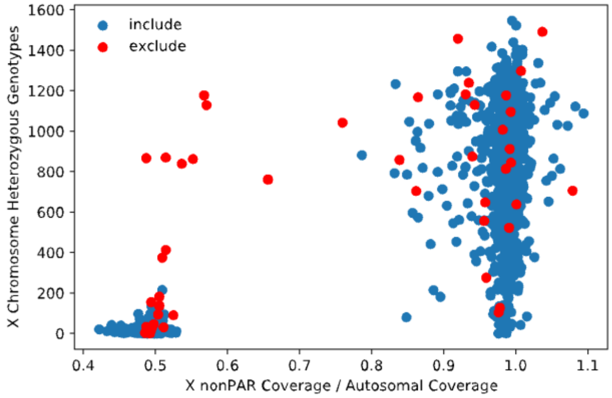

Inline figure 1.5. Filtered samples show additional signatures of contamination. A scatter plot of the X vs autosome read depth ratio (X axis) vs the number of heterozygous genotypes at SNV positions on the X chromosome (Y axis) is shown. The 50 samples identified as potentially contaminated based on reference read allele fraction are plotted in red. Samples with an intermediate X vs Autosome coverage level as well as male samples with an excess of heterozygous genotypes on the X chromosome were marked for removal by the read balance filter.

Identification of duplicate and mislabeled samples

We used PLINK v 1.9 [134] to calculate the pairwise identity by state (IBS) matrix for the remaining 2,052 samples based on inferred genotypes at 138,333 autosomal SNVs available from the Illumina CanineHD genotyping array that have a minor allele frequency greater than 0.01 and a genotype missingness less than 0.1. We identified 25 sample pairs with an IBS value greater than 0.9. This includes 7 pairs with an IBS value greater than 0.99 that were clear outliers relative to the other individuals of the same breed or group (*inline figure 1.6*).

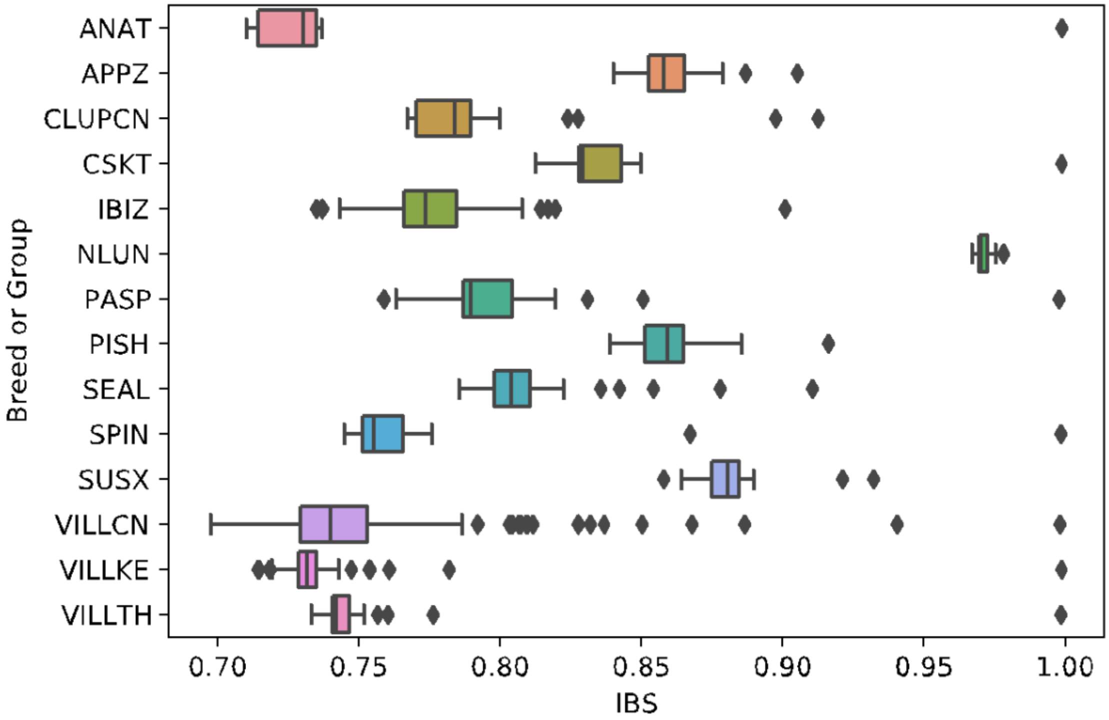

Inline Figure 1.6. Boxplots of pairwise IBS values for outlier samples. IBS analysis identified 25 sample pairs with an IBS value greater than 0.9. Boxplots of pairwise IBS values are shown for each of the 14 breeds or groups represented by the 25 samples. Seven pairs have an IBS value clearly greater than the other values observed within the breed or group. ANAT: Anatolian Shepherd Dog, APPZ: Appenzeller Sennenhund, CLUPCN: Wolf, China, CSKT: Cesky Terrier, IBIZ: Ibizan Hound, NLUN: Norwegian Lundehund, PASP: Pont-Audemer Spaniel, PISH: Picardy Shepherd, SEAL: Sealyham Terrier, SPIN: Spinone Italiano, SUSX: Sussex Spaniel, VILLKE: Village Dog, Kenya, VILLTH: Village Dog, Thailand

We considered these 7 samples to be duplicates, resulting either from the repeated collection of material from the same animal or from labeling and processing errors, and removed the individual with the lowest sequencing depth in each pair from further analysis.

We also searched for breed dogs with an incorrect breed assignment. For each sample we calculated the mean pairwise IBS value to all other samples of the same breed and compared the value to the mean pairwise IBS value found among the other samples in that breed. This analysis was limited to breeds that include four or more individuals. We identified 18 breed dogs that have a within-breed IBS at least 4 standard deviations lower than the other dogs belonging to that breed. We identified the sample with the

highest IBS score to each of the 18 outliers and found that the most similar samples represented a clear match to an unrelated breed for 8 of the samples (*inline table 1.3*). We considered these 8 samples to be mislabeled and removed them from analysis.

Inline Table 1.3. Identification of samples with incorrect breed assignment.

Sample	Mean IBS vs Group	Mean Group IBS	Z	Closest Sample	IBS	Conclusion
DEER000004	0.6830	0.8309	-56.7	WSSD000005	0.7212	Mislabeled
CHIN000008	0.6908	0.8033	-20.2	SARP000003	0.7605	Mislabeled
LAGO000003	0.7028	0.7770	-15.3	BEAU000003	0.7747	Mislabeled
TIBS000002	0.6990	0.7828	-13.4	KARS000001	0.7411	Mislabeled
IWSP000004	0.7891	0.8340	-12.8	IWSP000002	0.7964	
GOLD000001	0.7461	0.8131	-10.5	GOLD000005	0.7512	
KROM000005	0.7709	0.8418	-7.5	KROM000007	0.7753	
AUCD000001	0.7519	0.7756	-7.0	AUCD000003	0.7551	
AMST000004	0.7175	0.7693	-6.7	STAF000004	0.8016	Mislabeled
COTO000002	0.7015	0.7400	-6.4	PYMF000003	0.7725	Mislabeled
HOVA000001	0.7929	0.8016	-5.8	HOVA000004	0.7983	
KEES000001	0.6800	0.7600	-5.0	CHOW000003	0.7842	Mislabeled
AUPO000005	0.7117	0.7938	-4.9	BOLO000001	0.7389	
BRIA000007	0.7008	0.7821	-4.7	GRSD000001	0.7757	Mislabeled
PBGA000002	0.7465	0.7610	-4.6	PBGA000001	0.7566	
BLDH000002	0.8298	0.8395	-4.5	BLDH000004	0.8336	
PRKR000005	0.7420	0.7513	-4.1	PRKR000001	0.7496	
CAUC000001	0.7270	0.7367	-4.0	KARS000001	0.7515	

Results are shown for the 18 breed dogs with a within-breed mean IBS value that is at least 4 standard deviations below the average found for other samples of the same breed. The sample with the greatest similarity to each index sample is also shown.

In total, 1,987 samples passed the quality assessments and were included in the joint genotyping and subsequent analyses (*inline table 1.4*). Although the metrics described above identified several problematic samples, we note that they have several limitations. First, the breed assignment analysis is limited to breed dogs. Additionally, we limited the comparison to the 2,052 samples with at least 10x sequence coverage;

existing collections of samples that have been genotyped on the Illumina SNP array were not utilized. Finally, the SNP array positions, which have a skewed ascertainment, may not provide sufficient resolution to properly discriminate among sample relationships for all breeds. As described in Supplementary Methods Section 9 (Additional variant filtration for functional analysis), additional samples were removed from analysis based on examination of the whole genome SNV genotyping results.

Inline table 1.4. Summary of samples removed based on quality analysis

**Initial samples**	**2,075**
Precise breed unknown	2
Less than 10X coverage	21
Skewed reference read fraction	50
Sample duplicates	7
Mislabeled breed dogs	8
**Total remaining samples**	**1,987**

Section. 2 Genome wide identification of SNVs and indels

By: Jeffrey M. Kidd

Variant calling

SNV and indel calls were generated from 1,987 samples that passed the initial data quality checks described in Supplementary Methods Section 1. GVCF files were combined using the GATK GenomicsDBImport function and processed using the GenotypeGVCFs function. The genome was divided into 1 Mb segments that overlapped by 2 kb and processed in parallel. SNV and non-SNV variants were separated using the GATK SelectVariants function and processed separately. For SNVs, the VariantRecalibrator function was applied separately to data from the autosomes and X PAR region and the non-PAR region of the X chromosome. The combined set of available variants from the Illumina CanineHD BeadChip and Axiom K9 HD genotyping arrays were used as known sites with options -resource:array,known =false, training=true,truth=true,prior=12.0 SRZ189891_722g.simp.header.CanineHDandAxiom _K9_HD.GSD_1.0.vcf.gz --use-annotation QD --use-annotation MQ --use-annotation MQRankSum --use-annotation ReadPosRankSum --use-annotation FS --use-annotation SOR --use-annotation DP --trust-all-polymorphic true -mode SNP -tranche 100.0 -tranche 99.9 -tranche 99.0 -tranche 98.0 -tranche 97.0 -tranche 96.0 -tranche 95.0 -tranche 94.0 -tranche 93.0 -tranche 92.0 -tranche 91.0 -tranche 90.0. Due to the reduced training data size, the option --max-gaussians 4 was used for the X non-PAR variants. ApplyVQSR was then used to select the 99.0% tranche of variants. This resulted in a total of 33,374,690 SNPs on the autosomes+X-PAR and 1,191,860 SNPs on the X-nonPAR.

Due to the absence of high-quality training data, a hard filter was used to select high-quality candidate indel variants. The GATK VariantFiltration tool was used with the following options: -filter "QD < 2.0" --filter-name "QD2" -filter "FS > 200.0" --filter-name "FS200" -filter "ReadPosRankSum < -2.0" --filter-name "ReadPosRankSum-2" -filter "SOR > 10.0" --filter-name "SOR-10". A total of 14,414,501 indels passed these filters.

When tabulated in 50 kb windows among the autosomes, the last megabase of each chromosome has a mean density of 24.3 SNVs per kbp, compared to the 14.7 SNVs per kbp observed elsewhere, an approximately 65% increase ($p<10^{-30}$, Welch's unequal variances *t*-test; *inline figure 2.1*).

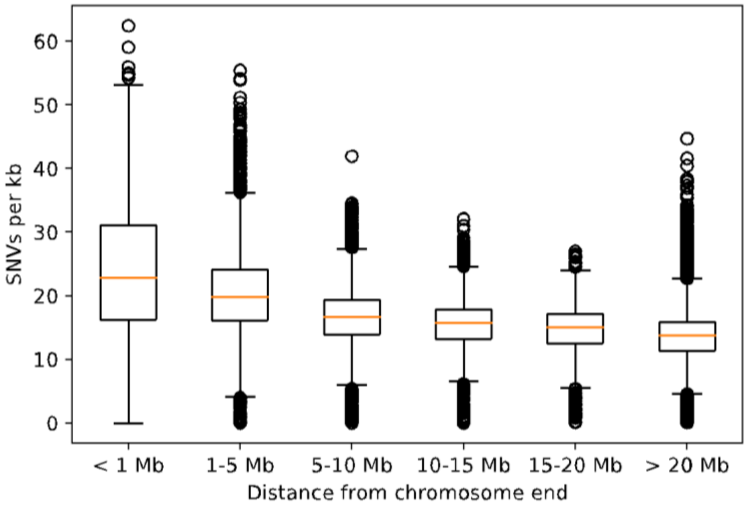

Inline figure 2.1. Increased SNV density near chromosome ends. Boxplots of SNV density in 50 kbp windows among autosomes, broken down by distance to chromosome end, is shown.

A clear correlation with GC content in 50 kb windows is also observed (Pearson's r = 0.372; *inline figure 2.2*).

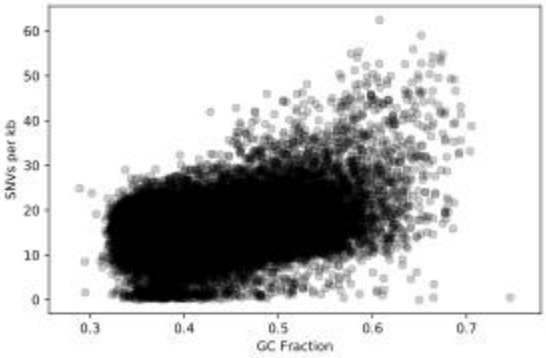

Inline figure 2.2. SNV density is correlated with GC content. A scatter plot of SNV density versus GC sequence fraction is shown in 50 kb windows for the autosomes. The two values have a Pearson correlation of r= 0.372.

Estimation of callable genome locations

Mapping properties from large scale alignments can be used to identify regions of the genome that are amenable to analysis using short-read sequencing data [42]. Such a mask may be useful for some down-stream genome analyses. Regions of the genome that are accessible by short read sequencing were identified from the alignment of 1,987 samples. Positions that are 'N' in the genome reference, positions where ≥ 10% of aligned reads have a mapping quality (MQ) of 0, and regions where the total coverage was more than 50% away from the median coverage were identified as failing the callability mask (*inline table 2.1*). Cutoffs were determined separately for the autosomes and X-PAR region and the non-PAR segment of the X chromosome. A total of 2,259,749,455 positions passed the above criteria, representing 96% of the 2,353,510,098 non-N positions in the assembly.

Inline table 2.1. Median total coverage and cutoffs used to construct callable genome mask.

**Region**	**Median**	**Low Cutoff**	**High Cutoff**
Autosomes + X PAR	36,634	18,317	54,951
non-PAR X	27,621	13,810.5	41,431.5

Section 3 Breed relationships and haplotype sharing

By: Heidi Parker

Breed cladogram

The placement of samples relative to each other was assessed and individuals were removed from the breed analysis if they 1) did not cluster with the multi-breed clade that contained all other member of the same breed; 2) they were listed as an ambiguous breed; or 3) they were part of a population that included first generation hybrids. In a preliminary analysis, sixteen individuals were removed due to unresolved mislabeling or sample mix-ups because they were placed outside of the multi-breed clade where all other sequences from the same breed were clustered. An additional 22 samples from ambiguous breeds or populations that include first generation mixes were excluded from breed analyses. The two Mountain Curr samples were omitted from breed analysis because the Mountain Curr breed is not yet fully established. Additionally, 12 samples that were assigned a category of Mixed/Other since they are of mixed origin or are not part of genetically defined breeds. These include samples labeled as Anglo-Francais Hound, Bandog, German Shepherd Mix, Carolina Dog, and Pit Bull Terrier; each was omitted from the breed phylogeny analysis. All other sequences cluster with individuals of the same breed or a closely related breed. For the 31 breeds represented by only 1 sample, the placement of 14 were confirmed by down-sampling the variants to those included on the Illumina Canine HD SNP bead array and clustering the individuals with previously published SNV genotype data [49]. Using both random subsampling and bootstrapping, the average branch retention value for the full tree was >98% (98.4 and 98.5 respectively).

D-statistics

D-stats were calculated for German Shepherd-like breeds to assess wolf admixture. The R package *admixr* [137] was used to run Admixtools v7.0.2 [138] on the tree structure (W, X)(Y, Z) where W=German Shepherd dog, Z = Coyote, X = the list of German Shepherd related breeds, and Y = the list of wolf populations. Significance was set at $|Z| \geq 3$ (*inline figure 3.1*).

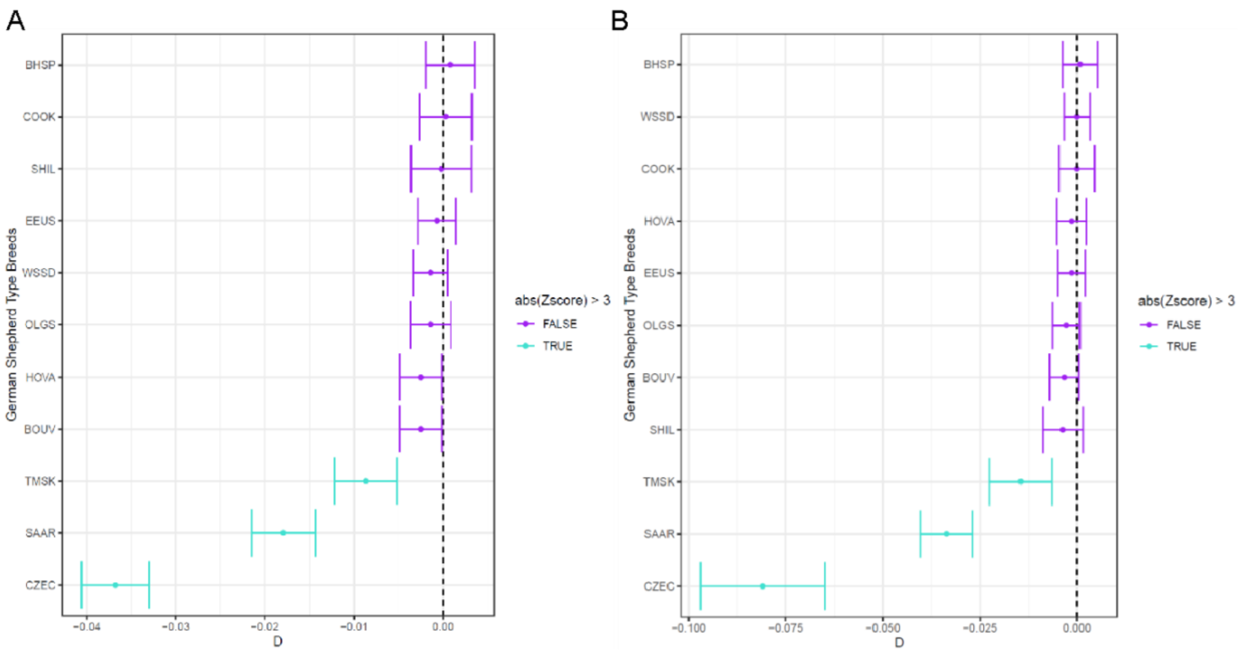

Inline figure 3.1. D statistic analysis of excess wolf allele sharing among Shepherd-like breeds. D-statistics of the form (German Shepherd, Shepherd-related breed X, Wolf population Y, Coyote) were calculated using Russian Wolf (panel A) or Eurasian Wolf (CLUPEA000001, panel B). The Tamaskan, Saarloos Wolfdog, and Czechoslovakian Wolfdog show excess sharing with wolves relative to German Shepherds. Breed abbreviations: BHSP Bohemian Shepherd, COOK Chinook, SHIL Shiloh Shepherd, EEUS East-European shepherd, WSSD White Swiss Shepherd Dog, OLGS Old German Shepherd, HOVA Hovawart, BOUV Bouvier des Flandres, TMSK Tamaskan, SAAR Saarloos Wolfdog, CZEC Czechoslovakian Wolfdog. Significance is set at $|Z| \geq 3$.

Compared to German Shepherds, three breeds showed significant wolf admixture with the majority of wolf populations: the Czechoslovakian Wolfdog (12/12 wolf populations), the Saarloos Wolfdog (10/12 wolf populations) and the Tamaskan (9/12 wolf populations). These three breeds, along with one other, the Shilo Shepherd, include at least one wolf or wolf-hybrid in their reported historical ancestral pool. Only the Czechoslovakian Wolfdog and Saarloos Wolfdog display haplotype sharing at above median breed to breed sharing levels with European wolves (maximum of 76.6 Mb and 23.8 Mb with the Eurasian wolf, respectively), suggesting recent wolf admixture [49]. The Shiloh Shepherd and Tamaskan show higher than average haplotype sharing with wolves (995 kb and 897 kb respectively, the average wolf to dog sharing is 298 kb). We note several potential limitations of this study including our use of a Coyote as an outgroup, since Coyote-Wolf admixture [173-175] may skew the results, and our focus on breed samples represented in the Dog10K collection. An extended analysis that includes a more diverged outgroup, a broader selection of breeds, and information from ancient DNA is required for a comprehensive assessment of historic gene flow between breed dogs and wolves.

Section 4 Variation among breeds

By: Jeffrey M. Kidd

Allele sharing

The samples analyzed in this section are noted in the *inline table 4.1* and include 321 breeds with 261 breeds represented by three or more individuals (*inline figure 4.1*). The 281 Village Dog samples were from 26 different countries and included additional geographic subdivisions. The 57 wolf samples were from across Eurasia.

Inline table 4.1. Sample types for the 1,929 samples that pass final SNV QC Metrics.

**Sample Category**	**Number of Samples**
Breed Dogs	1579
Mixed/Other	12
Village Dogs	281
Wolf	57
**Total**	**1929**

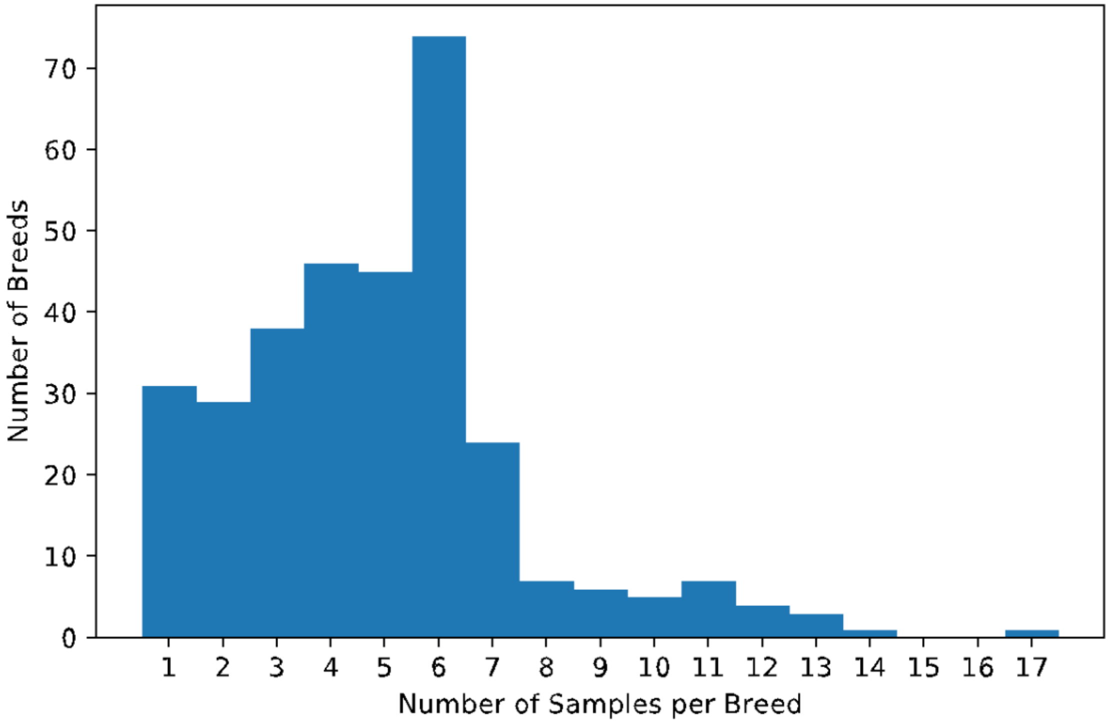

Inline figure 4.1. Number of samples per breed for 1,579 breed dogs that pass final SNV QC metrics.

We measure the statistic F_2 in the 1,929 Dog10K samples [41, 42]. F_2 refers to variants found in only two samples regardless of their zygosity and it is inspired by the count of f_2 variants (or doubletons), i.e., those present exactly twice in a sample. These rare variants are informative about recent shared ancestry. Here,

we use a total of 2,550,520 autosomal F_2 sites, of which 2,384,354 have no missing genotypes. The results per category are plotted in the *inline figure 4.2*.

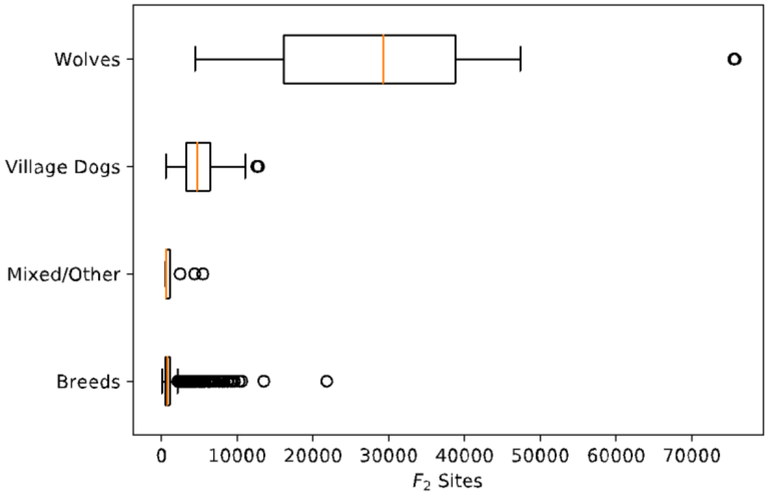

Inline figure 4.2. Number of F_2 sites per Dog10K category.

Estimation of variation to be discovered

We constructed the distribution of non-reference allele counts (the non-reference site frequency spectrum) for each sample group that has at least three individuals based on SNV sites with no missing genotypes within each sample group. We predicted the number of non-reference variants that would be discovered in a sample with 100 individuals by applying a linear program method to the observed site frequency spectrum [45]. The predicted fraction of variation already discovered varies widely among the 261 breeds that are represented by at least three individuals (*inline figure 4.3*). For example, we predict that our study of 5 Norwegian Lundehunds has captured 98.4% of the variation that would be discovered if 100 individuals of this breed were sequenced. For 22 breeds, we predict that we have already discovered more than 90% of the total predicted variants. For 20 breeds, we predict that 75% or less of the total variation has been discovered. This includes the Czechoslovakian Wolfdog, where we predict that the four analyzed samples only capture 47% of the variation expected in a sample of 100 individuals.

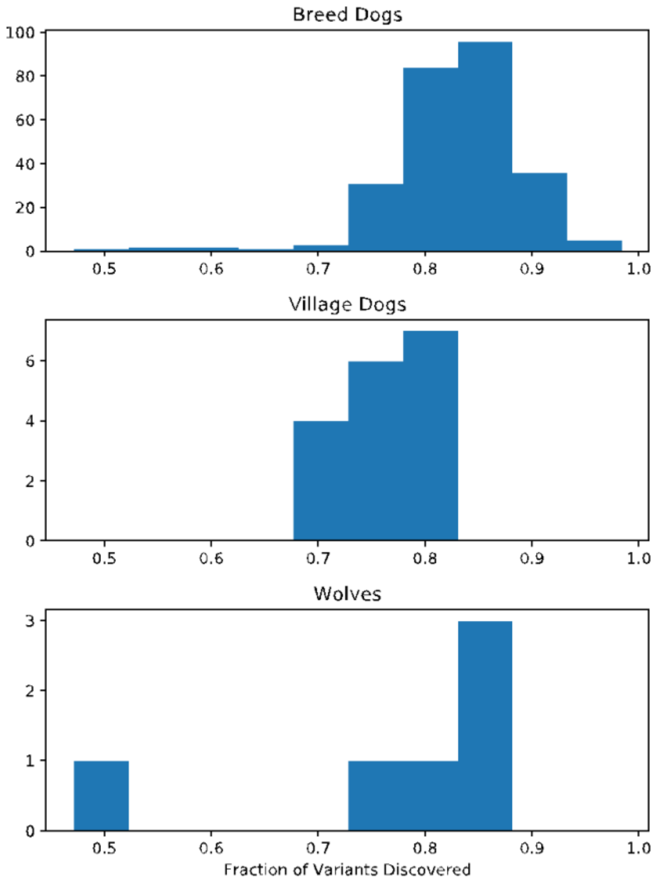

Inline figure 4.3. Fraction of SNVs expected in a sample of 100 individuals that is already found in the Dog10K Data. The predicted fraction of variants already discovered is shown for 259 breeds, 17 village dog groups, and 6 wolf groups that contain at least 3 individuals.

Section 5 Runs of homozygosity

By: Matthew Christmas

Identifying ROH regions

Input for this analysis was the set of autosomal 32,654,226 biallelic SNVs that passed the VQSR filtering. We identified runs of homozygosity (ROH) for all samples using the sliding-window approach implemented in PLINK v1.90b4.9 [176] with the'--homozyg' function and the following settings:

--homozyg-density 50
--homozyg-gap 1000
--homozyg-kb 200
--homozyg-snp 100
--homozyg-window-snp 100
--homozyg-window-threshold 0.05
--homozyg-window-het 3
--homozyg-window-missing 2

These settings were based on those previously recommended for high-density SNP datasets [139], with minimum average SNP density (--homozyg-density 50), maximum gap between adjacent SNPs (--homozyg-gap 1000), the size of the sliding window (--homozyg-kb 200), and the minimum number of variants needed to detect ROH (--homozyg-window-snp 100) set to reflect the average SNP density of the dataset. The '--homozyg-window-het' and '--homozyg-window-missing' flags were set to account for potential sequencing errors and missing data. The number of heterozygous sites to allow within a window (--homozyg-window-het 3) was set based on the average number of heterozygous sites called in male dogs outside of the pseudoautosomal regions on chromosome X (i.e., where all males are haploid and therefore any heterozygous calls are errors). This control data set was based on a separate SNV call set created by treating male samples as diploid for the entire X chromosome. We calculated the coefficient of inbreeding from our ROH estimates (F_{ROH}) by dividing the total length of all ROH within a sample by the genome size (i.e., F_{ROH} is the proportion of the genome within ROH). For a Dog10K category summary, see *inline figure 5.1*, followed by plots for Village dogs, wolves and coyotes and breeds (*inline figures 5.2, 5.3 and 5.4*).

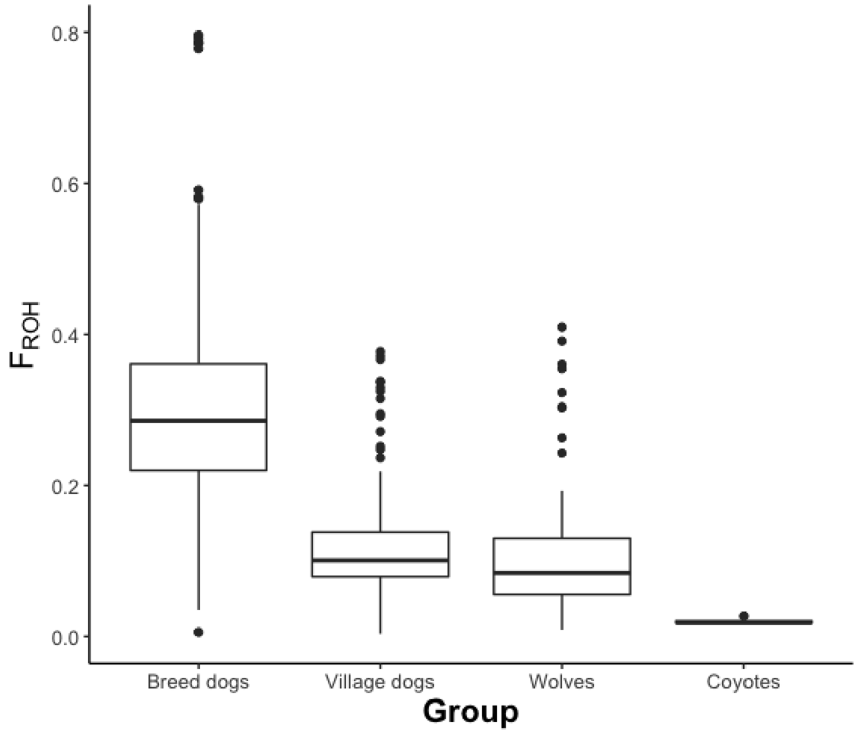

Inline figure 5.1. Box plots of F_{ROH} for each dog group and Coyotes.

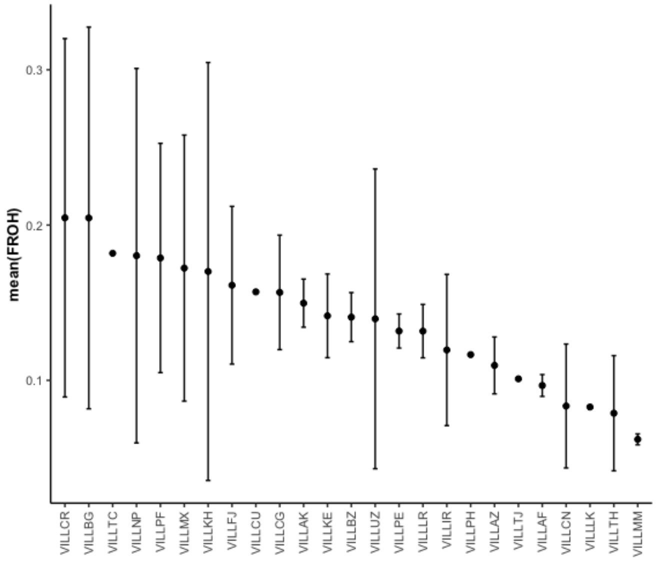

Inline figure 5.2. Mean (circles) and standard deviation (lines) F_{ROH} for all village dogs with at least 5 samples per population.

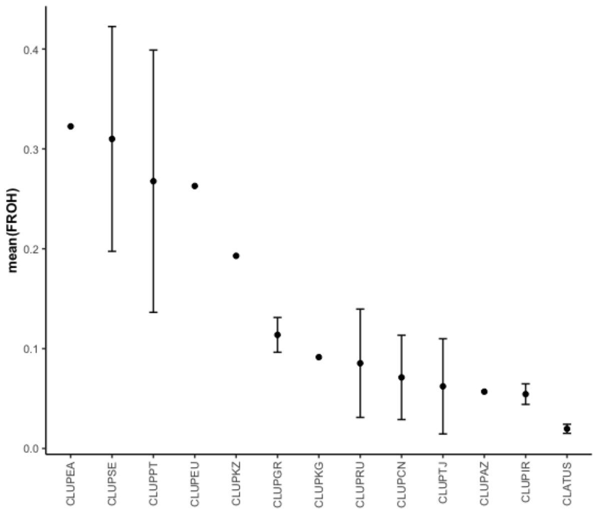

Inline figure 5.3. Mean (circles) and standard deviation (lines) F_{ROH} for all wolf and coyote (CLATUS) populations.

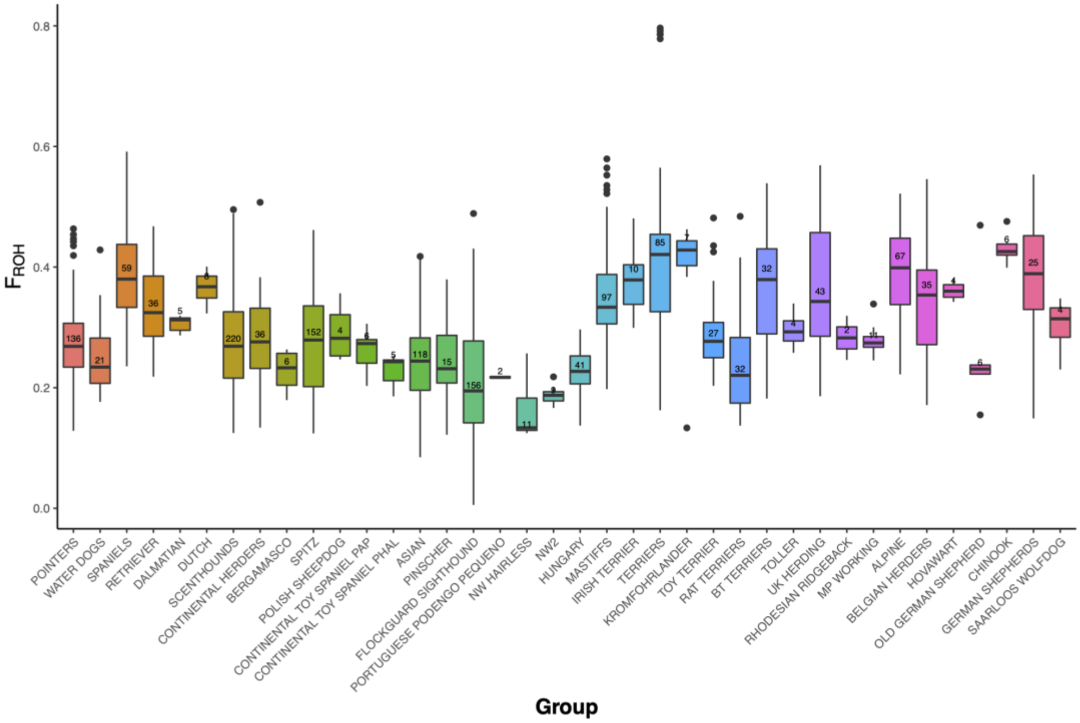

Inline figure 5.4. Box plots of F_{ROH} per breed group.

A correlation between the number of ROH segments and the summed length of the detected segments, as well as the ROH segments and their average length are shown for each breed below as scatter plots (*inline figures 5.5 and 5.6*).

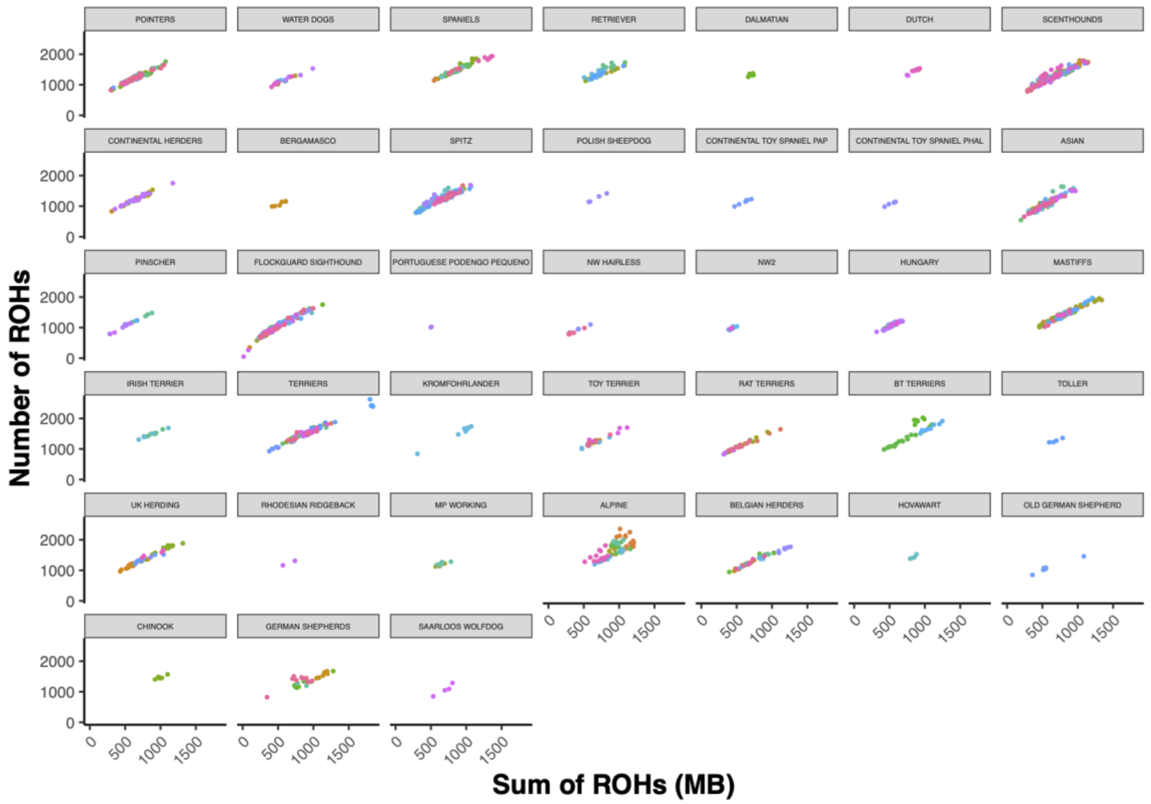

Inline figure 5.5. Correlation between total size of ROHs and number of ROH segments.

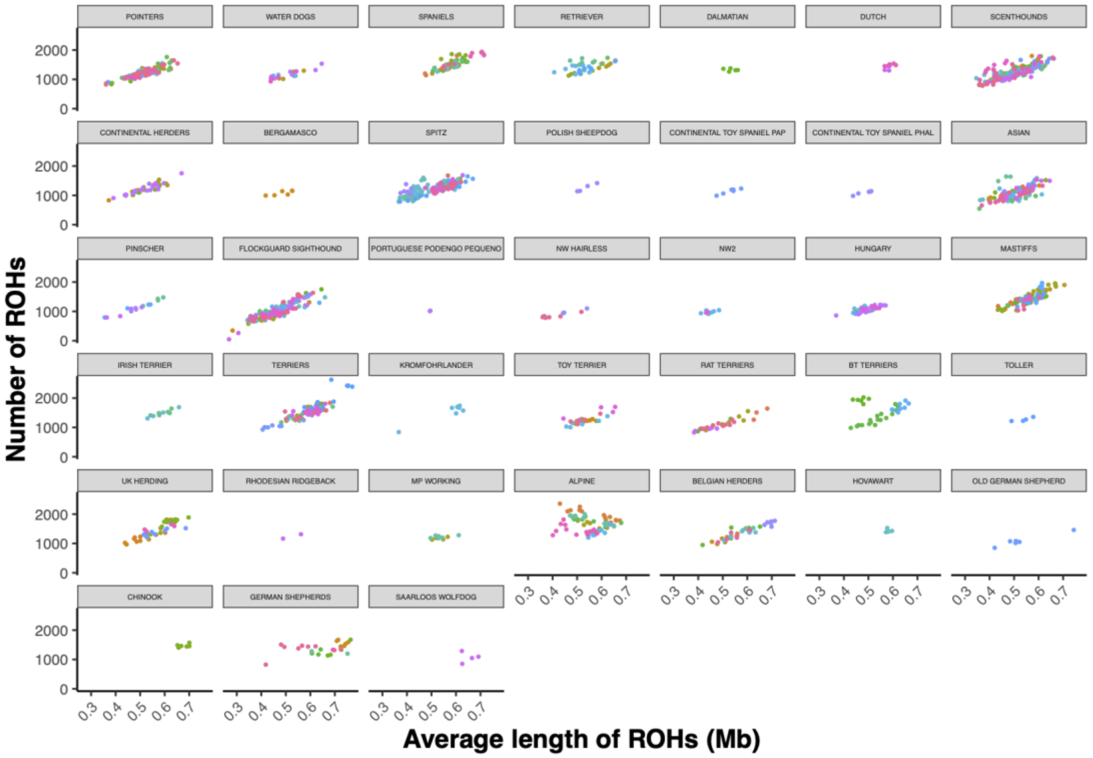

Inline figure 5.6. Correlation between the number of ROHs and their average length.

Section 6 Analysis of mitochondrial sequence

By: Greger Larson, Fabian Ramos-Almodovar, Jennifer R. S. Meadows, Peter Savolainen, Guo-Dong Wang, and Jeffrey M. Kidd

Identifying mitochondrial sequence variation using Illumina short-read sequencing presents several challenges due to the circular nature of the mitochondrial genome, extremely high read depths due to the high number of mitochondrial genomes per cell, the presence of heteroplasmies at variable allele fractions, and the misalignment of reads due to nuclear mitochondrial insertions. A pipeline that includes recent modifications to the Mutect2 variant caller has robustly identified mitochondrial variation in resequenced human genomes [145]. Here, we have adapted this pipeline to the analysis of mitochondrial sequence variation in canines.

Pipeline overview

Our procedure begins with reads aligned to the UU_Cfam_GSD_1.0_ROSY reference genome as previously described and gives final results relative to the NC_002008.4 canine reference mitochondrial genome [177]. First, Illumina read-pairs that have at least one read aligned to the UU_Cfam_GSD_1.0_ROSY chrM sequence or to a nuclear mitochondrial segment that is at least 300 bp long with at least 95% identity to the reference mitochondrial genome sequence (NC_002008.4) are extracted (*inline table 6.1*).

Inline table 6.1. Locations of nuclear mitochondrial insertions used for mitochondrial read recruitment.

**chromosome**	**start**	**end**	**identity**	**length**
chr10	15927342	15927681	95.28	339
chr20	10603520	10609165	97.42	5645
chr7	27683808	27684759	96.61	951
chrUn_JAAHUQ010000987v1	20706	22754	96.75	2048
chrUn_JAAHUQ010000987v1	1	17148	99.27	17147

Coordinates are given in BED format, sequence identity is calculated relative to NC_002008.4.

When the circular mitochondrial genome is linearized, a synthetic breakpoint is created at the beginning and end of the linear reference genome. This results in a lower rate of alignment for reads derived from the ends of the linear sequence. To compensate for this bias, an alternative mitochondrial genome that is linearized starting at position 8,000 was created. The extracted read-pairs are aligned to the NC_002008.4 reference mitochondrial genome as well as to the version of NC_002008.4 that has been rotated by 8 kb using bwa-MEM [146] version 0.7.15. This procedure compensates for the bias introduced by reads that align to the breakpoint used to create a linear representation of the circular mitochondrial genome.

Next, read depth is calculated using the CollectHsMetrics from GATK version 4.2.5.0. If the mean coverage is greater than 5,000, the resulting alignments are down-sampled to a depth of 5,000 using GATK DownsampleSam. Candidate variants are identified from each alignment using Mutect2 from GATK 4.2.5.0 with options --mitochondria-mode, --max-reads-per-alignment-start 75, --max-mnp-distance 0, and --annotation StrandBiasBySample. Filters are then applied to the resulting VCF file using GATK

FilterMutectCalls --mitochondria-mode. The VCF files generated from the original and rotated references are then merged, with variants in the first and last 4 kb taken from the alignment to the rotated reference. Sites where the most frequent alternative allele fails the strand_bias filter or represents a heteroplasmy (an allele fraction less than 0.5) are removed. A fasta representation of the mitochondrial sequence is then constructed using bcftools consensus (version 1.9). Regions with a coverage less than 100 and regions that overlap positions 15512-15535 or 15990-15990 are masked to 'N'. These regions correspond to a C-rich segment and a repetitive region that often contains heteroplasmic variation in the D-loop. It is hard or impossible to accurately identify variation in these regions and these regions are therefore normally masked out of mitochondrial analyses [178]. Note that this region is expanded relative to the segment described in Fregel et al. [178] as preliminary analysis identified artifacts in read alignments flanking the repeat. An implementation of this pipeline is available at https://github.com/jmkidd/callmito [161]. An archival version is available under the MIT Open Access at https://zenodo.org/record/8087897 [164].

Pipeline validation

To assess the accuracy of the mitochondrial variation discovery pipeline we compared the mitochondrial sequence constructed from Illumina data with that reported in five recently published long-read canine genome assemblies [29-32, 35]. Illumina reads from each sample were aligned to the UU_Cfam_GSD_1.0_ROSY genome and the mitochondrial sequence was reconstructed using the procedure described above. Zero differences were observed between the mitochondrial sequence reconstructed from Illumina data and the sequence previously reported for the Zoey (Great Dane, GCA_005444595.1), Nala (German Shepherd, GCA_008641055.3), and Mischka (German Shepherd, GCA_011100685.1, the source of the UU_Cfam_GSD_1.0 assembly) genomes.

One apparent mismatch was found in the Yella (Labrador Retriever, GCA_012045015.1) mitochondrial sequence at a position where the Illumina-derived sequence contains a 'G' while the PacBio derived assembly contains an 'A'. Examination of the Illumina reads indicates that this is a site of mitochondrial heteroplasmy where the G allele fraction is 62% (*inline figure 6.1*). Thus, the PacBio assembly appears to have captured a lower frequency variant.

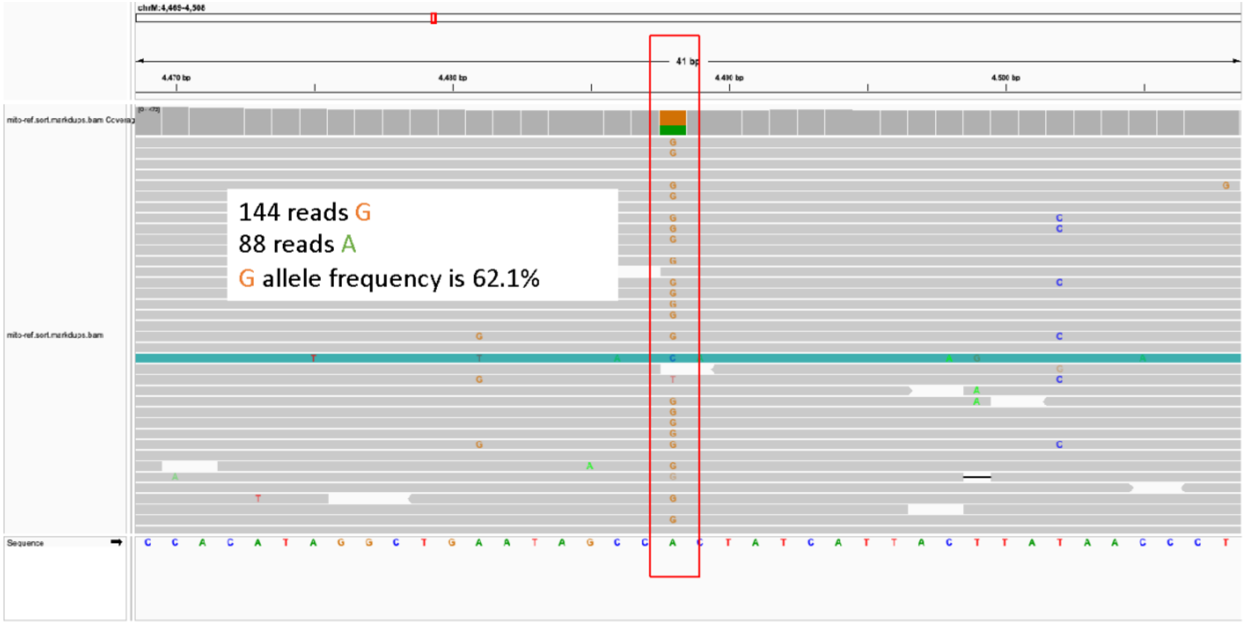

Inline figure 6.1. Identification of a heteroplasmic site captured in the Yella mitochondrial sequence. Illumina mitochondrial reads from Yella are aligned to the mitochondrial sequence released with the Yella genome assembly. An IGV screenshot of an apparent sequence difference is shown. The red box highlights the variant site and the number of reads containing the G or A alleles is shown.

An apparent mismatch was also found in the mCanLor (Grey Wolf, GCA_905319855.2) assembly at a position where the Illumina-derived sequence contains an ‘A’, in accordance with the mitochondrial reference genome sequence, and the PacBio derived sequence contains a ‘T’. Transversion mutations are rare in the canine mitochondria [61]. The ‘A’ allele is present in 98.5% of the Illumina reads, suggesting that this may be an error in the PacBio assembly (*inline figure 6.2*). Thus, across all five comparisons, zero likely errors in the Illumina-derived sequences were identified (*inline table 6.2*).

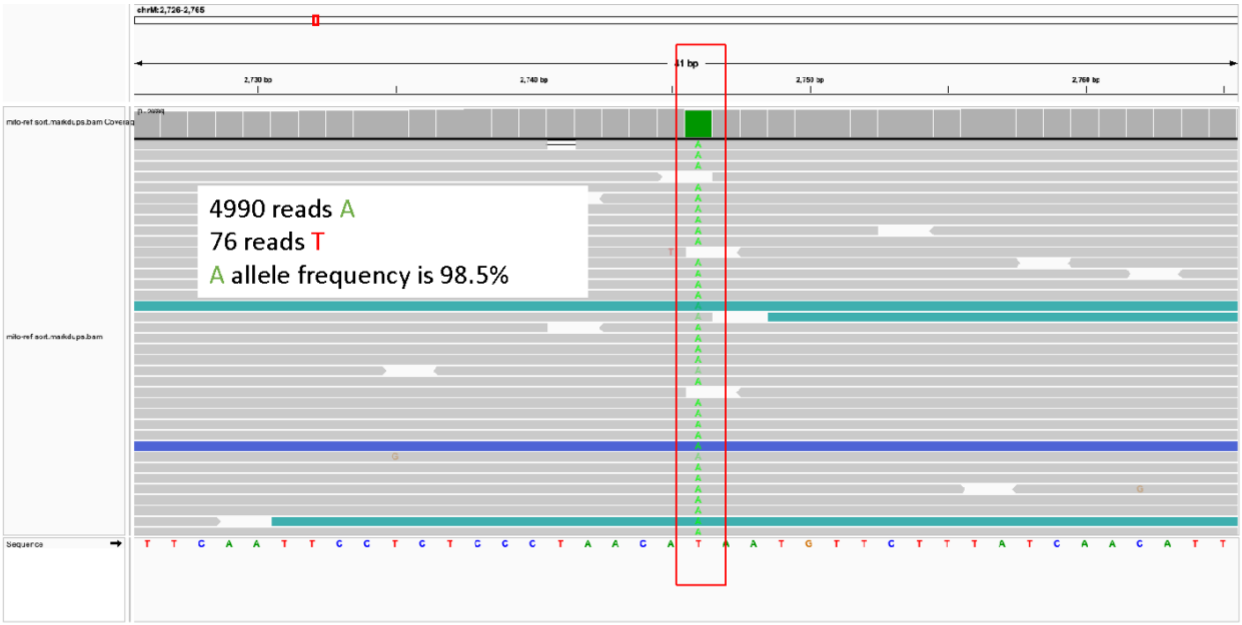

Inline figure 6.2. Identification of a potential error in the mCanLor mitochondrial sequence assembly. Illumina mitochondrial reads from mCanLor are aligned to the mitochondrial sequence released with the genome assembly. An IGV screenshot of an apparent sequence difference is shown. The red box highlights the variant site and the number of Illumina reads containing the A or T allele is shown.

Inline table 6.2. Summary of comparison with long-read assemblies.

Sample	Description	Mitochondria Coverage	Apparent Differences	Note
mCanLor	Grey Wolf	24,306	1	likely PacBio error
Mischka	German Shepherd	2,946	0	
Nala	German Shepherd	1,309	0	
Yella	Labrador Retriever	406	1	heteroplasmy in assembly
Zoey	Great Dane	990	0	

The differences observed between the mitochondrial sequence reported from each assembly with that reconstructed using Illumina data is shown for five recently published assemblies. Zero likely errors in the Illumina assemblies were identified across all five individuals.

Analysis of Dog10K samples

Using the above pipeline, we reconstructed the mitochondrial sequence for 1,933 Dog10K samples. This includes the 1,929 samples included in the SNV analysis as well as 4 coyotes that did not meet SNV inclusion criteria. The median mitochondrial sequence coverage across all samples was 952, with a range of 143-38,981. Converting to estimated mitochondrial genome copy-number compared to the observed autosomal sequence coverage yields a copy-number range of 13.3-4,260.2 with a median of 100.2. Multiple factors such as tissue source and DNA extraction technique are thought to affect the mitochondrial genome copy-number observed in whole genome sequencing data [145]. The estimated copy-number among breed dogs shows a bimodal distribution which correlated with sample provider (*inline figure 6.3*).

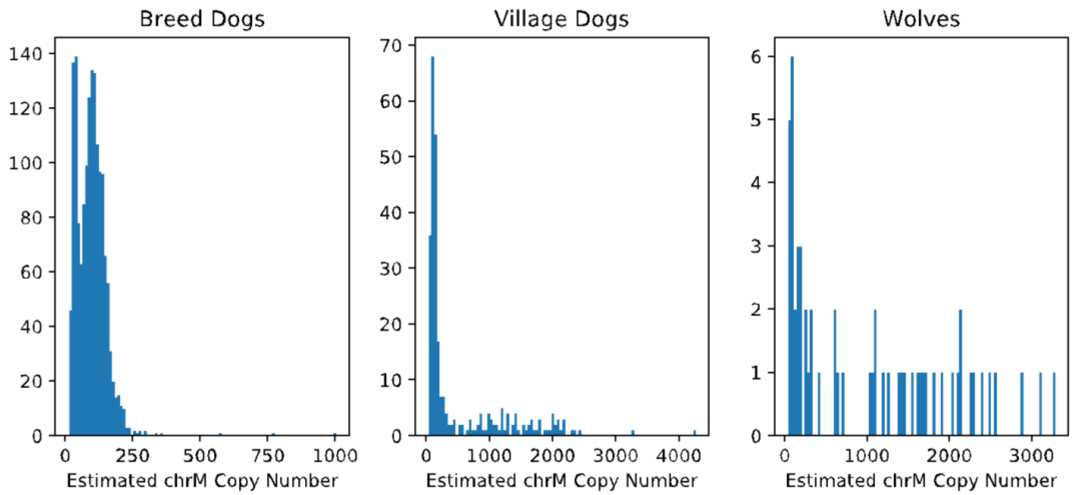

Inline figure 6.3. Estimated mitochondrial copy number by sample type. In each histogram the Y axis shows the number of samples and the X axis shows the estimated mitochondrial copy number

Across the 1,933 samples we identified 253,349 candidate polymorphisms, including heteroplasmies. The estimated alternative allele fraction for the candidate polymorphisms shows a tight clustering near the extreme values, with 95.6% of variants having an alternative allele fraction ≤ 0.05 or ≥ 0.95 (*inline figure 6.4*).

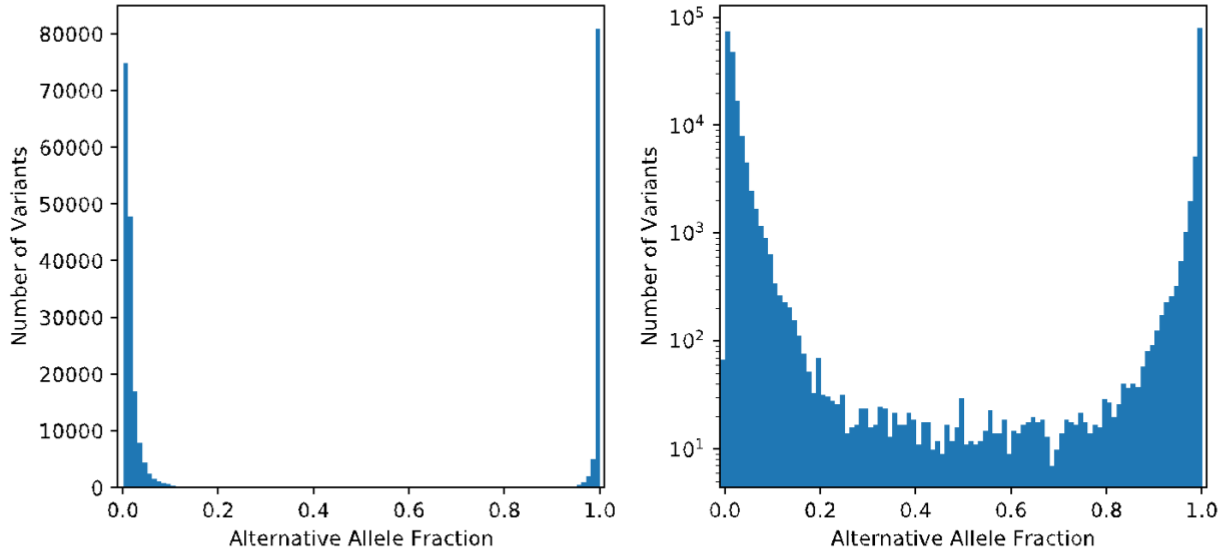

Inline figure 6.4. Distribution of alternative allele fraction for 253,349 candidate mitochondrial polymorphisms. The histogram is plotted with a linear (left) and logarithmic (right) scale on the Y-axis. Only candidates with an alternative allele fraction greater than 0.5 were retained.

We assigned each sample to a haplogroup, and created a multiple sequence alignment using Clustal Omega [179], including all 1,933 Dog10K sequences as well as published sequences from each major haplogroup. We calculated the average number of pairwise differences for each group that contained at least 3 samples (6 wolf and 18 village dog populations, based on country of origin, and 261 breeds). On average, village

dogs contain the highest level of mitochondrial diversity, but a range of variability is seen across groupings (*inline figure 6.5*).

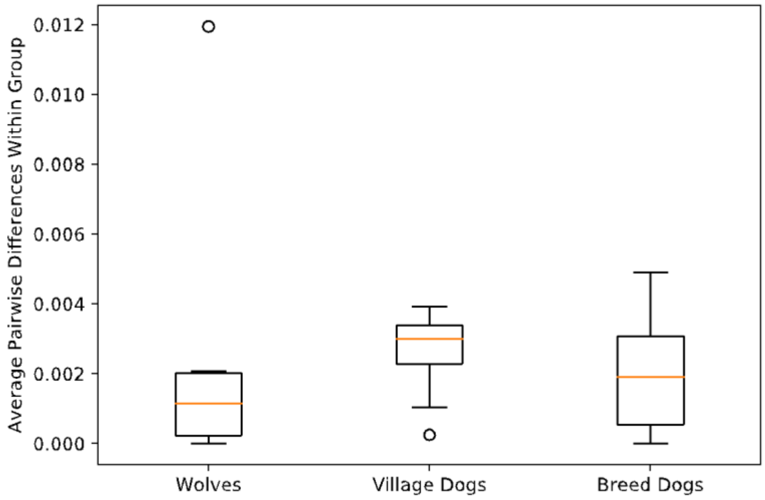

Inline figure 6.5. Mitochondrial sequence diversity within groups. Boxplots of the average pairwise mitochondrial sequence diversity is shown for 6 wolf populations, 18 village dog populations, and 261 dog breeds. Each group has at least three individuals.

Remarkably, only a single mitochondrial haplotype was found in 23 of the 261 breeds with at least 3 individuals (*inline figure 6.6*).

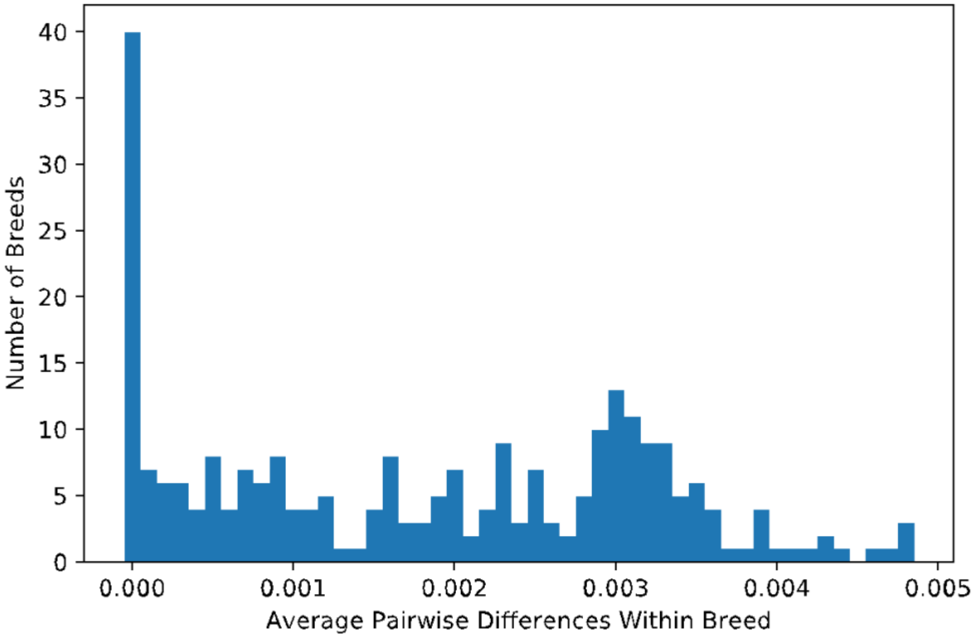

Inline figure 6.6. Average mitochondrial sequence pairwise differences within breeds. A histogram of the average number of pairwise mitochondrial sequence differences found within analyzed dog breeds is shown. Results are limited to the 261 dog breeds with at least 3 individuals. For 23 breeds there was no mitochondrial sequence variability.

There is a weak correlation between within-breed mitochondrial and autosomal sequence diversity (Spearman correlation of 0.291, P=1.7x10^{-6}) (*Inline figure 6.7*). This correlation is reduced when breeds with no mitochondrial diversity are omitted (Spearman correlation of 0.185, P=0.004).

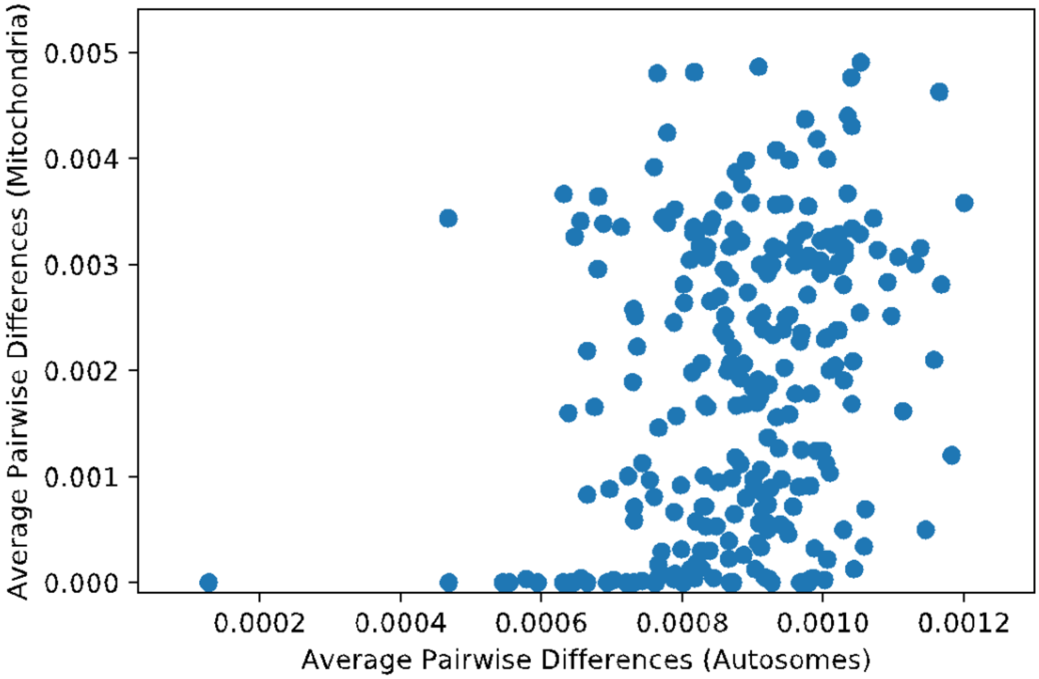

Inline figure 6.7. Correlation of mitochondrial and autosomal diversity for breed dogs. A scatter plot is shown comparing the average number of pairwise differences for the mitochondrial genomes (Y axis) and the autosomes (X axis) for 261 breeds with at least 3 individuals. Autosomal diversity was calculated based on the allele frequency spectrum at positions with no missing data and scaled based on a callable genome size of 2,143,448,215 positions that pass the callable genome filter.

Section 7 Structural variation analysis

By: Anthony K. Nguyen, Peter Z. Schall, and Jeffrey M. Kidd

We employed two complementary approaches to identify copy number variants (CNVs) and structural variants (SVs) in the Dog10K sample set: QuicK-mer2 [64], a rapid method that profiles read depth at unique k-mer positions, and Manta [66], which discovers structural variants based on discordant read-pair and split-read signatures.

QuicK-mer2 analysis

The QuicK-mer2 analysis was based on the UU_Cfam_GSD_1.0_ROSY assembly. A total of 2,054,266,273 unique 30-mers were identified using the QuicK-mer2 search command with default parameters (k=30, edit distance = 2, depth-threshold 100). Control regions for copy-number and GC normalization were created by excluding non-autosomal chromosomal sequence, regions that are duplicated in the genome assembly based on assembly self-alignment [147], reported CNVs [30], and regions with an elevated copy-number identified in a preliminary analysis using fastCN [148]. Genome wide copy-number profiles were constructed from 2,075 samples based on windows of 1,000 unique k-mers. To check the uniformity of sequence coverage, we estimated the median absolute deviation of the copy-number estimate for each sample in 2,040,588 autosomal windows. We excluded 109 samples with a median absolute deviation greater than 0.25, indicating an uneven distribution of read depth along the genome. Nearly 23% of the Village Dog samples, predominantly samples from China, were excluded due to uneven coverage. In combination with the samples previously removed due to poor SNV quality metrics, we identified a set of 1,879 samples for copy-number variation and structural variation characterization (*inline table 7.1*).

Inline table 7.1. Additional samples filtered for CNV analysis.

**Category**	**Initial Samples**	**Failed QuicKmer2 MAD Analysis**	**Failed SNV Analysis**	**Failed Both**	**Included in SV Analysis**
Breed Dogs	1649	18	70	14	1575
Coyotes	4	4	4	4	0
Mixed/Other	18	0	6	0	12
Village Dogs	336	78	55	34	237
Wolves	68	9	11	7	55
**Total**	**2075**	**109**	**146**	**59**	**1879**

Only samples that pass SNV and CNV metrics were retained for analysis.

Next, we analyzed genome wide copy-number profiles for all 1,879 samples. For male samples, values on the non-PAR region of the X chromosome were multiplied by 2. As expected, most of the genome is estimated to be diploid. Across all samples the median fraction of windows with a copy-number less than 1.5 was 0.042 and the median fraction of windows with a copy-number greater than 2.5 was 0.039.

Since QuicK-mer2 is based on unique k-mers, this profile is relative to sequence content present in the UU_Cfam_GSD_1.0_ROSY assembly. We noticed a 32kb locus with an extremely large copy-number range located at chr26:31,435,296-31,467,885. Although the region is duplicated in the UU_Cfam_GSD_1.0 assembly, QuicK-mer2 estimates a copy number of 60-70 in dogs, and into the 120s for wolves (*inline figure 7.1*). This region overlaps *LOC119866237*, the gene with the highest median copy number across all samples.

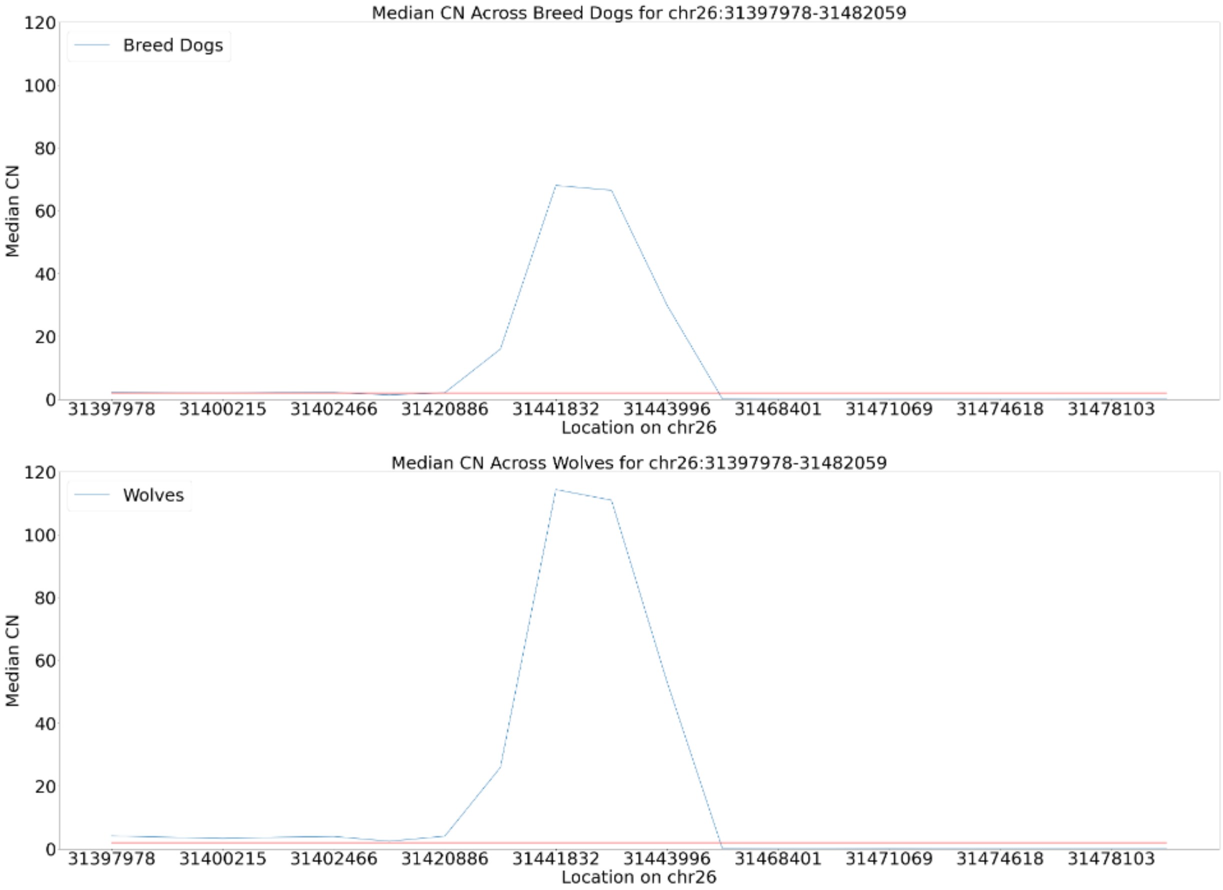

Inline figure 7.1. Median copy-number for the chr26 wolf-duplicated locus. While a duplication is present in both breed dogs and village dogs at chr26:31,435,296-31,467,885, the wolves contain a substantially higher copy number. Mixed Breed dogs and Village Dogs (not depicted) both have copy numbers in the 60s.

Other loci of notably high copy number are apparent on chromosomes 2, 5, 6, 11, and 18. Chromosome 2 contained a 237 kb region of duplication at chr2:83,392,336-83,629,428, overlapping the gene *VPS13D*, with a copy-number range of 10 across all samples. On chromosome 5 there was a 320 kb duplicated region that overlaps *LOC111096043*, a long-noncoding RNA in the same region, with a median copy number of 6**.** On chromosome 6 there was a duplication at chr6:38,456-145,747 that did not overlap with a gene, but had a median copy number of 12. Chromosome 11 contained three adjacent duplications, all within the same 815 kb block. The first, at chr11:10,940,032-11,143,374, was 203 kb long and had no overlapping gene. The second was of length 25 kb at chr11:11,251,888-11,277,364. The third was 350 kb in length at chr11:11,319,354-11,669,425. All three loci had a median copy number of 4. At chr18:18,638,669-19,125,159, there was a 486 kb locus that overlaps *MAGI2*, with a median copy number of 6. Regions

homologous to *MAGI2* have been previously found at the SOX9 locus on chr9 contributing to increased copy number and genome assembly errors (1) (*inline figure 7.2*).

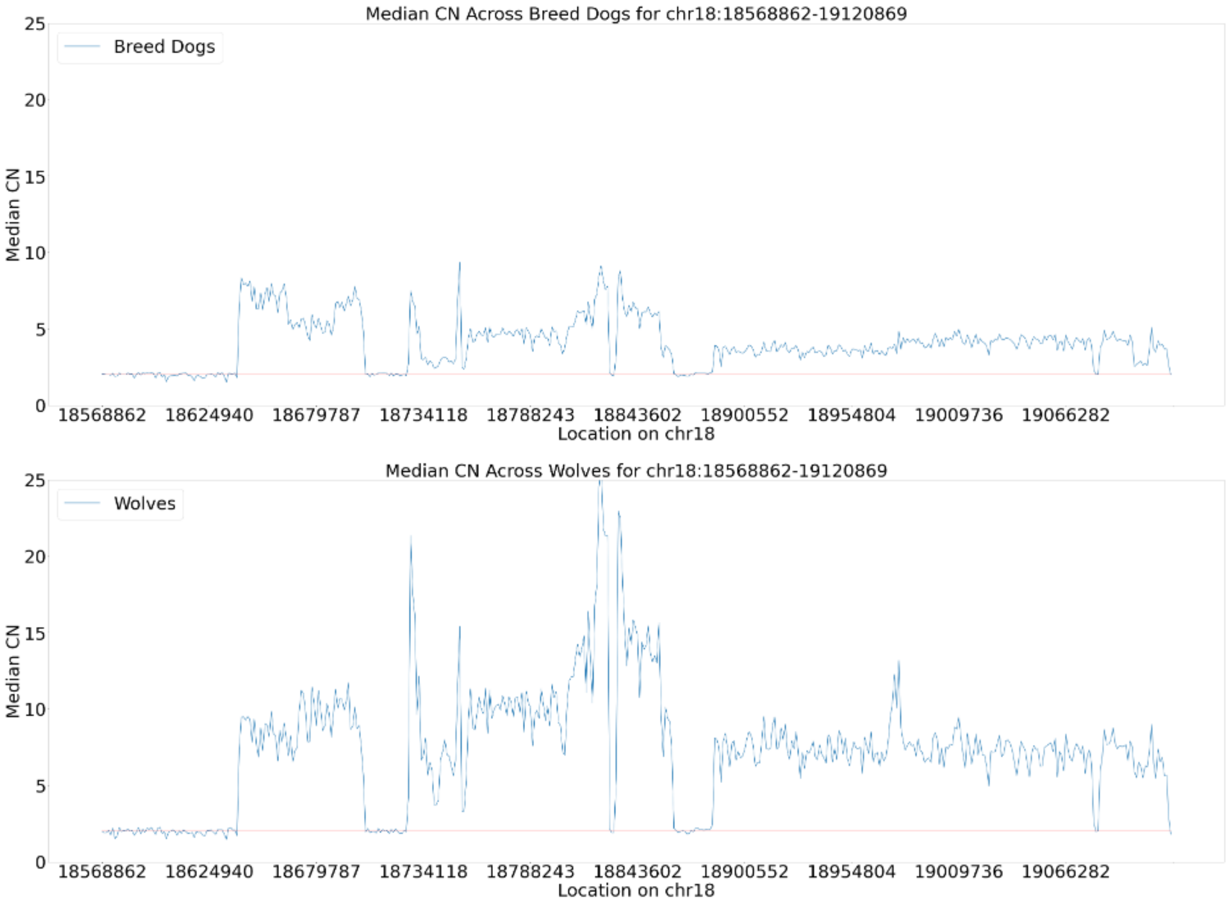

Inline figure 7.2. Median-copy number plot for the chr18 MAGI2 locus.

We estimated the paralog-specific copy number for each gene based on the median QuicK-mer2 estimate of intersecting windows for each sample. Limiting analysis to 18,162 protein coding genes that were fully encompassed by at least one k-mer window, we found 114 genes with a median copy number less than 1, suggesting that the UU_Cfam_GSD_1.0_ROSY assembly contains a rare or highly diverged structure for these genes. We also found 22 genes with a median copy number greater than 3. An increase in copy number in the amylase gene, *AMY2B (*annotated as *LOC607460*), in modern dogs is reflective of an increased ability to digest starch [180]. Consistent with previous studies [65, 181], breed dogs show a wide range of amylase copy-number with a median value of 12.4 while wolves are largely fixed for two copies (*inline figure 7.3*).

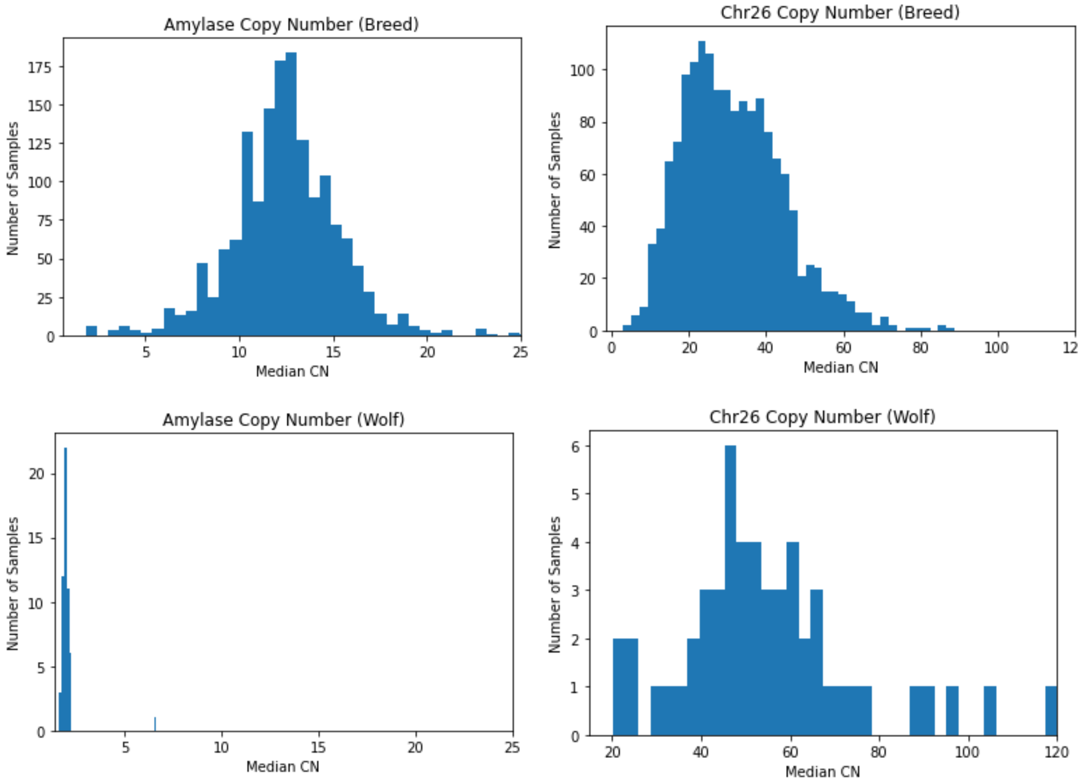

Inline figure 7.3. Copy-number estimates for amylase and the chr26 duplication. The distribution of copy-number estimated for breed dogs (top) and wolves (bottom) is shown for pancreatic amylase (*AMY2B*, *LOC607460*, left) and for a duplication of a 32 kbp region on chr26 (chr26:31435958-31468401, right). One wolf sample, CLUPRU000011, is estimated to have ~7 copies of the amylase gene.

Structural variant analysis with Manta

We identified candidate structural variants in 1,879 samples using Manta version 1.6.0 with default parameters [66]. Inversions were converted to event representation using the convertInversion.py utility distributed with Manta. Raw calls were merged using *svimmer* and genotyped across all samples using GraphTyper2 version 2.7.2 with default parameters [67]. All analyses were limited to the primary assembled chromosomes (chr1-38 + chrX). For break-end (BND), insertion (INS), deletion (DEL) and duplication (DUP) calls, the 'AGGREGATED' genotyping model was used. For inversion (INV) candidates, the breakpoint model was used as reported by GraphTyper2. Variants were filtered using vcffilter with the following command:

vcffilter -f "(SVTYPE = BND & SVMODEL = AGGREGATED & QD > 20 & (ABHet > 0.30 | ABHet < 0) & (AC / NUM_MERGED_SVS) < 10 & PASS_AC > 0 & PASS_ratio > 0.1) | (SVTYPE = DEL & SVMODEL = AGGREGATED & QD > 12 & (ABHet > 0.30 | ABHet < 0) & (AC / NUM_MERGED_SVS) < 25 & PASS_AC > 0 & PASS_ratio > 0.1) | (SVTYPE = DUP & SVMODEL = AGGREGATED & QD > 5 & PASS_AC > 0 & (AC / NUM_MERGED_SVS) < 25) | (SVTYPE = INS & SVMODEL = AGGREGATED & PASS_AC > 0 & (AC / NUM_MERGED_SVS) < 25 & PASS_ratio > 0.1 & (ABHet > 0.25 | ABHet < 0) & MaxAAS > 4) | (SVTYPE = INV & PASS_AC > 0

& (AC / NUM_MERGED_SVS) < 25 & PASS_ratio > 0.1 & (ABHet > 0.25 | ABHet < 0) & MaxAAS > 4)"

This resulted in a total of 147,113 structural variants that were successfully genotyped (*inline table 7.2*).

Inline table 7.2. Summary of structural variants identified and genotyped across 1,987 individuals.

Variant Type	Raw Merged Calls	Pass GraphTyper 2 Filters
BND	2,270,095	189
DEL	166,416	75,337
DUP	29,819	3,453
INS	220,651	66,328
INV	10,372	1,806
Total	2,697,353	147,113

BND: Breakpoint end, DEL: Deletion, DUP: Tandem duplication, INS: Insertion, INV: inversion

Structural variation calls from Manta (v1.6.0) in VCF format were converted to BEDPE with svtools (v0.5.1) [182] and imported into R (v4.1.0, RRID:SCR_002394). Resultant structural variants were filtered to include insertions, deletions, duplications, and inversions that were less than 10 Mb.

We assessed LD between structural variants and SNVs using PLINK. Analysis was limited to autosomal structural variants with a minor allele frequency of at least 1%. SNVs within 200 kb of each structural variant were assessed with an r^2 cutoff of 0.8. Using an r^2 cutoff of 0.8, we find that 43.8%-64.7% of SVs are in strong LD with a SNV (*inline table 7.3*). The lower LD found with duplications likely reflects both a higher mutational recurrence rate and lower genotype accuracy found with this SV type.

Inline table 7.3 Linkage disequilibrium between structural variants and SNVs

SV Type	Tested SVs	SVs with tag SNV	Percent Tagged
Deletions	68,119	44,068	64.7%
Insertions	50,629	29,633	58.6%
Duplications	3,005	1,317	43.8%

LD was calculated between deletion, insertion, and duplication variants and SNVs using PLINK. Analysis was limited to autosomal structural variants with a minor allele frequency of at least 1%. SNVs within 200 kb of each structural variant were assessed with an r^2 cutoff of 0.8.

Insertion and deletion variants were further queried for their respective intersection with coding exons. Annotation of structural variants was conducted using SnpEff v4.3 (RRID:SCR_005191) [150], after the

creation of a custom SnpEff database using the UU_Cfam_GSD_1.0_ROSY assembly and associated GTF file. A total of 31,950 deletions were identified that intersected a total of 12,522 genes. However, many of the deletions were rare across the sequenced samples. Filtering deletions to those present in at least 10% of the samples decreased the number of variant sites to 17,171, intersecting with 8,267 genes. The length of the intersecting deletions had a range of 50-9,862,940 bp, with a mean of 477,269 bp and median of 211 bp. The percent of samples exhibiting deletions ranged from 10.01-100% (allele frequency: 0.05-1.0), with a mean of 72.00% (allele frequency: 0.55), and a median of 80.47% (allele frequency: 0.55). Due to the range of length of the deletions, some structural variants impacted multiple genomic feature types (e.g., intron and exon, splice region and untranslated region, etc.). Introns were the largest category intersecting with deletions, followed by splice regions, and then frameshifts (*inline table 7.4*).

Inline table 7.4. Summary of deletions and insertions intersecting with genomic features.

Feature	Deletions	Insertions
Intron	16,776	14,201
Splice Region	535	15
Frameshift	237	33
3' UTR	157	107
Exon Loss	150	0
5' UTR	78	35
Stop Lost	22	1
Stop Gained	2	19

To identify functional variants restricted to a subset of breeds, the frequency of genic deletions and insertions were binned by breed category, limiting to those breeds with at least 4 samples. Across this breed-based population, variants were filtered to include those meeting these parameters: 1) presence in >=90% in one breed and >30% in <3 breeds or 2) presence <=10% in one breed and >90% in >150 breeds. This allowed for the identification of rare variants enriched within a specific breed, as compared to the UU_Cfam_GSD_1.0_ROSY assembly, and the inverse, those variants not present in a specific breed while present in the population at-large. A total of 341 variants fit the definition of the rare variants, which were present across 94 breeds. Four breeds (Norwegian Lundhound, Japanese Akita, Czechoslovakian Wolfdog, and the Saarloos Wolfdog) each had more than 20 of the rare insertions/deletions, and together these four breeds represented 34% (n=116) of the rare variants. Those variants that are well supported across the population, while rare in a single breed, numbered 426 across 96 breeds. As with the rare variants, four breeds (Norwegian Lundhound, Czechoslovakian Wolfdog, Bohemian Shepherd, and the Bull Terrier) all had more than 20 variants, representing 31% of the total variants.

A locus identified with QuicK-mer2 and Manta.

FGF4 retrogenes, which encode for Fibroblast growth factor 4, lead to the short leg phenotype present in multiple dog breeds[68-70], while a 133kb duplication that spans multiple genes, including *FGF4*, is responsible for the dorsal hair ridge found in Rhodesian and Thai Ridgebacks [71]. Our QuicK-mer2 analysis identified variation due to both events at the *FGF4* locus (*inline figure 7.4*). The 133 kbp duplication was also detected by Manta and is present in all analyzed Rhodesian and Thai Ridgebacks as well as three village dogs from Africa (VILLCG000006 (Congo), VILLKE000001 (Kenya), and

VILLLR000017 (Liberia)). Since *FGF4* is a small gene with short introns, the k-mer based analysis identified a spike of increased copy number associated with the presence of an *FGF4* retrogene in short-legged breeds.

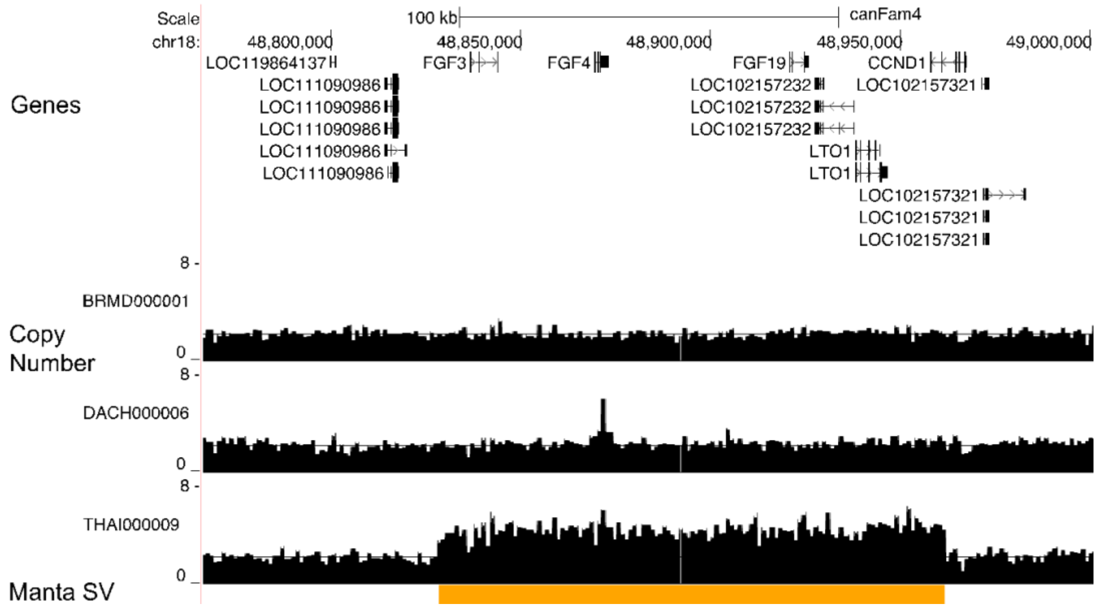

Inline figure 7.4. Multiple variants at the FGF4 locus. A genome browser view showing the estimated copy number at the *FGF4* locus is depicted. Gene annotations are shown at the top of the figure, followed by QuicK-mer2 copy-number estimated given in windows containing 1000 unique k-mers in three samples. A horizontal line indicates the normal diploid copy number of 2. The orange bar at the bottom indicates the position of a tandem duplication identified by Manta. The top sample, BRMD000001, a Bernese Mountain Dog, has a diploid copy-number of two throughout the depicted region. The middle sample, DACH000006, a Dachshund, has an increased copy-number restricted to the *FGF4* locus. Examination of individual reads confirms that the detected increase is due to the presence of a retrogene. The bottom sample, THAI000009, a Thai Ridgeback, has a ~133 kbp duplication that spans multiple genes.

Section 8 Selection in breed groups

By: Katia Bougiouri, Tatiana Feuerborn, Laurent Frantz, and Fernando Racimo

Analysis using Ohana

We used Ohana [78] to detect regions of the genome under positive selection in different groups of dogs. This method leverages information about extreme allele frequency differences and incorporates admixture between populations to detect selection signatures specific to ancestry components. It uses a latent mixture model to estimate ancestral population components and ancestral allele frequencies. These frequencies are then used to compute a population covariance matrix of the ancestral components and infer a tree from it. Selection scans are subsequently carried out by testing for covariance outliers that can be explained by excessively long branches subtending one or several of the ancestral components.

The use of Ohana has two main advantages compared to previous methods of detecting selection: 1) It simultaneously models admixture and tests for selection, therefore avoiding the need to specify populations *a priori*, and 2) It can analyze multiple populations simultaneously by detecting selection signatures specific to ancestral components or sets of components. This method can therefore facilitate the detection of selection in admixed populations with unclear evolutionary histories and can even test if the selection signal is shared among multiple populations.

Here we used this method to detect signals of selection shared across nine groups of dog breeds which possess similar morphological traits, representing a total of 790 samples (Additional File 1: Table S21):

- **Spitz:** Hallefors Elkhound (1), Norwegian Elkhound (7), American Eskimo Dog (6), Norwegian Buhund (5), Pomeranian (7), Norrbottenspitz (5), Swedish White Elkhound (7), Swedish Vallhund (4), Icelandic Sheepdog (6), German Spitz Klein (11), Swedish Lapphund (3), Finnish Spitz (6), Volpino Italiano (6), Keeshond (11), German Giant Spitz (6), Swedish Elkhound (4), German Spitz (6), Japanese Spitz (12), German Spitz Mittel (12)
- **Sighthounds**
  - African sighthound: Sloughi (3), Azawakh (1)
  - Iberian sighthounds: Pharaoh Hound (6), Ibizan Hound (13), Cirneco dell'Etna (6)
  - Middle Eastern sighthound: Tazi (2), Afghan Hound (1), Saluki (3)
  - UK sighthound: Scottish Deerhound (3), Silken Windhound (2), Whippet (6), Magyar Agar (6), Hortaya Borzaya (2), Galgo Espanol (6), Polish Greyhound (7)
- **Waterdogs:** Lagotto Romagnolo (3), Spanish Water Dog (4), Barbet (6), Portuguese Water Dog (8)
- **Scenthounds:** Petit Basset Griffon Vendeen (11), Hanoverian Scenthound (1), Artois Hound (2), Porcelaine (1), Alpine Dachsbracke (7), Lowchen (4), Bruno Jura Hound (5), Russian Hound (6), American Foxhound (7), Petit Bleu de Gascogne (4), Great Anglo-French Tricolour Hound (6), Griffon Nivernais (6), Otterhound (7), Slovensky Kopov (2), Small Swiss Hound (12), Bavarian Mountain Scent Hound (6), Griffon Fauve de Bretagne (1), Great Anglo-French White and Orange Hound (6), Grand Griffon Vendeen (2), Drever (6), Bloodhound (4), Dachshund (17), Basset Fauve de Bretagne (5), Plott Hound (1), Zagar (7), Grand Bleu de Gascogne (1), Redbone Coonhound (3), Harrier (3), Posavac Hound (1), Basset Hound (7), Gotland Hound (1), Blue Gascony Griffon (2),

Hellenic Hound (1), Grand Basset Griffon Vendeen (5), Segugio Italiano (4), Briquet Griffon Vendeen (2), Basset Artesien Normand (6), Billy (2), Anglo-Francais hound (4), German Hound (6), Treeing Walker Coonhound (1), Bluetick Coonhound (2), English Foxhound (2), Swiss Hound (12), Finnish Hound (6), Ariegeois (1), Black and Tan Coonhound (1), Hamiltonstovare (7), Estonian Hound (3)

- **Pointers:** French Spaniel (6), German Wirehaired Pointer (5), Irish Red and White Setter (1), Auvergne Pointer (6), Wirehaired Pointing Griffon (6), Dutch Partridge Dog (1), English Setter (3), Small Munsterlander (11), Spinone Italiano (7), English Pointer (8), Weimaraner (5), Saint-Usuge Spaniel (1), Slovakian Wirehaired Pointer (4), Braques Francais (5), Bracco Italiano (6), Brittany (8), Gordon Setter (1), Pudelpointer (3), Bourbonnais Pointing Dog (7), Irish Setter (3), Cesky Fousek (4), Portuguese Pointer (1), Picardy Spaniel (4), Vizsla (7), Large Munsterlander (1), German Shorthaired Pointer (4), Ariege Pointer (6), Pont-Audemer spaniel (6), Blue Picardy Spaniel (3), Catalburun (3)
- **Belgian herders:** Dutch Shepherd (5), Belgian Sheepdog (3), Picardy Shepherd (6), Bouvier des Ardennes (1), Bouvier des Flandres (2), Belgian Malinois (4), Groenendael (3), Belgian Laekenois (5), Belgian Tervuren (5)
- **UK herding:** Australian Shepherd (3), Cardigan Welsh Corgi (4), Shetland Sheepdog (4), Australian Cattle Dog (4), Australian Kelpie (6), Pembroke Welsh Corgi (1), Collie (13), Old English Sheepdog (7)
- **Spaniels:** Cocker Spaniel (3), Irish Water Spaniel (4), American Water Spaniel (6), Clumber Spaniel (5), Field Spaniel (5), Boykin Spaniel (5), Cavalier King Charles Spaniel (5), Welsh Springer Spaniel (4), Sussex Spaniel (6), English Cocker Spaniel (3), German Spaniel (6), Curly-Coated Retriever (4), English Springer Spaniel (3),
- **Mastiffs:** Majorca Mastiff (3), Bull Terrier (4), Olde English Bulldogge (3), French Bulldog (6), Dogue de Bordeaux (5), Continental Bulldog (6), Bullmastiff (6), Boxer (5), American Bulldog (5), Miniature Bull Terrier (4), Dogo Canario (6), English Bulldog (2), Boerboel (4), Boston Terrier (3), Mastiff (6), Staffordshire Bull Terrier (6), Dogo Argentino (5), American Staffordshire Terrier (5)

We only included biallelic SNPs with a PASS flag and a minor allele frequency (MAF) of >5%. We only kept sites without missing genotype data. This resulted in a total of 6,181,086 autosomal sites used as input for the selection scans. We ran Ohana using a number of ancestral components ranging from K=2 up to K=11. Ohana admixture plots for K from 2 to 11 are shown (*inline figure 8.1*).

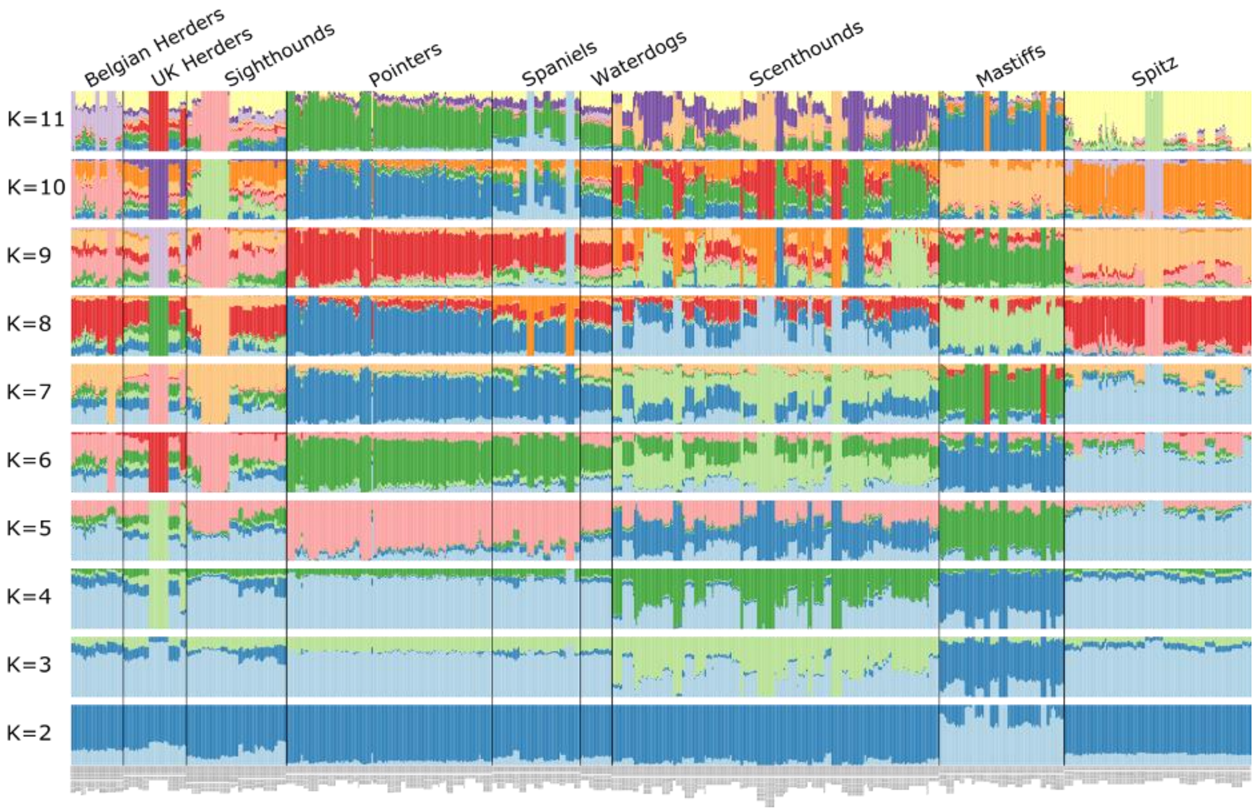

Inline figure 8.1. Admixture plots for K=2 up to K=11 as inferred from Ohana for 790 samples. All nine dog groups are labeled at the top and samples belonging to each group are separated with black vertical lines.

We chose K=5 as a compromise between low risk of over-fitting (see Q-Q plots *inline figure 8.2*) and interpretability of component identity. The five inferred ancestral components were maximized for the following dog groups: Mastiffs, Scenthounds, Spitz, Pointers and Spaniels, and the Collie and Shetland Sheepdog.

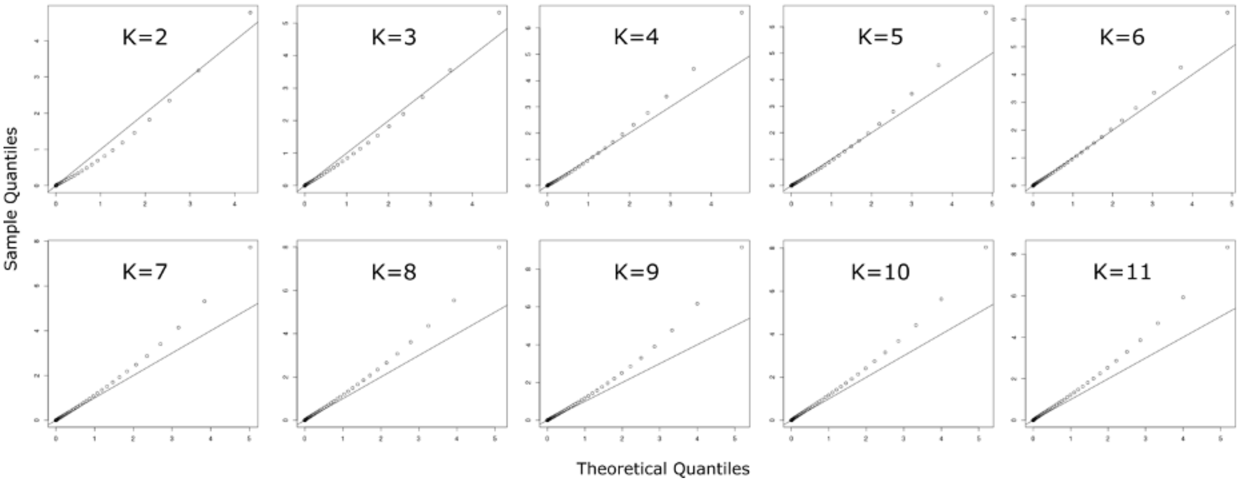

Inline figure 8.2. Q-Q plots comparing the sample quantiles against the theoretical quantiles coming from a mixed chi-square distribution for K=2 to 11.

The log-likelihood ratio test statistic of Ohana's selscan module was used to evaluate the likelihood of selection for each variant. Genomic control was carried out and p-values were calibrated using a mixed chi-squared distribution with the "emdbook" R package (version 1.3.12) [149]. A Bonferroni threshold [-log(0.05/number of analyzed sites)] was used as a threshold value for significance ($P_{bonferroni}$=8.09). We note that this threshold is necessarily quite strict as many of the sites are highly correlated due to linkage. We used the 'intersect' function of BEDTools v2.30.0 [140] and the UU_Cfam_GSD_1.0 annotation to identify genes overlapping or within 100 kb of the significant sites.

We did not further examine the results for the component which was maximized for the Collies and Shetland Sheepdogs due to the low number of samples and the strong inbreeding observed in these breeds (see Supplementary Methods Section 5). Candidate genes which were either overlapping within 100 kb of the selected regions for each targeted ancestry are presented in Additional File 1: Table S9.

Fine-mapping using iSAFE

Results of running iSAFE [93] on 13 of the 15 identified loci are shown below. Two loci (chr26 and chr38 in the component maximized in Mastiffs) are too large for iSAFE analysis and are omitted. In each figure the Y-axis gives the iSAFE score. The dashed line corresponds to an iSAFE score threshold of 0.1. Variants above this threshold are colored green. Those variants that also have a significant signature of selection based on the Ohana analysis are colored red. Annotated genes are plotted for each locus. The variants with the top iSAFE scores for each locus are given in Additional File 1: Table S10.

Spaniels-Pointers

chr16: 55473955-55474254

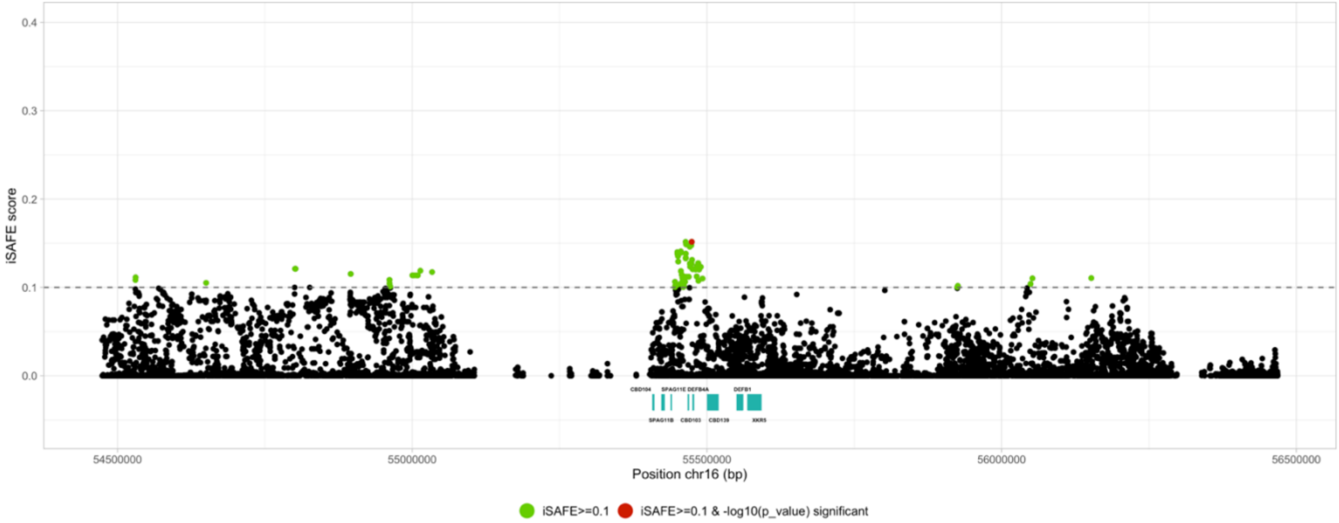

Spitz

chr1: 93899377-93958228

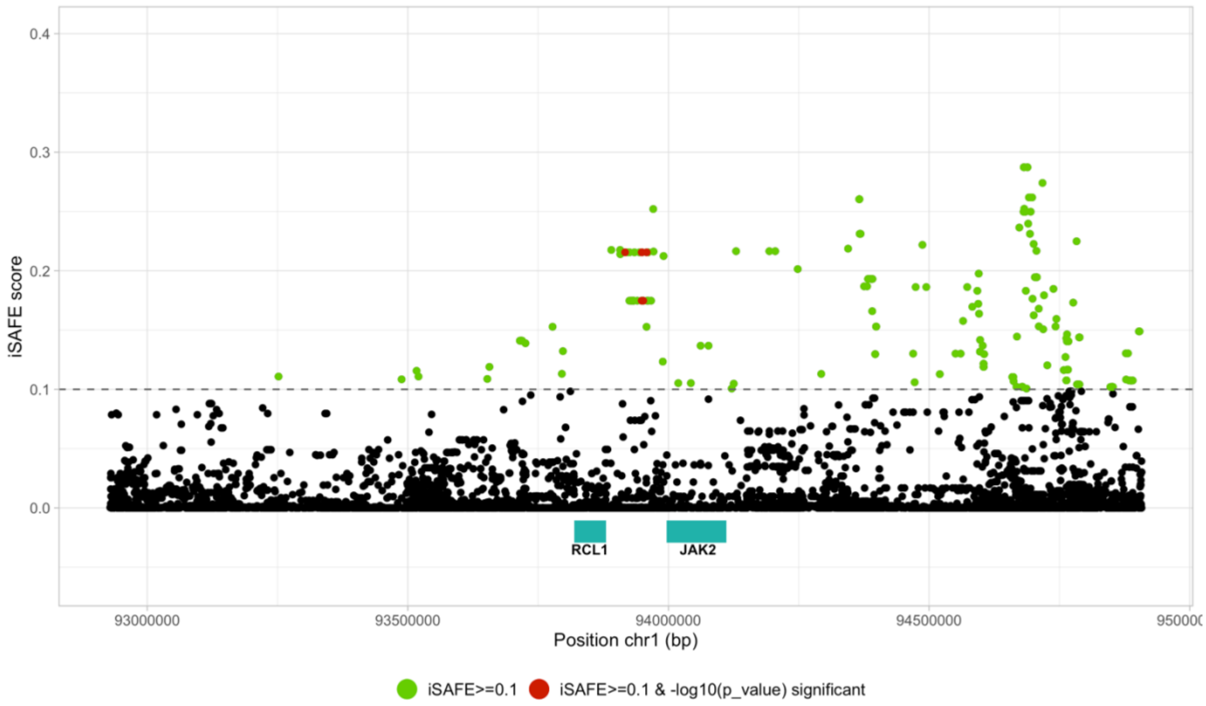

chr10: 8137458-8986053

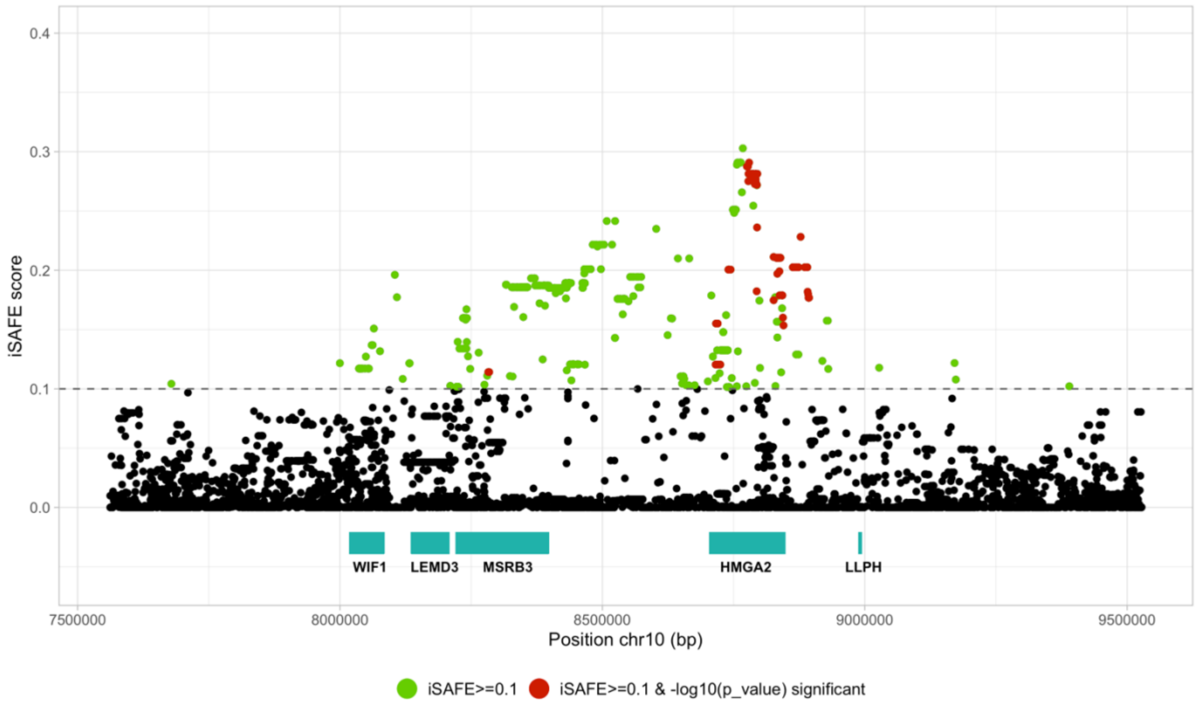

chr10:67223309-67223309

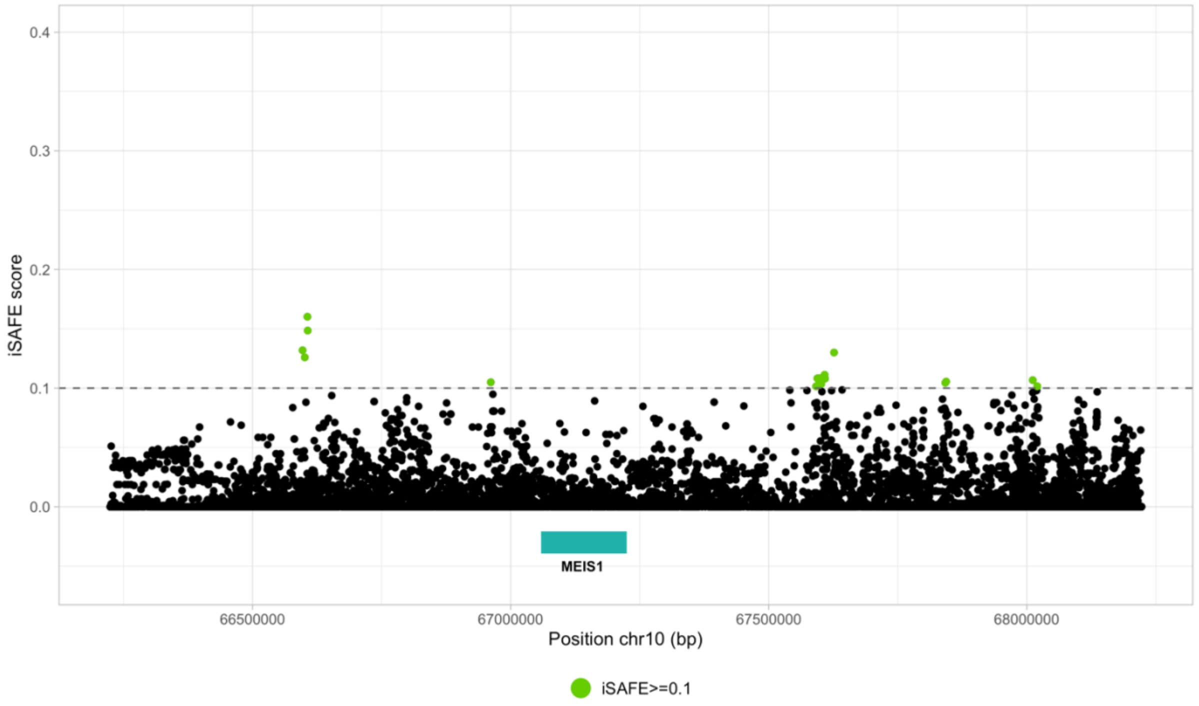

chr28:25156924-25166920

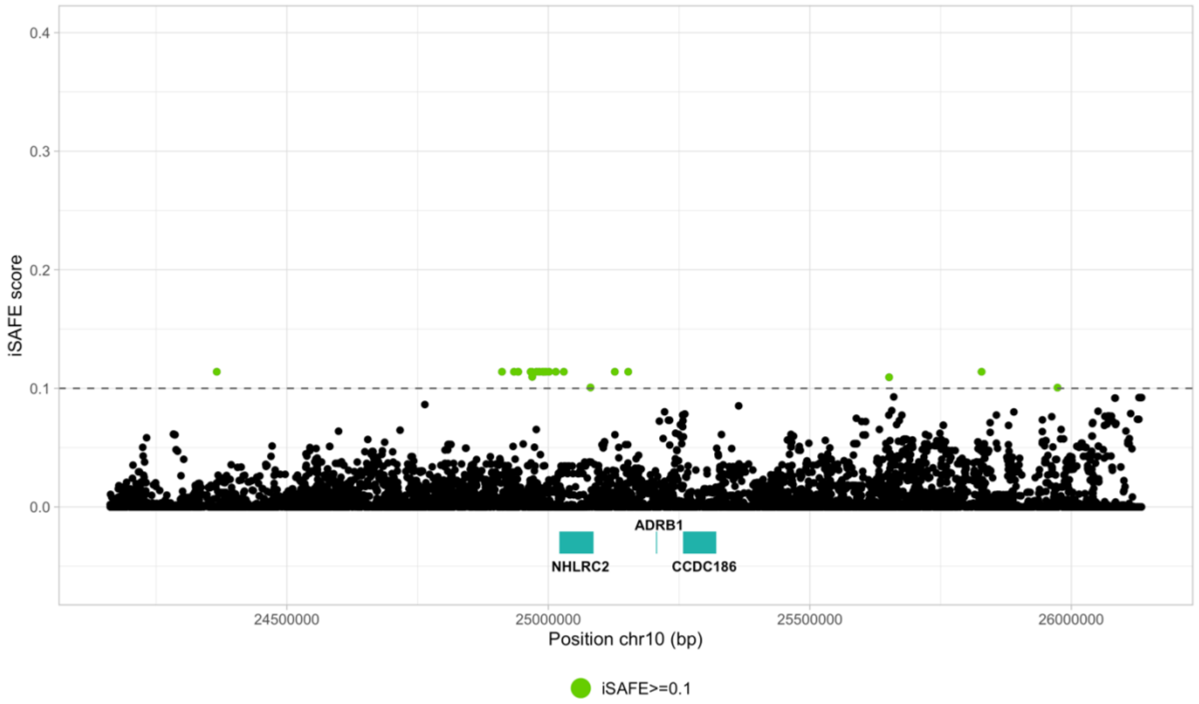

Scenthounds

chr11:36619547-37215297

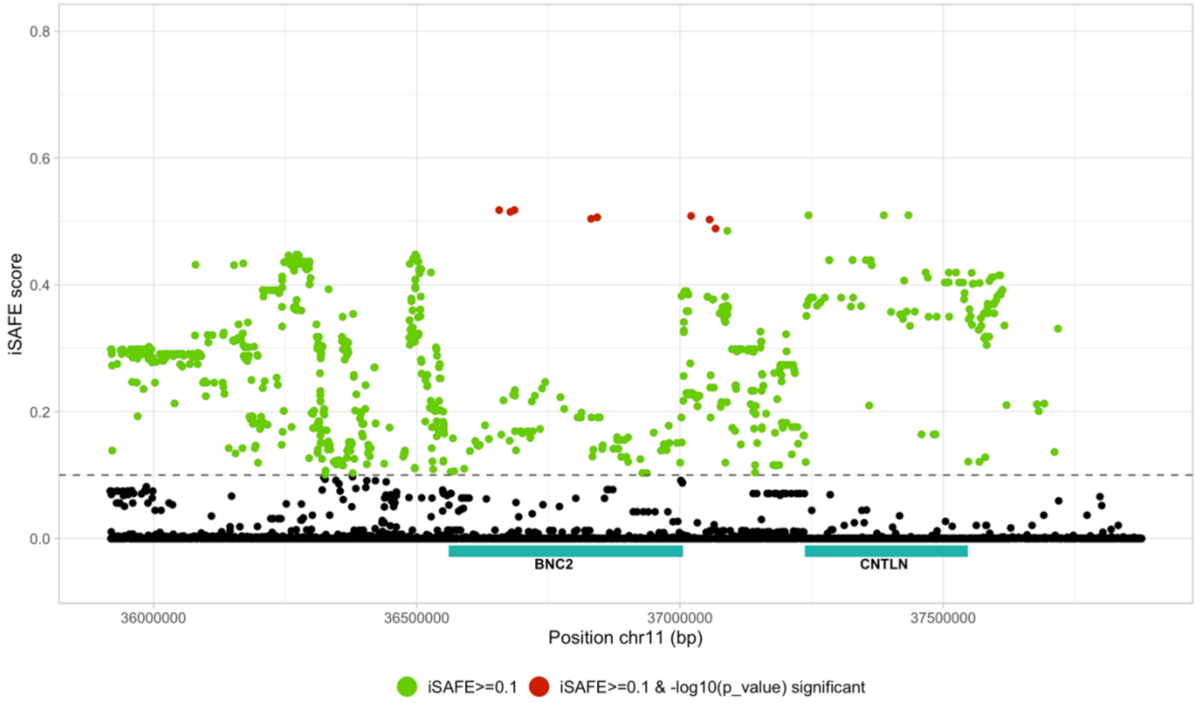

chr24:23625523-24163404

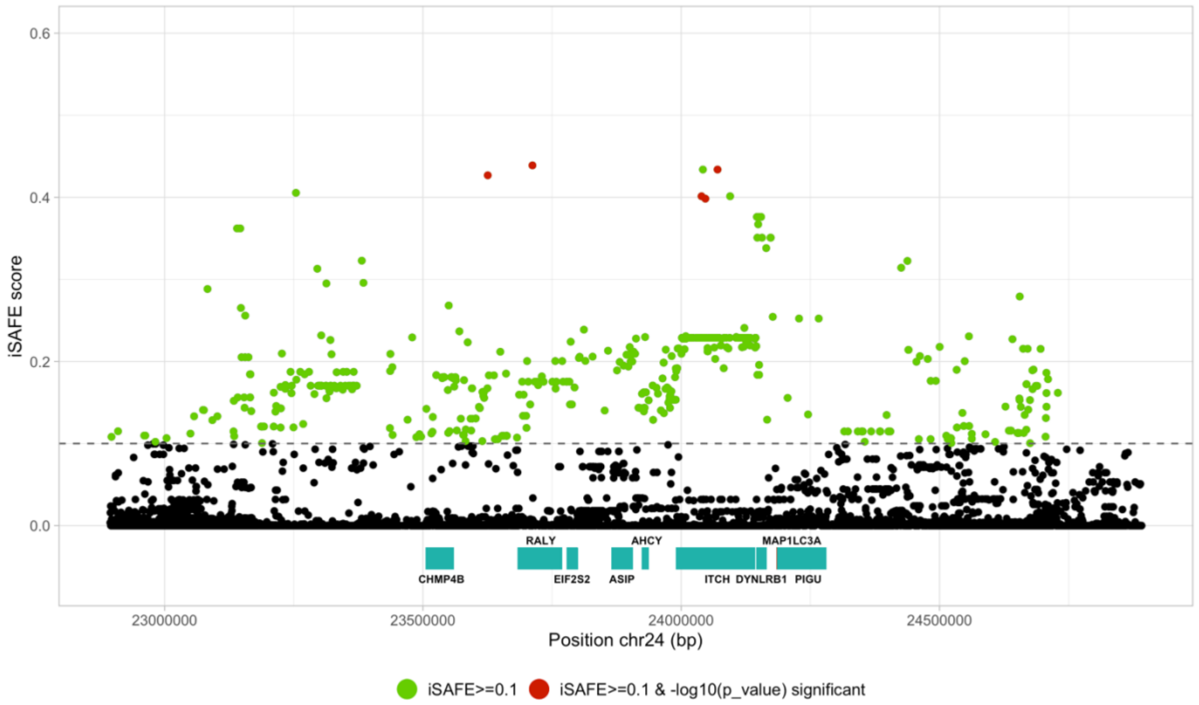

Mastiffs:

chr1:52369802-53844285

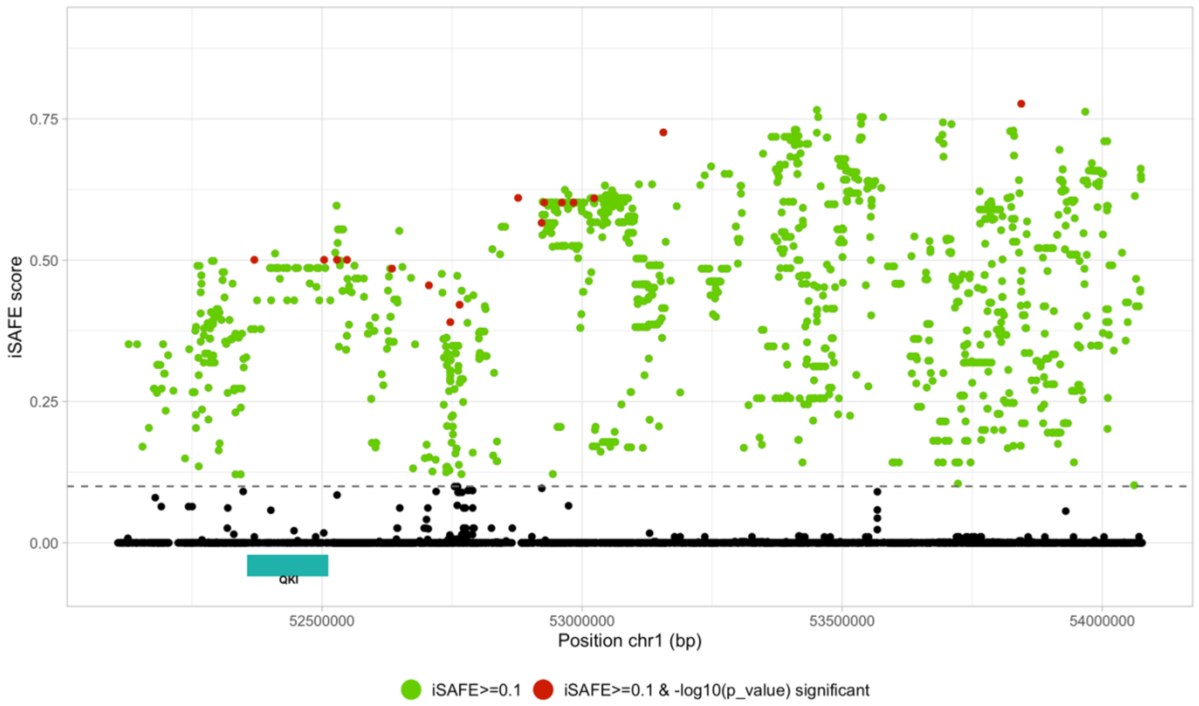

chr6:69385669-69385669

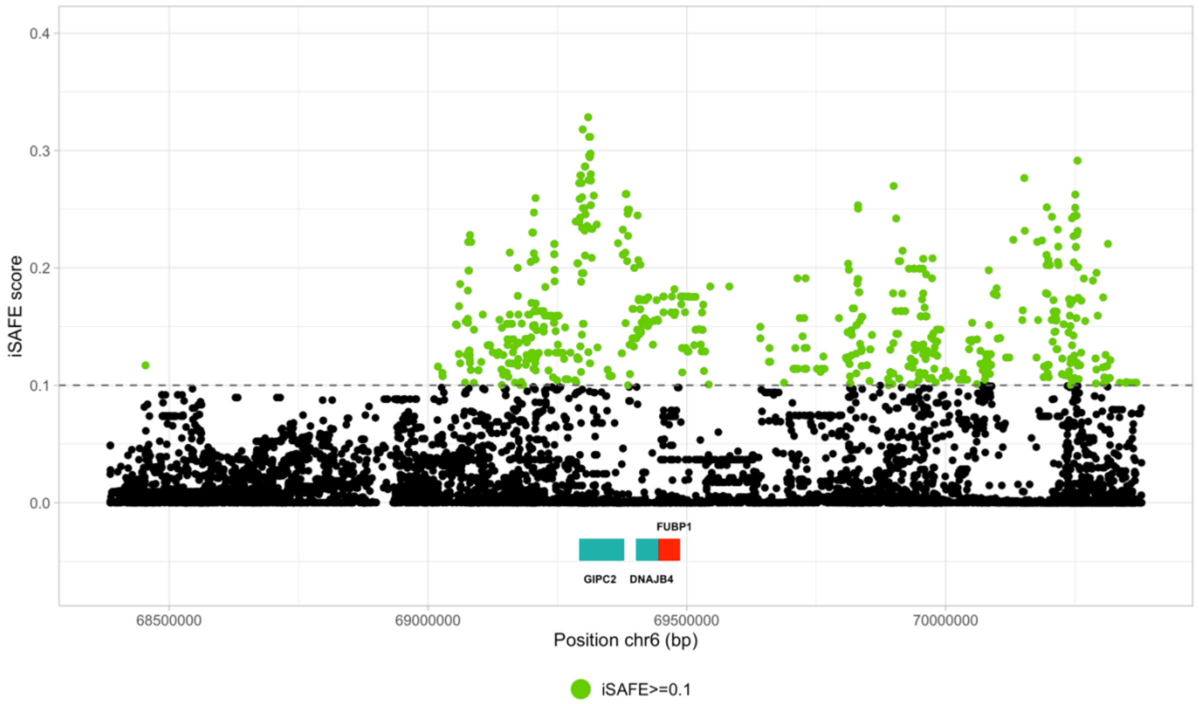

chr9:42047014-42176961

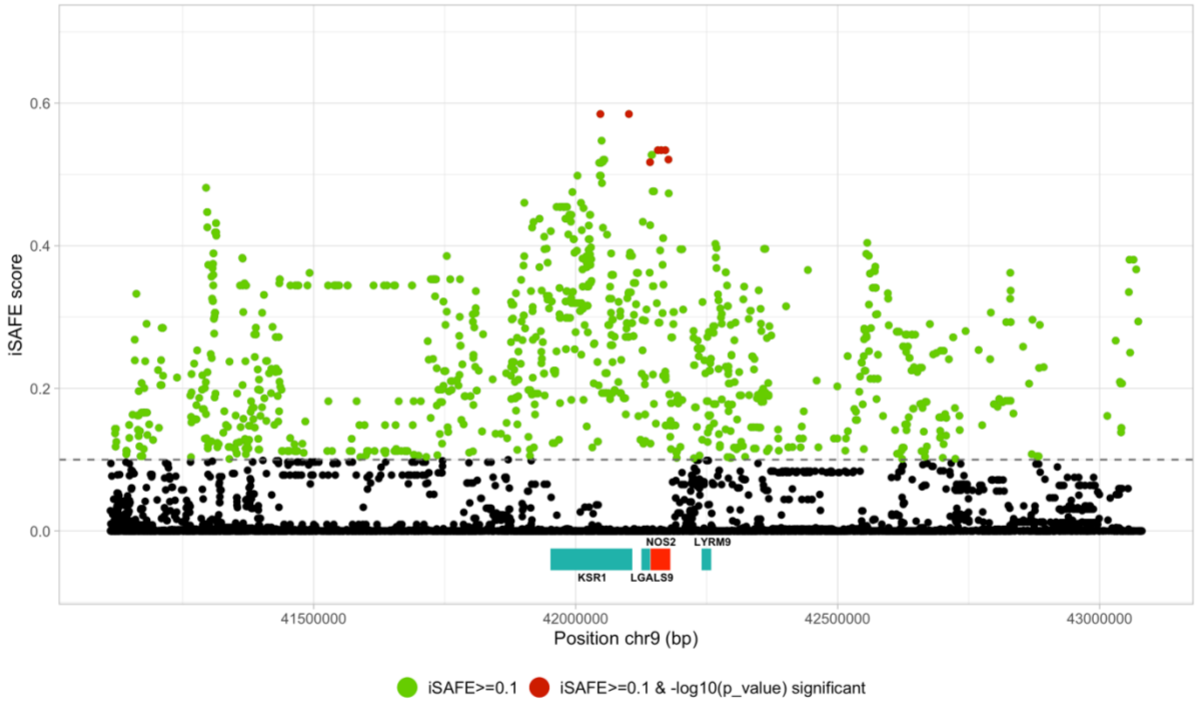

chr10:7270898-7341150

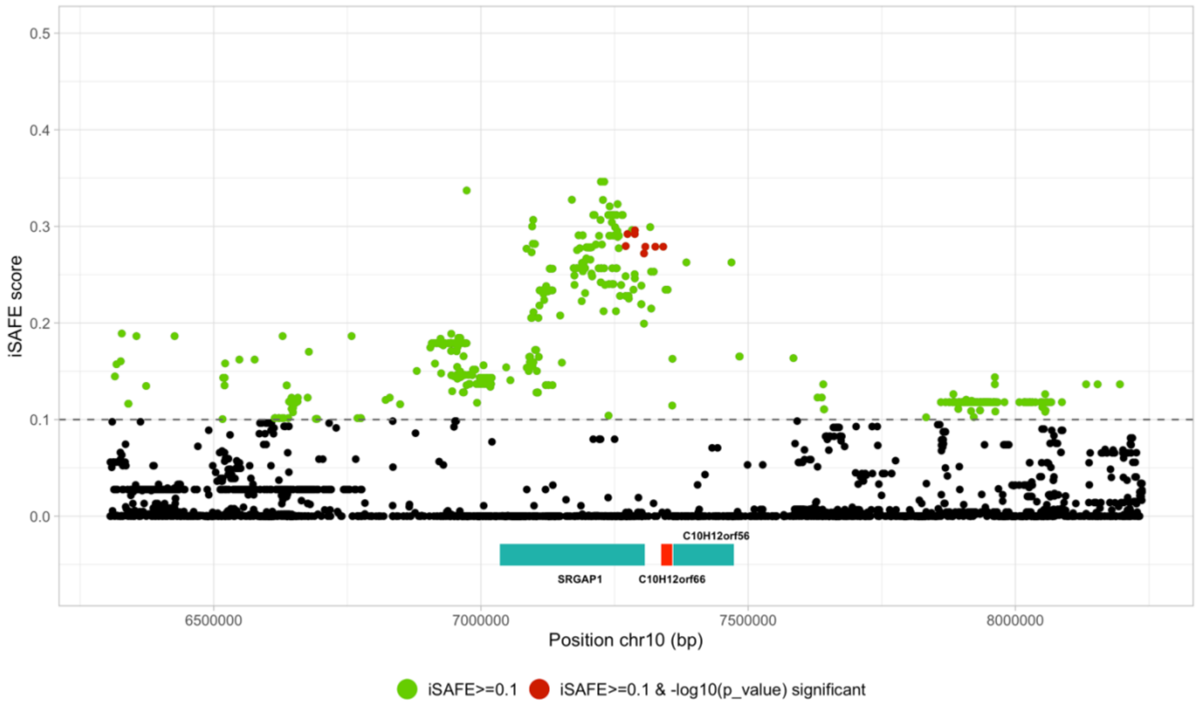

chr20:29500160-29520764

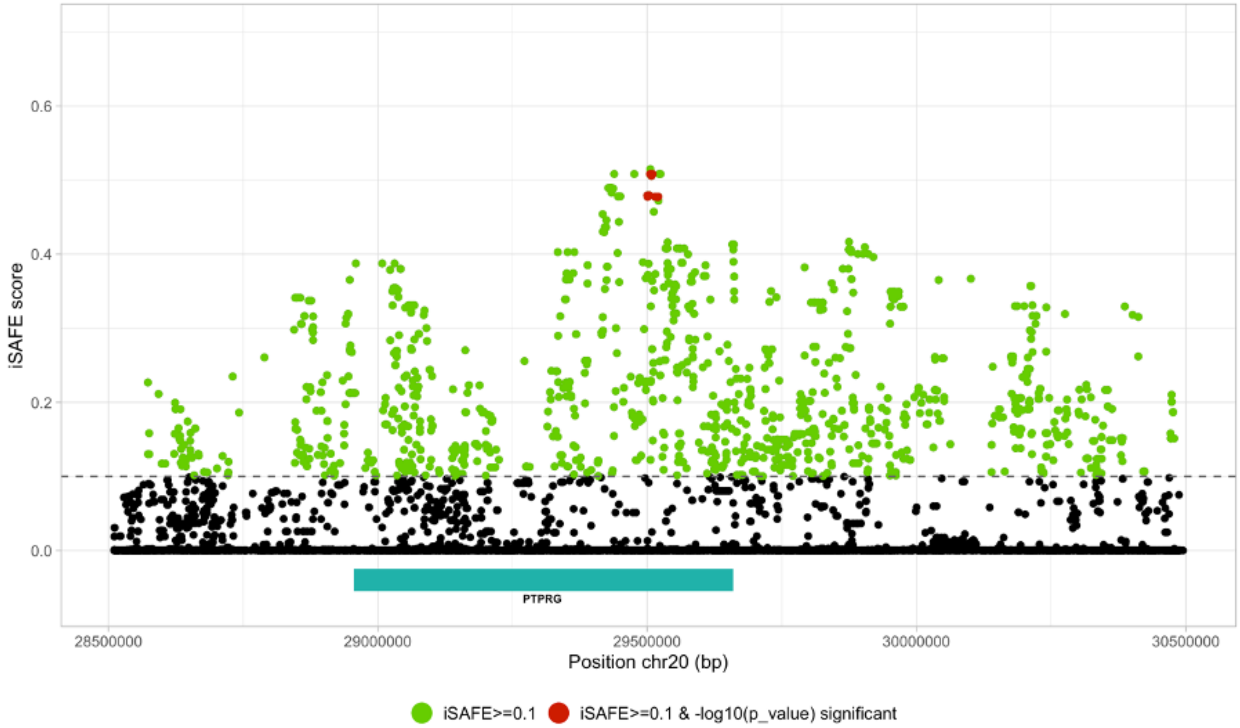

chr38:21631003-21703958

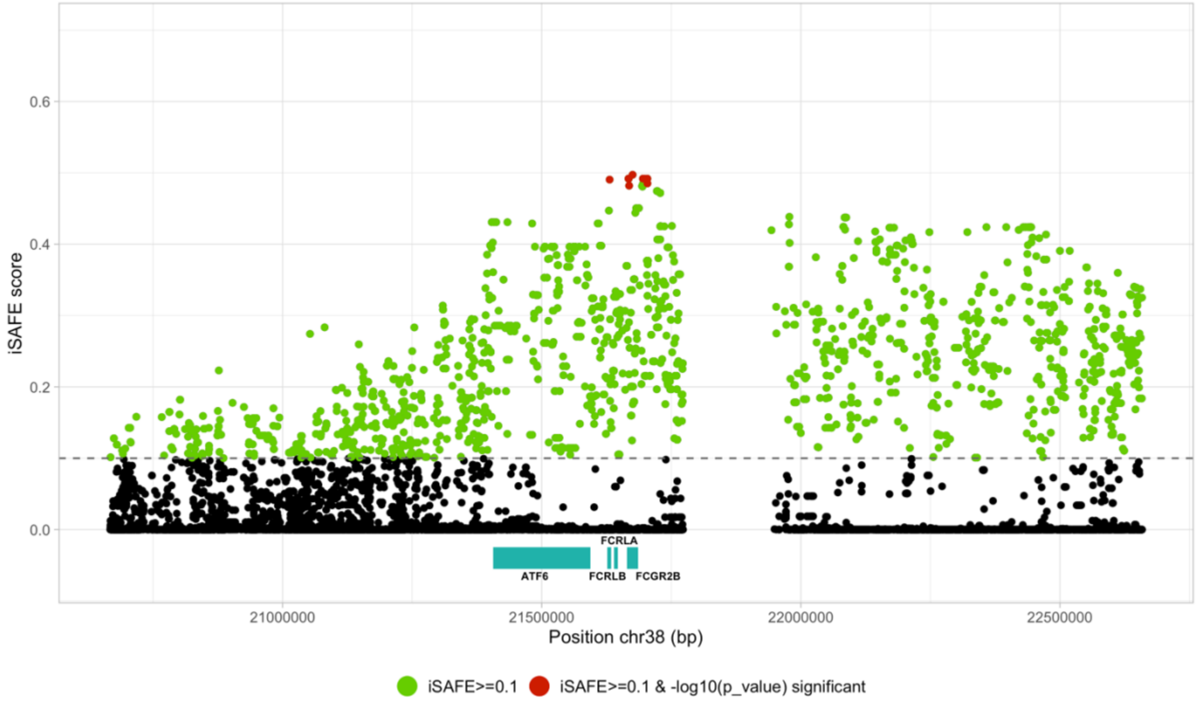

Section 9. Strict filtering

By: Vidhya Jagannathan, Christophe Hitte, Reuben Buckley, Julia E. Niskanen, Matthew J Christmas, Chao Wang, Matteo Bianchi, Jennifer R. S. Meadows

A series of additional filters were applied to the available set of 1,971 samples previously processed Firstly, coyotes were removed to restrict the sample set to wolves, village-, breed- and mixed-breed dogs.

For biallelic single variant positions, VCFtools v0.1.16 [151] was used to filter autosomal SNVs based on depth (--minDP 5) and genotype quality (--minGQ 20). An in-house allelic balance (0.30≤AB≤0.70) filter based on the vcf4.2 allele depth (AD) INFO field was applied, prior to the iterative steps of removing variant positions with >20% genotype missingness (--geno 20) and then samples with >10% missingness (--mind 10) with PLINK v1.90b6.9 (24). Individuals were not removed based on heterozygosity. For biallelic chromosome X variants, male and female samples which passed autosomal filters, were processed separately. For females, --minGQ 20, DP 5 and 0.70 ≥ AB ≤ 0.30 filters were applied, whereas for males, only the genotype quality filter was used and heterozygous variants within the PAR were set to missing. Variant positions were filtered from chromosome X so that the remaining genotyping rate per sample on this chromosome was >90%. In combination, the filters result in a final sample set of 1,929 individuals (1,591 breed or "other" dogs, 281 village dogs and 57 wolves), 27,878,354 autosomal and 847,128 chromosome X, polymorphic biallelic variants. The genotyping rate in the final data set is 0.97 in autosomes and 0.99 for chromosome X.

A comparison of sites removed during this process showed that most were rare, less than 1% allele frequency, but that common sites were also impacted by this filter (*Inline figure 9.1.*). We note that as a validated truth set does not exist for dogs, it is likely that not all variants that PASS VQSR will be true, but given that only 0.7% of those available were removed, that the vast majority are.

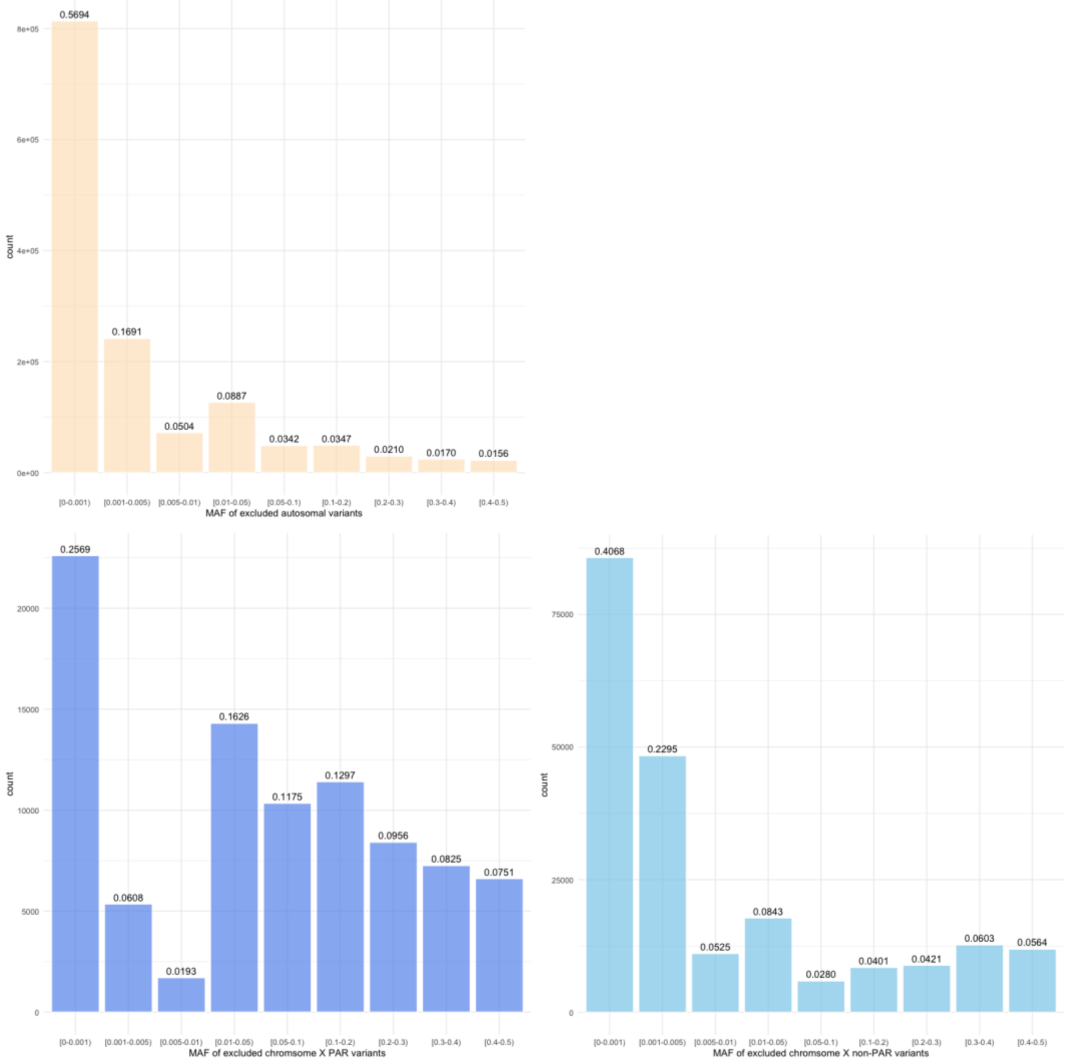

Inline figure 9.1. Allele frequency distribution of sites removed during the strict filtering process.

Section 10. Variant concordance

By: Reuben Buckley and Julia E. Niskanen

The process for genotype concordance is illustrated in the *inline figure 10.1*. A total of 168 Dog10K samples (NHGRI *n* =134, University of Helsinki *n* = 34) were genotyped within Dog10K and previously for separate projects on the 170K Illumina CanineHD array.

First, liftOver was used to convert canFam3.1 Illumina CanineHD array (noted as Array) positions to UU_Cfam_GSD_1.0 (GSD_1.0) coordinates. For the 168 samples considered, the GSD_1.0 Array coordinate sites were extracted using BCFtools v1.13 from the Dog10K VQSR PASS VCF, retaining those positions with at least one non-reference allele. GSD_1.0 genotypes that mapped to the minus strand of canFam3.1 were reverse complemented. Second, for the Array genotype set, monomorphic sites were removed, and sites were reverse complemented when the canFam3.1 reference allele was not present among the alleles called. Merging of these data sets left 151,198 common sites for evaluation, or 145,271 if the sites passing strict filtering (See *Section 9*) were considered.

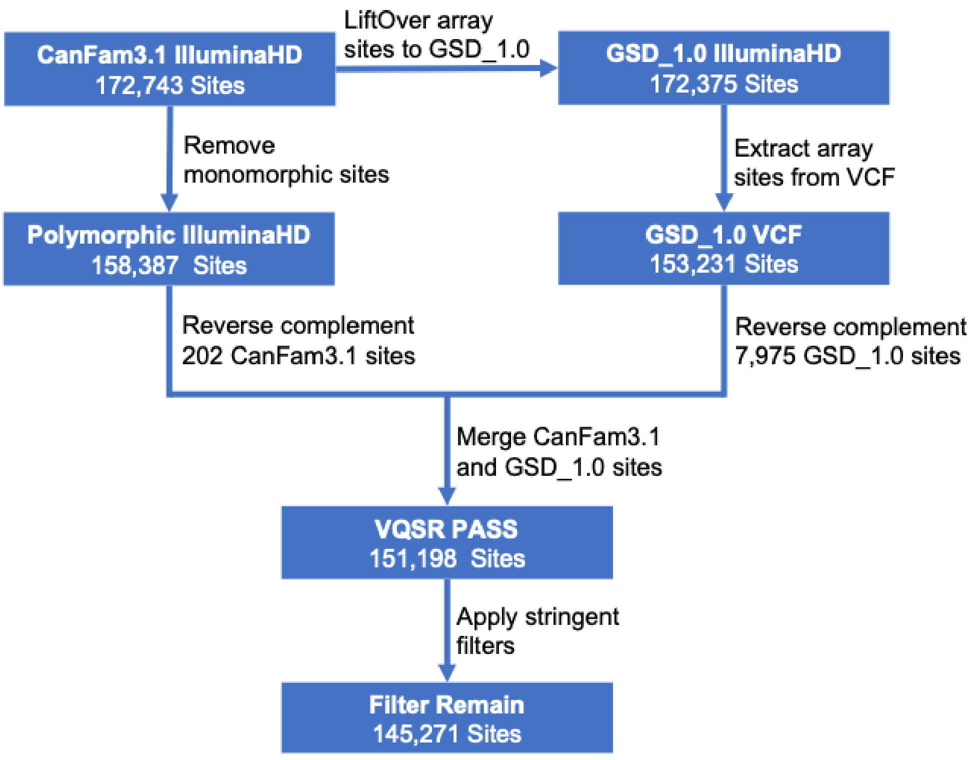

Inline figure 10.1. Site selection for measuring genotype concordance

To avoid confounding due to reference allele differences between CanFam3.1 and UU_Cfam_GSD_1.0, concordance was measured according to the alleles called by each technology, rather than ref/alt genotypes, As such a genotype was considered concordant if it consisted of the same alleles in each dataset, and discordant if both alleles were called, and were different between datasets. Instances where genotyping information was missing in one or both datasets were also recorded.

When considering all sites available, irrespective of if genotypes were missing from the array or for VCF data, concordance was high for both VQSR PASS (97.8%) and Strict (94.6%) data sets (*inline table 10.1*).

Inline table 10.1. Genotype comparisons for 168 samples with Dog10K VCFs and IluminaCanineHD data.

	VQSR PASS[1]	Strict Filter Retained[2]	Strict Filter Removed[3]
**Concordant**	24,845,003 (97.8 %)	22,910,552 (94.6 %)	1,036,410 (87.3 %)
**Discordant**	103,705 (0.41 %)	25,368 (0.10 %)	43,012 (3.62 %)
**Missing in CanineHD**	411,074 (1.62 %)	325,456 (1.34 %)	68,826 (5.80 %)
**Missing in WGS**	36,630 (0.14 %)	934,568 (3.86 %)	35,428 (2.98 %)
**Missing in Both**	4,852 (0.02 %)	18,400 (0.08 %)	3,244 (0.27 %)
**Total**	25,401,264	24,214,344	1,186,920

[1]Genotypes available from VQSR PASS VCF sites.
[2]Genotypes available from Strict filtering VCF sites.
[3]Genotypes removed between VQSR PASS and Strict filtering VCF sites.

When missing sites were removed from consideration, the proportion of non-missing genotypes rose to above 99% (*inline table 10.2*).

Inline table 10.2. Concordance rates for non-missing genotypes.

	VQSR PASS[1]	Strict Filter Retained[2]
**Concordant**	99.58%	99.89%
**Discordant**	0.42%	0.11%

[1]Rate from genotypes available from VQSR PASS sites.
[2]Rate from genotypes available from strict filtering sites.

To determine the likelihood of encountering discordances between datasets, we measured the proportion of sites, per number of discordances. We found that >70% of sites had no discordant samples, and at least 97% of sites had less than three discordant samples (*inline figure 10.2A*), indicating that sites discordant across a high number of samples are rare. We also found that discordant genotypes were relatively evenly distributed across the genome (*inline figure 10.2B*), and that most samples had similar rates for concordant, discordant, and missing genotypes (*inline figure 10.2C*).

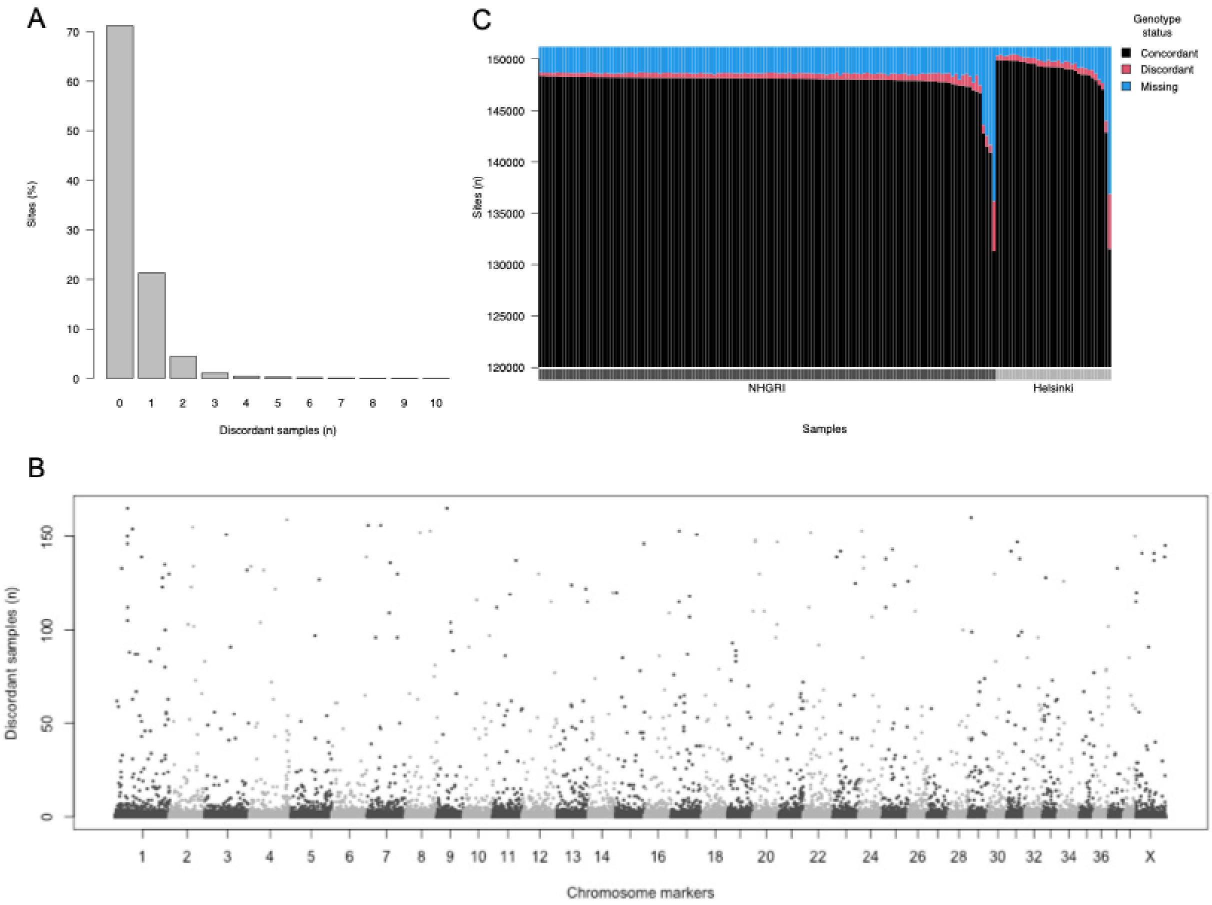

Inline figure 10.2. The landscape of Dog10K WGS SNV and Illumina CanineHD array genotype concordance. **A)** Distribution of sites per number of discordant samples. X-axis has been truncated at 10 discordant samples per site. **B)** Genomic distribution of genotype discordances. Sites are plotted according to chromosome and marker order in GSD_1.0. **C)** Genotype concordance counts per sample. Missing genotypes are missing in either Dog10K VCF or the Illumina CanineHD array. All figure panels displayed are based on the VQSR PASS VCF set of sites and genotypes.

Four samples from the NHGRI, and two samples from the University of Helsinki, had > 7,000 missing genotypes in either of the two platforms, while all other samples had < 4,000 missing genotypes. These six samples contributed to 13.5% of the total number of discordant genotypes and only 3.34% of the overall number of concordant genotypes. Removing these samples increased the overall concordance rate in VQSR PASS VCF from 97.8% to 98.0%. Together these analyses show that the genotype accuracy of the Dog10K VCF call sets are extremely high, and that the Dog10K VCF SNV catalogs are appropriate for use in large-scale genomic analyses.

Section 11. Comparison of public variation catalogs

By: Chao Wang, Jennifer R. S. Meadows

The strict-filtered Dog10K collection was compared to two other publically available datasets in terms of, i) methods used to call variants, ii) sharing of individuals between sets and iii) sharing of breed types. Only positions variable in dogs and wolves were considered. The public datasets were DBVDC (590 samples, 20,443,472 SNVs) [6], and NIH (715 samples, 18,468,060 SNVs) [4]. CanFam3.1 referenced datasets were lifted to UU_Cfam_GSD_1.0 coordinates, with variants on unplaced scaffolds excluded from further analysis. For i) the methods and filters used to call variants are summarized in Additional File 1: Table S12. For ii) individuals were considered shared between datasets if their proportion of IBD was in excess of that observed for the closest pair in Dog10K (i.e. PLINK (v1.9) [176] PiHAT > 0.9451 based on 145,845 random variants, *inline figure 11.1*). For iii) breed types, breed names and descriptors were harmonized, and compared across sets (*inline figure 11.2*, Table S13).

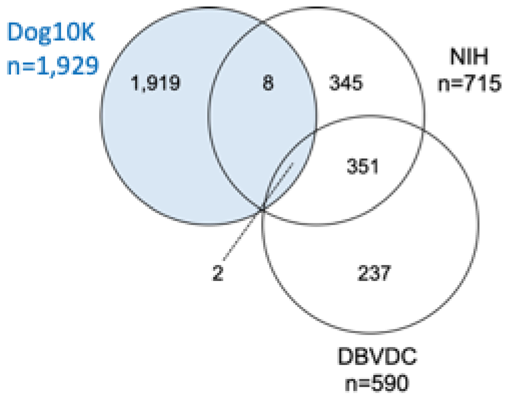

Inline figure 11.1. Samples shared between three large datasets based on proportion of IBD. Total number of samples per dataset is indicated.

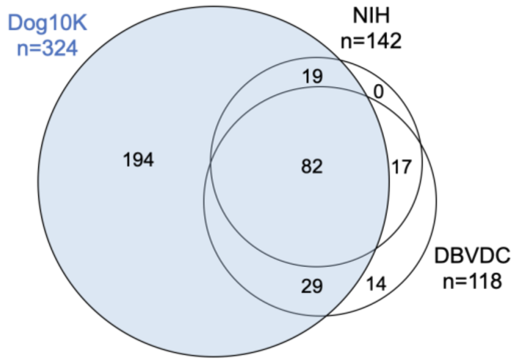

Inline figure 11.2. Breed types shared between three large datasets are indicated. Total number of breed types per dataset is indicated. Breed types are collated in Additional File 1: Table S13. Where information was available, breed types are differentiated so that users can access the panel best suited to their needs (e.g., for Poodle, four types are included: Poodle, Miniature, Toy, Standard). Samples classified as "Unknown" breed are excluded.

Section 12. Genome-wide distribution of genetic variation

By: Christophe Hitte, Matteo Bianchi, Jennifer R. S. Meadows

The genome was divided into 100 kb bins, and SNV allele density calculated for the whole data set (n = 1,929) and each sample set (Breed Dog And Other n = 1,591, Village Dog n = 281, Wolf n = 57) based on regions of the genome (coding or non-coding) and allele frequency bin (rare, AF ≤ 1%; Intermediate, 1% > AF < 5%; common, AF ≥ 5%). To aid visualization, the distribution was Z-transformed. Similar patterns were observed for all four analyses (All, Village Dog, Wolf, *inline figures 11.1-3* respectively).

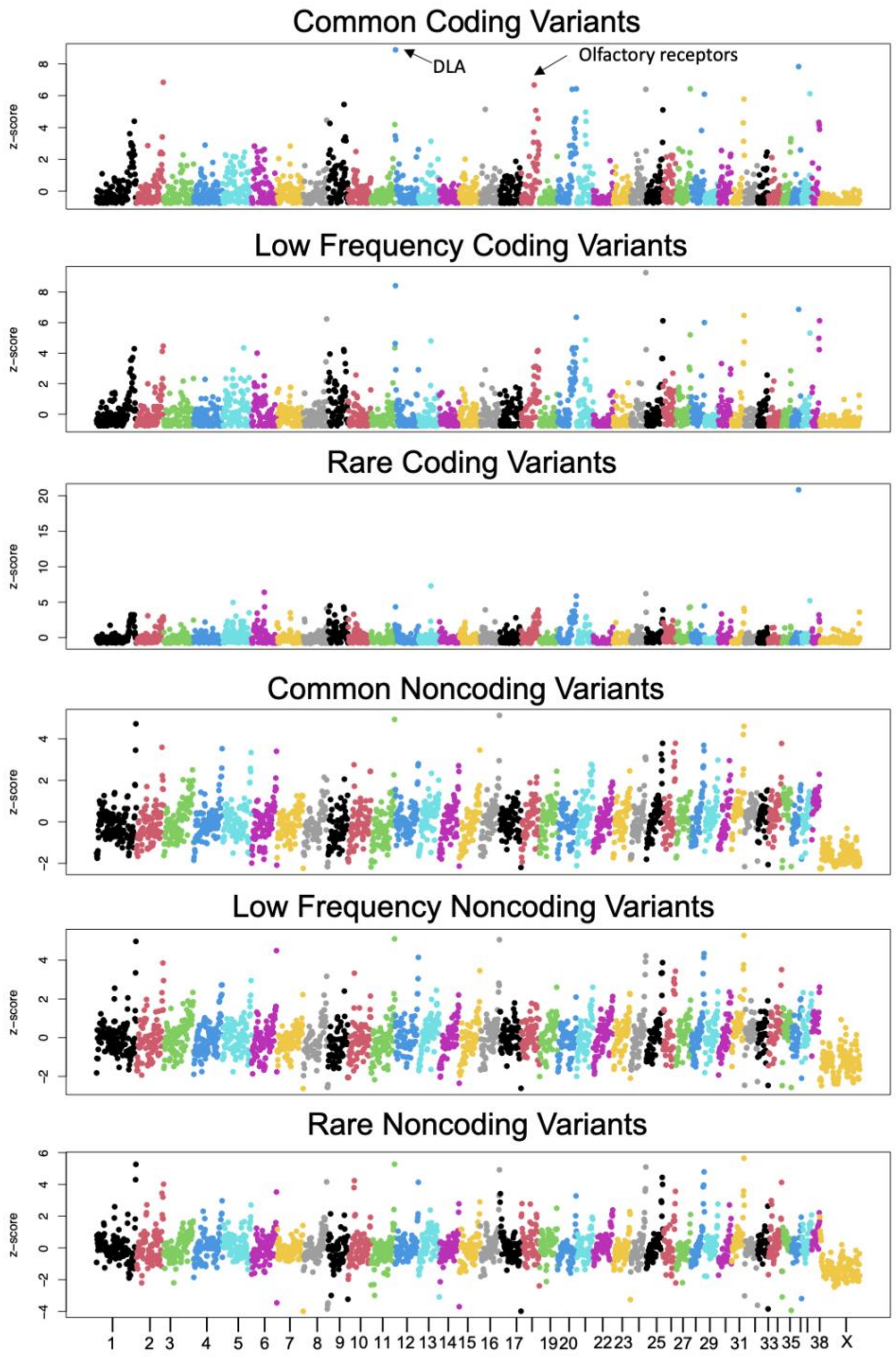

Inline figure 11.1. Distribution of variation across the genome for all samples (n=1,929).

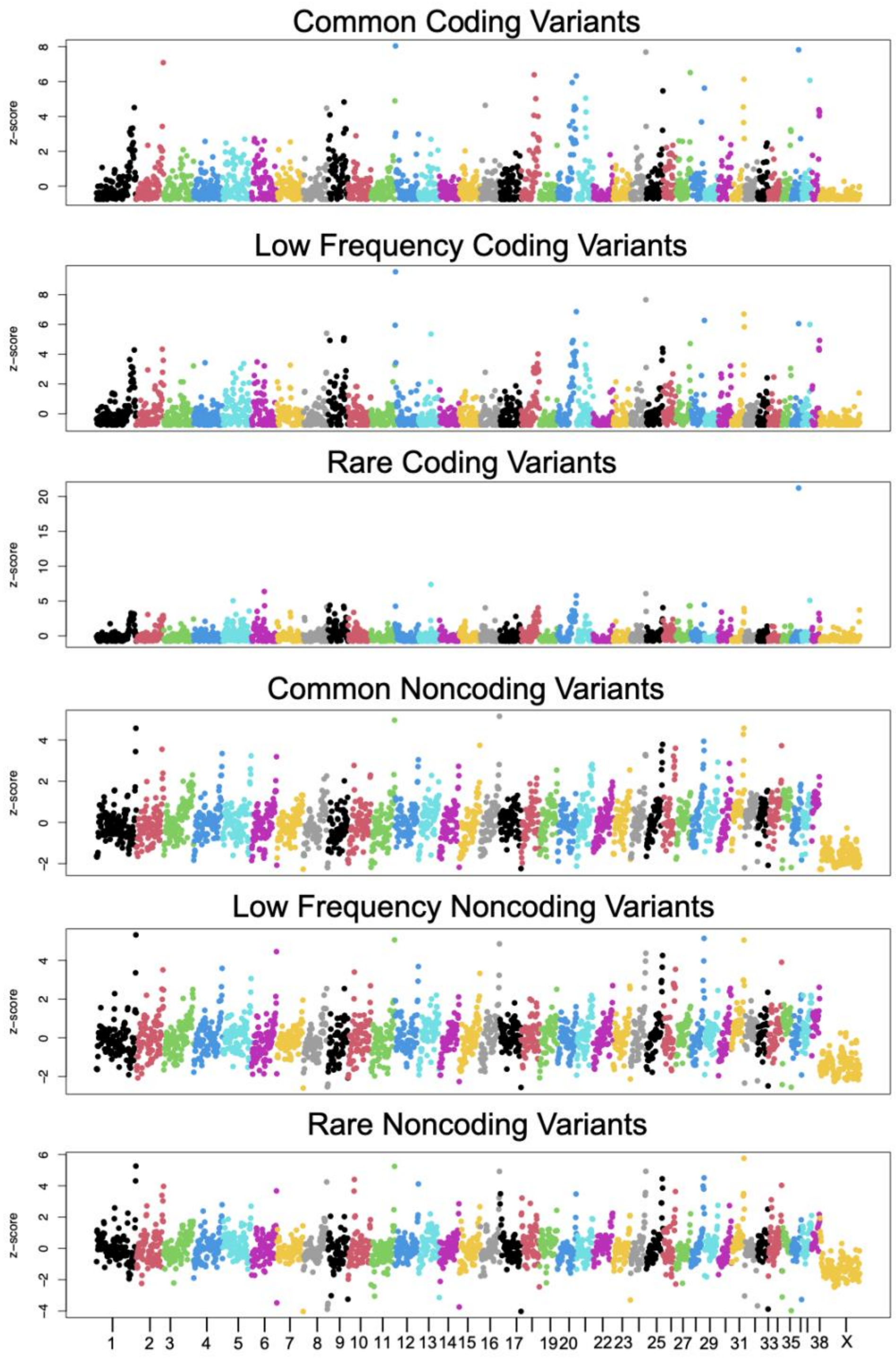

Inline figure 11.2. Distribution of variation across the genome for Village Dogs (n=281).

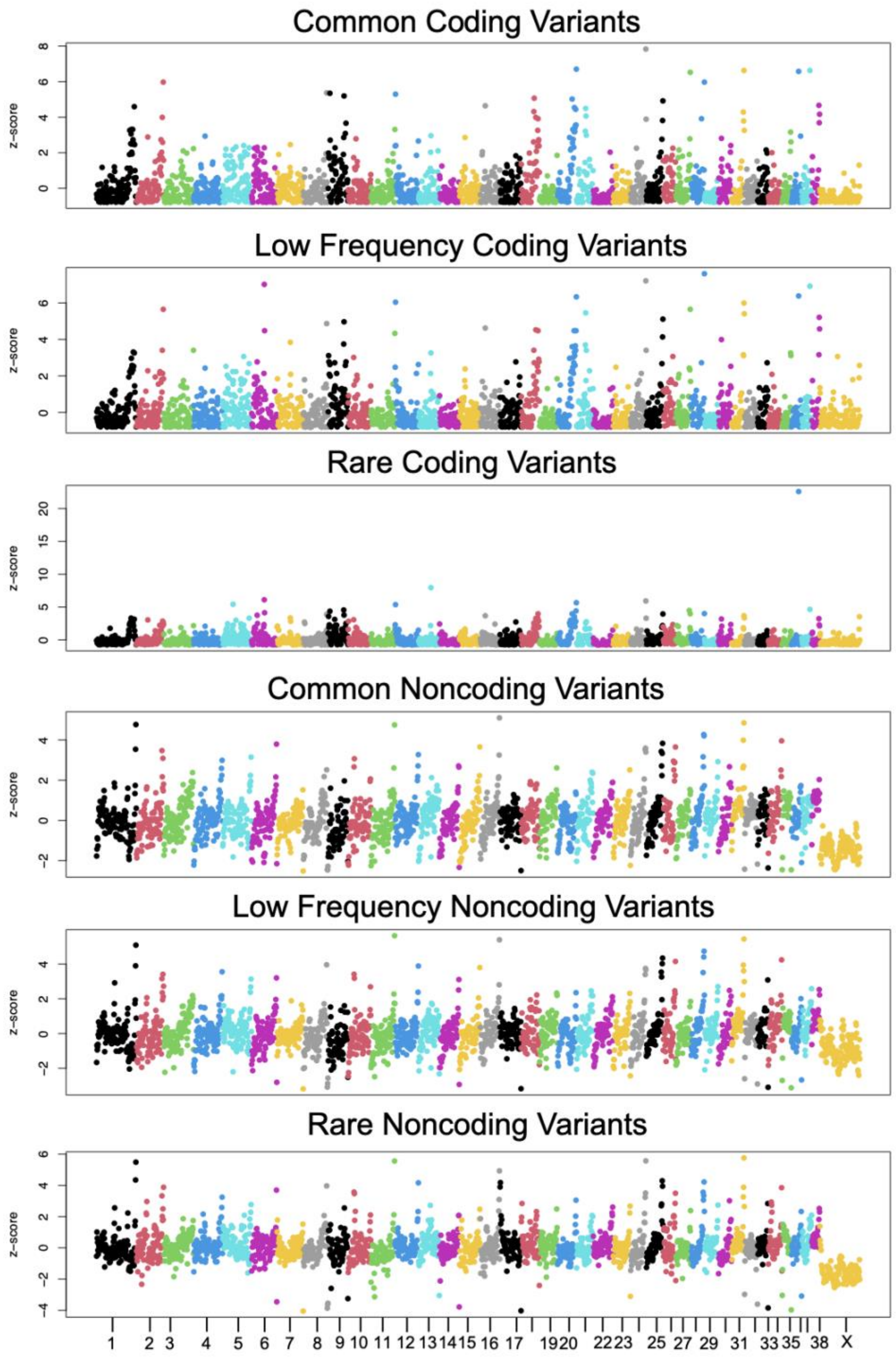

Inline figure 11.3. Distribution of variation across the genome for wolves (n=57).

Section 13. Druggable gene target analysis

By: Anthony K. Nguyen, Peter Z. Schall, Jeffrey M. Kidd, Jennifer R. S. Meadows, Elaine A. Ostrander

We analyzed a set of previously identified druggable gene targets (Tier 1 Genes, n=1,427 genes [99]) for copy number differences in the Dog10K dataset using QuicK-mer2. The identified Tier 1 genes represent efficacy targets of approved small molecules or drugs as well as clinical-phase drug candidates. 176 genes were removed from analysis because they were smaller than a QuicK-mer2 window or were not present in the UU_Cfam_GSD_1.0 annotation, leaving 1,251 genes to examine. We found that 79 of these genes have a copy-number range greater than 2 across the analyzed samples, indicating that they are copy number variable. The ten genes with the largest median CN ranges are listed below in the *inline table 12.1*.

Inline table 12.1. Tier 1 druggable gene targets with the largest copy number range across Dog10K samples.

**Gene Name**	**Coordinates**	**Copy Number Range**
CYP1A2	chr30:38258389-38264108	5.9
CELA1	chr27:43094493-43113139	4.5
SLC28A3	chr1:75622824-75700238	4.3
CFD	chr20:58265041-58267518	4.2
AURKC	chr1:101592105-101595738	3.8
FGF4	chr18:48869443-48873311	3.6
TUBB2A	chr35:4734869-4739525	3.2
MTNR1B	chr21:7715221-7727804	3.1
HTR1A	chr2:49236031-49237302	3.1
HCAR2	chr26:6976781-6978885	3.1

CYP1A2 and *SLC28A3* are discussed in the main text. *AURKC* does not have clearly estimated copy number states, instead having a majority of samples within the copy number range of 1-3 (*inline figure 12.1*). *AURKC* encodes the aurora kinase C protein which assists in cell division regulation. All 9 members of the Chinook breed (COOK) are estimated to carry a duplication of this gene. Additionally, a few samples appear to have deletion of *AURKC*, including a Barbet (BRBT000005) and a Cesky Terrier (CSKT000006).

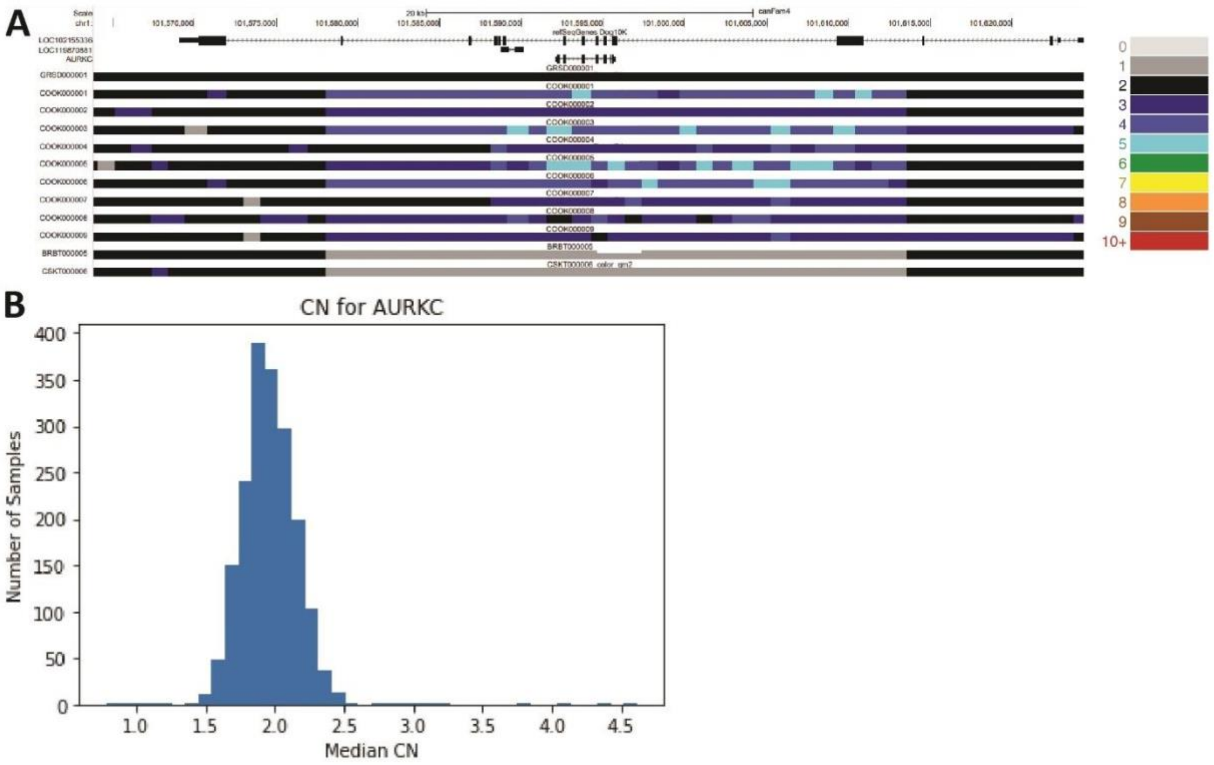

Inline figure 12.1. Copy number analysis of AURKC. (A) A UCSC Genome Browser view showing estimated copy number for the region around *AURKC* is depicted. Colored bars depict copy number predicted by QuicK-mer2 (see heatmap color key to right). Samples include GRSD000001 (a German Shepherd), all members of the Chinook breed (COOK), a Barbet (BRBT), and a Cesky Terrier (CSKT). Blue bars indicate duplicated regions, where all COOK samples show an increased copy number, whereas light grey indicates deleted regions (BRBT and CSKT samples). (B) A histogram showing the estimated copy number for all 1,879 of samples for *AURKC*. Most samples congregate at a copy number of 2, but there are some that appear to have a duplication or deletion.

TUBB2A encodes a beta-tubulin and is involved in mitosis and cellular transport. A substantial number of samples (n=96) have an estimated copy number of 1 or less at this locus; with the samples clustering around an estimated copy number of 0.5 (*inline figure 12.2*). This suggests a partial deletion event, as the copy number was estimated over the entire gene. Examination of the copy number profile reveals a deletion that overlaps the 5’ end of *TUBB2A* as well as large duplications that encompass the entire region.

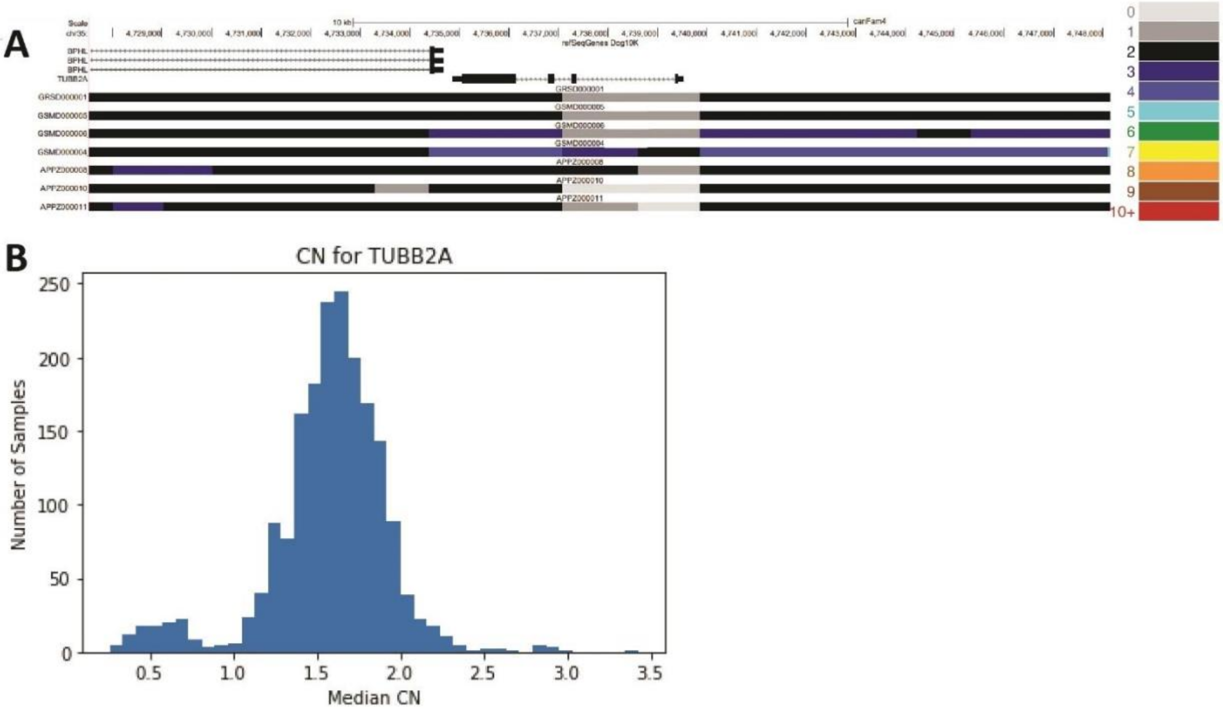

Inline figure 12.2. Copy number analysis of TUBB2A. (A) A UCSC Genome Browser view showing estimated copy number for the region around *TUBB2A* is shown. Colored bars depict copy number predicted by QuicK-mer2 (see heatmap color key to right). Samples include GRSD000001 (a German Shepherd), three Greater Swiss Mountain Dogs (GSMD), and three Appenzeller Sennenhunds (APPZ). Only a single canine, GSMD000004, carries a duplication of this entire region; most dogs have a partial deletion, shown by the light gray in all other samples. (B) A histogram showing the estimated copy number for all 1,879 samples for *TUBB2A*. GSMD000004 is the sample on the far right of the histogram with the largest estimated copy number. The concentrated peaks around 0.5 and 1.5 indicate a partial deletion across the gene locus.
